# Salinirifamycins A–E: Rifamycin S Derivatives from the Brazilian Marine Actinomycete *Salinispora arenicola*

**DOI:** 10.1021/acs.jnatprod.5c01381

**Published:** 2026-01-01

**Authors:** Alison Batista da Silva, Francisco Chagas L. Pinto, Edilberto R. Silveira, Tercio de Freitas Paulo, Diego V. Wilke, Elthon G. Ferreira, Leticia V. Costa-Lotufo, Kirley M. Canuto, José Delano Barreto Marinho-Filho, Ayslan B. Barros, Genoveffa Nuzzo, Angelo Fontana, Norberto Kássio. V. Monteiro, Otilia D. L. Pessoa

**Affiliations:** † Departamento de Química Orgânica e Inorgânica, 28121 Universidade Federal do Ceará, Fortaleza, Ceará 60.021-970, Brazil; ‡ Núcleo de Pesquisa e Desenvolvimento de Medicamentos, 67794 Universidade Federal do Ceará, Fortaleza, Ceará 60.430-275, Brazil; § Departamento de Farmacologia, Universidade de São Paulo, São Paulo, São Paulo 05508-900, Brazil; ∥ Embrapa Agroindústria Tropical, Fortaleza, Ceará 60.511-110, Brazil; ⊥ Núcleo de Pesquisa e Pós-graduação, 603028 Universidade Federal do Delta do Parnaíba, Parnaíba, Piauí 64202-020, Brazil; # 201790 CNR, Istituto di Chimica Biomolecolare, Bio-Organic Chemistry Unit, Pozzuoli, Naples 80078, Italy; ∇ Departamento de Química Analítica e Físico-Química, Universidade Federal do Ceará, Fortaleza, Ceará 60020-181, Brazil

## Abstract

Five new rifamycin derivatives, named salinirifamycins A–E (**1**–**5**), were isolated from a Brazilian marine *Salinispora arenicol*a (BRA-213) strain extract. The structures of the new rifamycins were elucidated using a combination of NMR, IR, UV, and MS spectroscopic techniques, quantum-chemical calculations (DFT-calculated ^13^C NMR chemical shifts and DP4+ probability analysis), and comparison of experimental and calculated electronic circular dichroism (ECD) spectra. Compounds **1**, **2**, and **4** displayed antibacterial activity against *Staphylococcus aureus* and *Enterococcus faecalis* with MIC values ranging from 2.0 to 125.0 μg/mL, whereas **5** exhibited an MIC of 0.02 μg/mL to *S. aureus*, similar to the positive control rifampicin (MIC 0.03 μg/mL).

## Graphical abstract

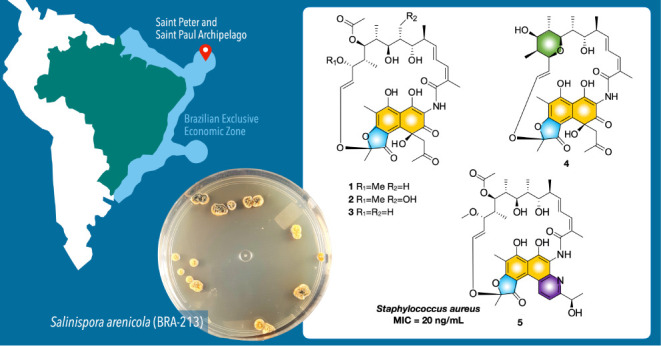

Actinomycetes comprise the main group producing bioactive compounds from an economical and biotechnological point of view.^1^ Due to their great metabolic diversity and bioactivities, those belonging to the genus *Streptomyces* are the most commonly investigated. Species of this genus have been highlighted for producing more than 70% of the total secondary metabolites already reported from actinomycetes.^2^ However, the small genus *Salinispora*, officially documented in 2005 as the first group of exclusively marine actinomycetes, has arisen as a prolific and striking source of new active compounds as well as a model organism in the development of methods for new secondary metabolite discovery.^3^ Recently, the genus, which consisted of just three species, has been expanded to nine species.^4,5^ *Salinispora* species are producers of structurally unusual and highly functionalized compounds such as the salinosporamides,^6^ saliniquinones,^7^ arenicolides,^8^ saliniketals,^9^ and arenamides.^10^ Rifamycins, the ancient and well-known class of antibiotics, have also been reported in *Salinispora* species.^11^ This ancient class of compounds was first isolated in 1957 from *Amycolatopsis mediterranei* S699.^12^ Semisynthetic rifamycin derivatives (e.g., rifampicin) have been widely used in clinical settings, particularly to treat tuberculosis, leprosy, and AIDS-related mycobacterial infections.^13–15^ Continuing with our studies of bioactive natural compounds from marine bacteria,^16,17^ we have now focused our attention on the isolation of rifamycins from the EtOAc extract of *S. arenicola* (BRA-213), since UV bands for these compounds (λ_max_ 230, 268, 318, and 420 nm) were detected in this extract from a preliminary analysis. Herein, the isolation, complete structure characterization, and antimicrobial properties of five novel rifamycin derivatives, designated salinirifamycins A–E (**1**–**5**), are described.
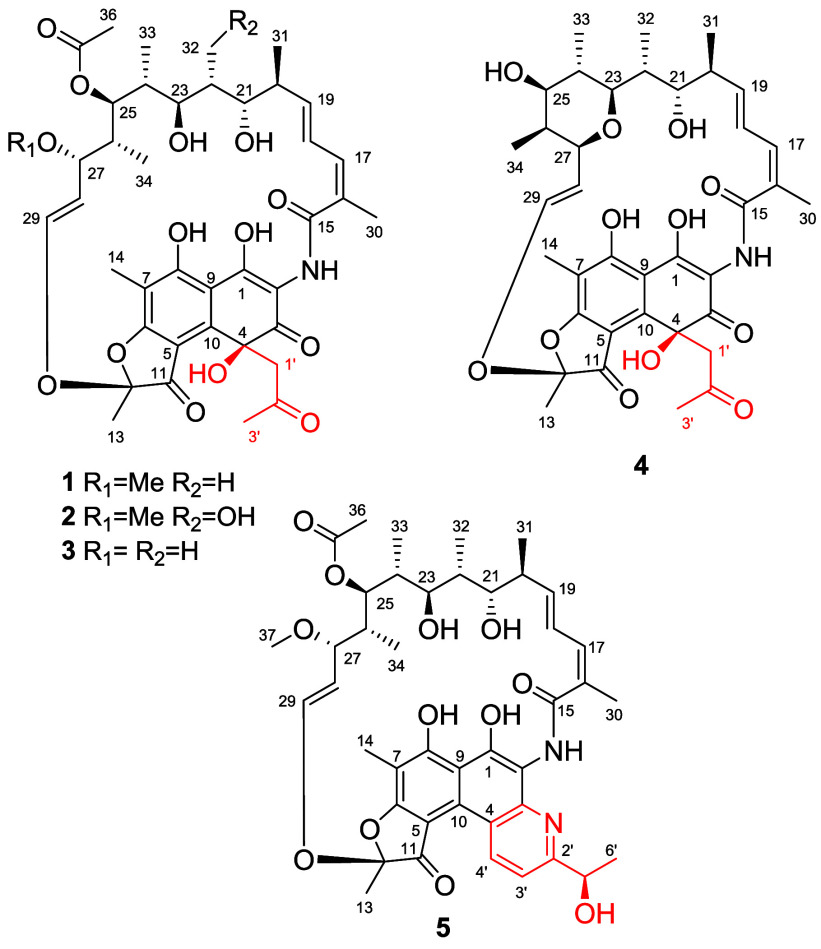


## Results and Discussion

Analysis of the EtOAc extract from the residual aqueous phase of the fermentation broth of *S. arenicola* by LC-MS searching exclusively for compounds with UV bands around λ_max_ 230–420 nm, showed a series of compounds with high molecular weight. Chromatographic procedures, including cartridge C-18, Sephadex LH-20, and reverse-phase HPLC, resulted in the isolation of five new rifamycin S derivatives, named salinirifamycin A–E (**1**–**5**).

Salinirifamycin A (**1**) was isolated as a yellowish solid. Its molecular formula was determined as C_40_H_51_NO_14_ (16 degrees of unsaturation) by HRESIMS data (*m*/*z* 768.3240 [M – H]^−^, calcd for C_40_H_50_NO_14_, 768.3237). Its ^1^H NMR spectrum exhibited signals to five exchangeable protons at δ_H_ 19.15, 8.00, 6.09, 5.00, and 4.00 assigned to NH or OH groups. It is worth highlighting that the unusual chemical shift δ_H_ 19.15 for HO-1 (a β-diketone enol) can be explained by strong hydrogen bonding either with the DMSO solvent or an intermolecular hydrogen bond with the C-8 hydroxy or with the C-15 carbonyl. The ^1^H and HSQC NMR spectra showed five vinyl protons at δ_H_ 7.07 (dd, *J* = 15.9, 10.7 Hz, H-18), 6.28 (d, *J* = 12.9 Hz, H-29), 6.09 (d, *J* = 10.7 Hz, H-17), 5.93 (dd, *J* = 15.9, 7.6 Hz, H-19), and 4.90 (dd, *J* = 12.9, 8.6 Hz, H-28), and four oxymethine protons at δ_H_ 5.07 (d, *J* = 10.6 Hz, H-25), 3.86 (br d, *J* = 8.9 Hz, H-21), 3.26 (d, *J* = 8.6 Hz, H-27), and 2.82 (m, H-23). In addition, ten methyl groups, including a methoxy (δ_H_ 2.87, OMe-37), a methyl ketone (δ_H_ 2.16, Me-3′), and an acetyl group (δ_H_ 1.97, Me-36), as well as signals for one pair of diastereotopic methylene protons at δ_H_ 2.81 (d, *J* = 11.9 Hz, H_2_-1′b) and 2.72 (d, *J* = 11.9 Hz, H_2_-1′a), were identified. The ^13^C-APT NMR displayed signals for 40 carbons (Table 1), which were characterized as 10 methyls, one methylene, 13 methines (five olefinic and four oxygenated), and 16 non-hydrogenated carbons (five carbonyls). The chemical shifts of the methyl ketone (δ_H_/δ_C_ 2.16/32.7, Me-3′), the methylene group (δ_H_/δ_C_ 2.81 and 2.73/32.7, C-1′), and the carbonyl carbon at δ_C_ 205.0 (C-2′), along with the HMBC correlations of the Me-3′ and methylene protons (CH_2_–1′) with C-2′, suggested the presence of a 2′-oxopropyl group. Detailed analysis of the ^1^H and ^13^C NMR spectroscopic data (Table 1), along with the COSY, HSQC, and HMBC correlations of **1** (Figure 1), showed the same polyketide chain from the C-15 (δ_C_ 167.7) to C-29 (δ_C_ 143.2) characteristics of rifamycins.^18^ The presence of the fully substituted naphthofuranone core was indicated by the HMBC correlations of Me-13 (δ_H_ 1.60) with the carbons at δ_C_ 193.6 (C-11) and 107.6 (C-12); Me-14 (δ_H_ 1.96) with δ_C_ 175.6 (C-6), 169.2 (C-8), and 105.9 (C-7); and the hydroxy proton at δ_H_ 19.15 (OH-1) with δ_C_ 172.8 (C-1), 106.0 (C-2), and 109.4 (C-9). The polyketide chain was attached to the naphthofuranone moiety at C-2 and C-12 based on the HMBC correlations of the amide proton at δ_H_ 8.00 with the carbons at δ_C_ 188.3 (C-3), 167.7 (C-15), and 106.0 (C-2), as well as the olefinic proton δ_H_ 6.28 (H-29) with the ketal carbon δ_C_ 107.6 (C-12). Finally, the unequivocal position of the 2′-oxopropyl group at C-4 was established by HMBC correlations of methylene protons CH-1′ with the carbons at δ_C_ 188.3 (C-3), 145.1 (C-10), and 76.5 (C-4). The relative configuration of the polyketide chain was suggested to be the same as that of previously reported rifamycins, some of which were identified in *Salinispora* species, and therefore, assumed to share the same biosynthetic origin.^19,20^ Due to the absence of NOE correlation for the hydroxy proton (HO-4, δ_H_ 6.09), the relative configuration at C-4 could not be established. Therefore, calculations of ^13^C NMR chemical shifts using the GIAO-mPW1PW91/6–31G­(d,p) level of theory, combined with DP4+ probability analysis^22,23^ were performed to distinguish between the 4*R* (**1a**) and 4*S* (**1b**) relative configurations. Linear regression analyses for both isomers **1a** and **1b** and the experimental data of **1** were inconclusive, as the correlation coefficients (*R* ^2^) of 0.9927 and 0.9938 were very close. However, the DP4+ probability analysis of 98.81% (Table S1) suggests the relative configuration 4S* (Figure S49). The absolute configuration of all stereocenters was assigned by comparing the predicted electronic circular dichroism (ECD) spectrum to experimental data (Figure S54). The results indicated a good match between the calculated and experimental ECD spectra for the isomer 4*S*, supporting the assignment of the absolute configuration of C-4. Therefore, it was suggested that the absolute configuration of **1** is 4*S*,12*S*,20*S*,21*S*,22*R*,23*R*,24*R*,25*S*,26*R*,27*S*, establishing its structure as a new 4-(2′-oxopropyl)-3-oxorifamycin S derivative.

**Table 1:** ^1^H (600 MHz) and ^13^C (150 MHz) NMR Spectroscopic Data for Compounds **1** and **2** (δ in ppm, *J* in Hz) in DMSO-*d*_6_

	**1**		**2**	
no.	δ_C_, type	δ_H_	δ_C_, type	δ_H_
1	172.8, C		173.0, C	
2	106.0, C		106.0, C	
3	188.3, C		188.1, C^a^	
4	76.5, C		76.4, C^a^	
5	105.7, C		105.7, C	
6	175.6, C		175.5, C	
7	106.4, C		106.5, C	
8	169.2, C		169.3, C	
9	109.4, C		109.4, C	
10	145.1, C		145.2, C	
11	193.6, C		193.6, C	
12	107.6, C		107.5, C	
13	22.2, CH_3_	1.60 s	22.1, CH_3_	1.60 s
14	7.1, CH_3_	1.96 s	7.0, CH_3_	1.96 s
15	167.7, C		167.8, C	
16	132.6, C		132.2, C^*a*^	
17	131.2, CH	6.09 d (10.7)	131.2, CH	6.09 d (10.9)
18	127.5, CH	7.07 dd (15.9, 10.7)	127.6, CH	7.08 dd (16.2, 10.9)
19	137.5, CH	5.93 dd (15.9, 7.6)	137.8, CH	5.94 dd (16.2, 7.7)
20	37.3, CH	2.20 m	37.3, CH	2.38 m
21	72.7, CH	3.86 br d (8.9)	73.1, CH	3.84 br d (10.1)
22	32.3, CH	1.72 m	41.2, CH	1.65 m
23	76.2, CH	2.82 m	71.1, CH	3.24 br t (8.8)
24	39.2, CH	1.46 m	39.9, CH	1.47 m
25	73.7, CH	5.07 d (10.6)	73.8, CH	5.07 d (10.9)
26	38.6, CH	1.44 m	38.3, CH	1.42 m
27	76.3, CH	3.26 d (8.6)	76.4, CH	3.27 d (8.7)
28	117.9, CH	4.90 dd (12.9, 8.6)	117.9, CH	4.91 dd (12.9, 8.5)
29	143.2, CH	6.28 d (12.9)	143.1, CH	6.27 d (12.9)
30	20.3, CH_3_	1.87 s	20.2, CH_3_	1.85 s
31	18.3, CH_3_	0.85 d (6.7)	18.5, CH_3_	0.93 d (6.8)
32a	10.7, CH_3_	0.91 d (7.0)	57.2, CH_2_	3.53 dd (10.8, 8.5)
32b				3.64 dd (10.8, 4.6)
33	9.6, CH_3_	0.65 d (6.7)	9.5, CH_3_	0.62 d (6.8)
34	9.5, CH_3_	–0.08 d (6.6)	9.5, CH_3_	–0.07 d (6.7)
35	169.3, C		169.3, C	
36	20.7, CH_3_	1.97 s	20.7, CH_3_	1.97 s
37	55.6, CH_3_	2.87 s	55.6, CH_3_	2.88 s
1′a	54.9, CH_2_	2.73 d (11.9)	55.1, CH_2_	2.75 d (12.0)
1′b		2.81 d (11.9)		2.80 d (12.0)
2′	205.0, C		205.0, C	
3′	32.7, CH_3_	2.16 s	32.7, CH_3_	2.16 s
HN-2		8.00 s		7.92 s
HO-1		19.15 s		19.11 br s
HO-4		6.09 s		6.10 s
HO-21		5.00 d (3.4)		4.94 br s
HO-23		4.00 d (8.9)		4.03 d (8.7)
HO-32				4.24 br s

a The ^13^C chemical shifts were determined by analysis of the HMBC spectrum.

**Figure 1:**
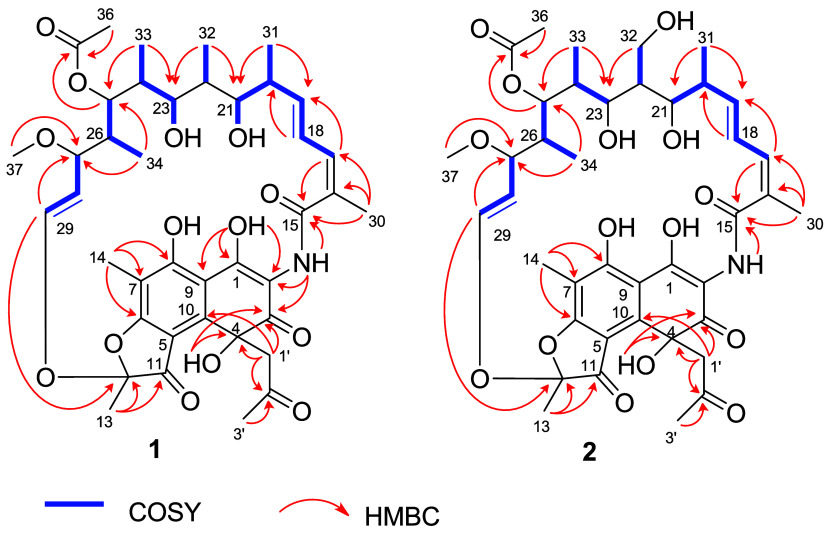
Key COSY and HMBC correlations of compounds **1** and **2**.

Salinirifamycin B (**2**) was isolated as a yellowish solid. Its molecular formula, C_40_H_51_NO_15_, was defined based on HRESIMS, whose spectrum displayed an ion peak [M—H]^−^ at *m*/*z* 784.3205. Analyses of the ^1^H and ^13^C NMR data of **2** (Table 1) showed the presence of a rifamycin S derivative bearing the 2′-oxopropyl moiety at C-4, similar to **1**. The difference between **2** and **1** was the replacement of the methyl group (δ_H_/δ_C_ 0.91/10.7, Me-32) in **1** by a hydroxymethyl group (δ_H_ 3.64, dd, *J* = 10.8, 4.6 Hz, H-32b, 3.53, dd, *J* = 10.8, 8.5 Hz, H-32a; δ_C_ 57.2, C-32) in **2**. The position of the hydroxymethyl group at C-32 was assigned by the HMBC correlation of H_2_-32 (δ_H_ 3.53) with the carbon at δ_C_ 71.1 (C-23) (Figure 1), supporting the structure of **2** as a new 32-hydroxy-4-(2′-oxopropyl)-3-oxorifamycin S derivative. The relative configuration of **2** (**2a**: *R* and **2b**: *S*) was determined similarly to that of **1**. The DP4+ probability results for **2b** show 100% viability, suggesting an *S*-configuration for the stereocenter C-4 (Table S1). In turn, the absolute configuration for **2** was assigned by comparing the experimental and calculated ECD data (Figure S54). Thus, the absolute configuration of **2** was suggested to be identical to that of rifamycin **1**.

Salinirifamycin C (**3**), isolated as a yellowish solid, had its molecular formula assigned as C_39_H_49_NO_14_ based on the [M – H]^−^ ion peak at *m*/*z* 754.3095 (calcd *m*/*z* 754.3080) in the HRESIMS. Comparatively, the NMR spectroscopic data of **3** (Table 2) were similar to those of **1**, except for the shielding of almost 10 ppm of C-27 (**1**: δ_C_ 76.3; **2**: δ_C_ 66.0), consistent with the presence of a hydroxy group instead of a methoxy group in C-27 (Figure 2). The relative configuration of **3** (**3a**: *R* and **3b**: *S*) was also determined similarly to that of **1** and **2**. Based on the results of the DP4+ probability (99.99%) for **3b**, an *S*-configuration for the stereocenter C-4 was suggested (Table S1). Comparison of the ECD experimental and calculated data (Figure S54) for **3** allowed us to determine the absolute configuration of **3** as being identical to rifamycins **1** and **2**.

**Table 2:** ^1^H and ^13^C NMR Spectroscopic Data for Compounds **3** and **4** (*δ* in ppm, *J* in Hz) in DMSO-*d*_6_

	**3** ^a^		**4** ^b^	
no.	δ_C_, type^c^	δ_H_	δ_C_, type^c^	δ_H_
1	nd^d^		173.0, C	
2	nd		105.6, C	
3	188.6, C		188.2, C	
4	76.2, C		76.4, C	
5	nd		nd	
6	175.4, C		175.1, C	
7	105.7, C		105.5, C	
8	169.6, C		169.8, C	
9	nd		109.3, C	
10	nd		145.2, C	
11	194.0, C		194.2, C	
12	107.6, C		107.0, C	
13	21.9, CH_3_	1.58 s	21.3, CH_3_	1.57 s
14	7.0, CH_3_	1.97 s	6.9, CH_3_	1.96 s
15	167.7, C		167.5, C	
16	132.1, C		131.3, C	
17	131.2, CH	6.09 d (11.0)	131.1, CH	6.09 d (10.7)
18	127.5, CH	7.06 dd (16.1, 11.0)	127.1, CH	7.02 dd (16.0, 10.7)
19	137.6, CH	5.93 dd (16.1, 7.6)	137.8, CH	5.93 dd (16.0, 7.7)
20	37.4, CH	2.22 m	37.3, CH	2.22 m
21	73.1, CH	3.85 br d (9.7)	72.7, CH	3.90 br d (9.3)
22	32.4, CH	1.72 m	32.3, CH	1.74 m
23	76.3, CH	2.86 br t (8.7)	76.1, CH	3.21 br t (9.7)
24	38.9, CH	1.45 m	38.6, CH	1.32 m
25	74.4, CH	5.02 d (11.0)	69.5, CH	3.53 dd (10.3, 6.4)
26	39.4, CH	1.37 m	40.2, CH	1.11 m
27	66.0, CH	3.77 m	65.4, CH	4.30 br t (8.6)
28	123.0, CH	5.08 dd (12.6, 7.5)	124.5, CH	5.13 dd (12.7, 6.5)
29	140.8, CH	6.16 d (12.6)	139.3, CH	6.07 d (12.7)
30	20.4, CH_3_	1.85 s	20.2, CH_3_	1.85 s
31	18.3, CH_3_	0.86 d (6.5)	18.1, CH_3_	0.86 d (6.7)
32	10.9, CH_3_	0.91 d (7.0)	10.7, CH_3_	0.93 d (7.0)
33	9.5, CH_3_	0.65 d (6.7)	8.5, CH_3_	0.54 d (6.7)
34	9.2, CH_3_	–0.11 d (6.5)	9.0, CH_3_	–0.22 d (6.6)
35	170.2, C			
36	20.9, CH_3_	1.95 s		
1′a	55.0, CH_2_	2.73 d (12.0)	55.1, CH_2_	2.73 d (11.9)
1′b		2.81 d (12.0)		2.80 d (11.9)
2′	205.2, C		205.1, C	
3′	32.9, CH_3_	2.16 s	32.4, CH_3_	2.16 s
HN-2		8.01 s		7.94 s
HO-1				19.10 s
HO-4		6.07 s		6.00 s
HO-21		5.00 br s		4.90 d (3.7)
HO-23		4.03 d (8.5)		
HO-25				3.99 d (4.1)
HO-27		3.99 d (4.0)		

a 500 MHz (^1^H) and 125 MHz (^13^C).

b 600 MHz (^1^H) and 150 MHz (^13^C).

c The ^13^C chemical shifts were determined by analysis of 2D NMR spectra.

d nd: not detected.

**Figure 2:**
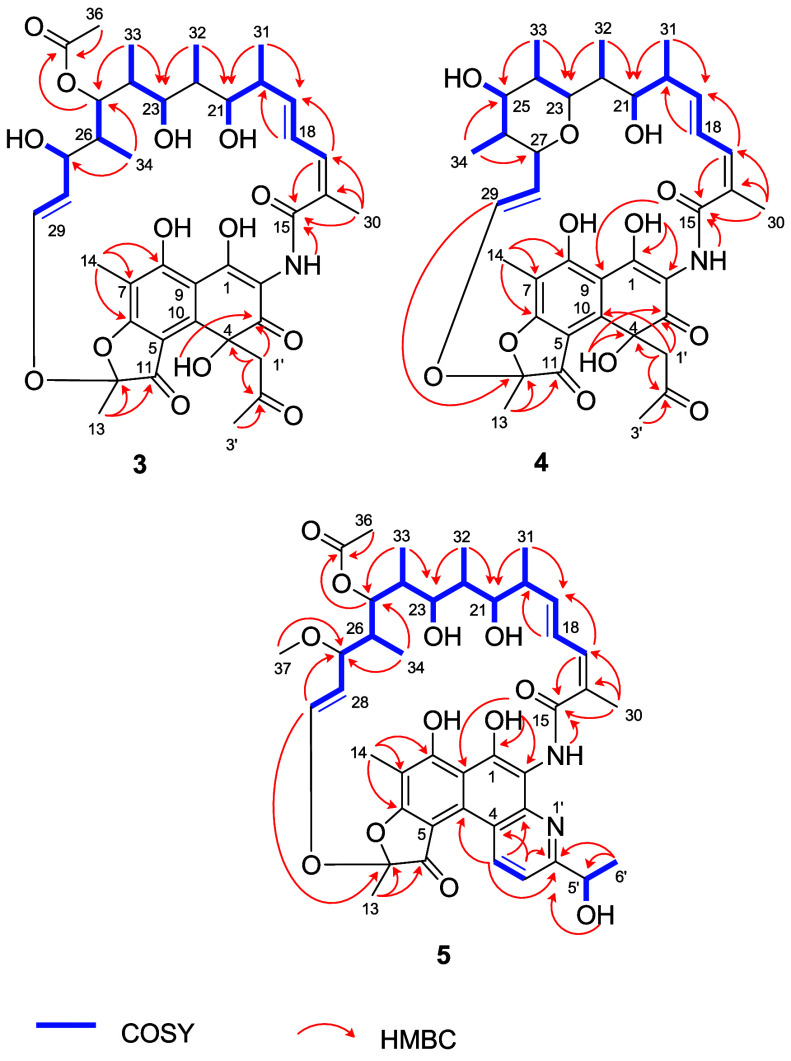
Key COSY and HMBC correlations of compounds **3**, **4**, and **5**.

Salinirifamycin D (**4**) was also isolated as a yellowish solid. Its molecular formula was determined as C_37_H_45_NO_12_ by HRESIMS data (*m*/*z* 694.2872 [M – H]^−^, calcd for C_37_H_44_NO_12_, 694.2869). Analyses of the ^1^H and ^13^C NMR data of **4** (Table 2) also showed the presence of the rifamycin S derivative bearing the 2′-oxopropyl group at C-4, similar to **1** but without the acetyl and methoxy groups found in **1**. The sequence of scalar couplings for the spin systems Me-33­(C-24)–H-23, Me-34­(C-26)–HO-25, and H-29–H-27 observed in the COSY spectrum, along with the HMBC correlations of the Me-33 (δ_H_ 0.54, d, *J* = 6.7 Hz) with the carbons at δ_C_ 76.1 (C-23) and 69.5 (C-25) and Me-34 (δ_H_-0.22, d, *J* = 6.6 Hz) with δ_C_ 69.5 (C-25) and 65.4 (C-27), support the presence of the ether group (C-23/O/C-27) as depicted in Figure 2.

The relative configuration of **4** was determined similarly to the above rifamycins **1**–**3**, but with a focus on the C-4, C-23, and C-27 stereocenters. DP4+ calculations for the isomers **4a** (4*R*, 23*R*, 27*S*), **4b** (4*S*, 23*R*, 27*S*), **4c** (4*R*, 23*S*, 27*R*), and **4d** (4*S*, 23*S*, 27*R*) indicated a perfect match ratio of 100% for the **4b** isomer (Table S1). The absolute configuration of **4** was assigned as 4*S*,12*S*,20*S*,21*S*,22*R*,23*R*,24*R*,25*S*,26*R*,27*S* by comparison between the experimental and predicted ECD spectra (Figure S54). Therefore, the structure of **4** was established as 27-demethoxy-27-hydroxy-4­(2′-oxopropyl)-3-oxorifamycin S.

Salinirifamycin E (**5**), obtained as a red powder, had its molecular formula C_42_H_52_N_2_O_12_ determined based on the protonated molecule [M + H]^+^ ion at *m*/*z* 777.3597 (calcd for C_42_H_53_N_2_O_12_, 777.3593), indicating 18 degrees of unsaturation. The ^1^H and ^13^C NMR data of **5** (Table 3) showed resonances that belong to a polyketide side chain similar to that found in **1**. Rifamycins behave like paracyclophane molecules, where the positioning of the protons relative to the benzene ring of the naphthopyrone moiety can cause a higher shielding effect on the methyl group just above the π-electron ring current, as was observed for the methyl-34 of compounds **1**–**4** (δ_H_ −0.07 to −0.22). On the other hand, compound **5** has two shielded methyls at δ_H_ −0.11 (Me-33) and −0.69 (Me-34). This can be explained by a molecular model showing C-33 above the naphthalene portion of the naphthofuranone, while C-34 is directed upward toward the pyridine ring. Additionally, its ^1^H NMR spectrum exhibited two deshielded signals at δ_H_ 8.50 (H-4′) and 8.23 (H-3′), both appearing as a doublet with a coupling constant (*J*) of 8.9 Hz, indicating *ortho*-positioned protons of a pyridine ring moiety,^21^ an extra oxygen-bearing methine proton at δ_H_ 5.33 (m, H-5′), and a methyl at δ_H_ 1.59 (d, *J* = 6.6 Hz, Me-6′) assigned to a 1-hydroxyethyl moiety. The fully substituted naphthofuranone core was established by the HMBC correlations of the Me-13 (δ_H_ 1.83) with the carbons at δ_C_ 188.9 (C-11) and 108.2 (C-12); Me-14 (δ_H_ 2.09) with the carbons at δ_C_ 182.1 (C-8), 171.3 (C-6), and 105.9 (C-7), and a chelated hydroxy proton at δ_H_ 18.02 (OH-1) with δ_C_ 157.9 (C-1), 111.0 (C-2), and 116.3 (C-9). Additional proton and carbon signals at δ_C_/δ_H_ 156.5, 138.7/8.50, 120.3/8.23, 66.0/5.33, and 22.6/1.59 were compatible with a fused pyridine ring substituted by a 1-hydroxyethyl group. The presence of the vicinally coupled protons H-3′ (δ_H_ 8.23)/H-4′ (δ_H_ 8.50), both exhibiting *J* = 8.9 Hz, together with the HMBC correlation of H-4′ to δ_C_ 110.5 (C-10), was crucial to determining the unequivocal position of the pyridine ring at C-3/C-4 of the naphthofuranone core (Figure 2). HMBC correlations of H-4′ (δ_H_ 8.50), Me-6′ (δ_H_ 1.59), and HO-5′ (δ_H_ 6.46) to δ_C_ 156.5 (C-2′) ensured the 1-hydroxyethyl group position at C-2′.

**Table 3:** ^1^H (500 MHz) and^13^C (125 MHz) NMR Spectroscopic Data for Compound **5** (*δ*in ppm,*J* in Hz) in DMSO-*d*_6_

	**5** ^a^	
no.	δ_C_, type	δ_H_
1	157.9, C	
2	111.0, C	
3	129.1, C	
4	127.2, C	
5	nd^b^	
6	171.3, C	
7	105.9, C	
8	182.1, C	
9	116.3, C	
10	110.5, C	
11	188.9, C	
12	108.2, C	
13	21.1, CH_3_	1.83 s
14	7.3, CH_3_	2.09 s
15	167.8, C	
16	132.7, C	
17	131.6, CH	6.41 d (11.4)
18	125.3, CH	6.81 dd (15.3, 11.4)
19	138.8, CH	6.10 dd (15.3, 7.0)
20	37.6, CH	2.26 m
21	71.9, CH	3.52 br d (9.8)
22	32.2, CH	1.55 m
23	75.2, CH	2.69 br t (9.1)
24	37.5, CH	0.86 m
25	71.7, CH	4.94 d (11.6)
26	39.3, CH	0.43 m
27	75.5, CH	3.22 br d (7.1)
28	118.3, CH	4.99 m
29	141.6, CH	6.19 d (12.6)
30	19.9, CH_3_	2.01 s
31	17.7, CH_3_	0.84 d (7.6)
32	10.7, CH_3_	0.86 d (7.5)
33	8.0, CH_3_	–0.11 d (6.7)
34	7.9, CH_3_	–0.69 d (6.8)
35	169.0, C	
36	20.7, CH_3_	1.90 s
37	55.6, CH_3_	2.88 s
2′	156.5, C	
3′	120.3, CH	8.23 d (8.9)
4′	138.7, CH	8.50 d (8.9)
5′	66.0, CH	5.33 m
6′	22.6, CH_3_	1.59 d (6.6)
HN-2		9.42 s
HO-1		18.02 s
HO-8		18.87 s
HO-21		4.95 br s
HO-23		3.96 d (8.8)
HO-5′		6.46 d (3.2)

a The ^13^C chemical shifts were determined by analysis of 2D spectra.

b nd: not detected.

The relative configuration of the polyketide chain of **5** was assigned to be identical to that of **1**. Regarding the C-5′ configuration: **5a** (5′*S*) or **5b** (5′*R*), theoretical methods were carried out to unambiguously assign it. The 5′*R* relative configuration was suggested based on a DP4+ probability analysis of 100.00% (Table S1). Furthermore, the predicted ECD spectra for enantiomers **5** and *ent-* **5** were calculated using TDDFT, whose results were compared with the experimental ECD curve (Figure S54), establishing the absolute configuration of **5** as 5′*R*,12*S*,20*S*,21*S*,22*R*,23*R*,24*R*,25*S*,26*R*,27*S*. Thus, the structure of **5** was proposed as a new rifamycin S derivative containing an unusual 5/6/6/6 tetracyclic pyridine-fused system.

The antibacterial activity of **1**, **2**, **4**, and **5** was evaluated against *Staphylococcus aureus* and *Enterococcus* bacterial strains (Table 4). Rifamycin **5** exhibited potent antibacterial activity against *S. aureus*, with an MIC value of 0.02 μg/mL, comparable to that of the positive control rifampicin (MIC of 0.03 μg/mL). In turn, rifamycins **1**, **2**, and **4** displayed good to moderate antibacterial activity against one or both bacterial strains.

**Table 4:** Antibacterial Activity of Compounds 1, 2, 4, and 5 (MIC, μg/ml)

compound	*S. aureus* (ATCC 29,213)	*S. aureus* (ATCC 43,300, MRSA)	*E. faecalis* (ATCC 29,219)	*E. faecalis* (ATCC 51,212, VRE)	*E. coli*
**1**	3.9	2.0	15.6	31.2	^a^
**2**	62.5	31.2	15.6	125.0	^a^
**4**	62.5	62.5	^a^	^a^	^a^
**5**	0.02	0.02	0.2	3.1	100.0

a No activity was observed in the tested concentrations. Rifampicin showed an MIC ≤0.03 μg/mL.

The identification of rifamycins from *S. arenicola* has been previously reported,^20,24^ but little attention has been given to the isolation of new rifamycins. In this study, five rifamycin S derivatives, designated as salinirifamycins A–E (**1**–**5**), were isolated from *S. arenicola*. Salinirifamycins A–D (**1**–**4**) represent new rifamycin derivatives bearing an unusual 2-oxopropyl group as a substituent, while salinirifamycin E (**5**) constitutes a new and unusual 5/6/6/6 tetracyclic pyridine-rifamycin, which, in a preliminary *in vitro* study, showed potent antibacterial activity against *S. aureus*, similar to the standard rifampicin, a well-known antibiotic currently used to treat infectious diseases. The isolation of compounds **1**–**5** highlights the versatility of *Salinispora* species in producing rifamycins bearing unusual substituents, which may enhance their biological activity and increase prospects for the development of new antibacterial agents.

## Experimental Section

### General Experimental Procedures

Optical rotations were measured by using a JASCO P-2000 digital polarimeter. Ultraviolet–visible (UV–vis) spectra were acquired on a SHIMADZU 2600 UV–vis spectrophotometer. Electronic circular dichroism (ECD) measurements were carried out on a JASCO J-815 spectropolarimeter (JASCO, Japan). ECD spectra were acquired over a range of 200 to 450 nm in a quartz cuvette with a path length of 1 mm at room temperature. The spectra were recorded with a scan speed of 100 nm/min in 1.0 nm increments. The samples were dissolved in methanol, and all measurements were repeated at least 3 times. IR spectra were recorded using a PerkinElmer Spectrum 100 FTIR spectrometer equipped with a universal attenuated total reflectance accessory (UATR) in the range of 4000 to 650 cm^–1^. NMR spectra were obtained on either a Bruker Avance DRX-500 (500 MHz to ^1^H and 125 MHz to ^13^C) or an Agilent DD2- 600 spectrometer (600 MHz to ^1^H and 150 MHz to ^13^C). High-resolution electrospray ionization mass spectra (HRESIMS) were acquired using an Acquity Xevo UPLC-QTOF-MS system from Waters (Milford, MA). Sephadex LH-20 (Pharmacia) and SPE cartridges C18 (20*g*/60 mL; Strata, Phenomenex) were used for the chromatographic fractionations. HPLC analyses were carried out using a UFLC (SHIMADZU) system equipped with an SPD-M20A diode array UV–Vis detector, a Phenomenex Luna 5 μm C18 column (10.0 × 250 mm), and a Phenomenex Luna 5 μm silica column (10.0 × 250 mm).

### Bacterial Material

The bacterial strain *S. arenicola* (BRA-213) was previously identified and deposited in GenBank (accession number MH910695) by Bauermeister et al.^25^ The bacterial strain was isolated on solid A1 media [18 g of agar, 10 g of starch, 4 g of yeast extract, and 2 g of peptone dissolved in 1 L of artificial seawater, Red Sea Fish Pharm Ltd. (40.0 g/L)] from marine sediment collected at Saint Peter and Saint Paul Archipelago—SPSPA, Pernambuco, Brazil, at a depth of 16 m (N 0°55′, W 29°38′) in November 2011. A voucher strain is preserved at the Laboratório de Bioprospecção e Biotecnologia Marinha (LaBBMar), Universidade Federal do Ceará, Brazil. The license for the collection was granted by the Conselho de Gestão do Patrimônio Genético (CGEN) (SisGen No. AA8F8B8).

### Fermentation

Colonies of the strain BRA-213 were inoculated into Erlenmeyer flasks (2 L) containing 500 mL of A1 media (10 g of soluble starch, 4 g of yeast extract, and 2 g of peptone) supplemented with calcium carbonate (0.5 g/L CaCO_3_), solutions of iron­(III) sulfate (5 g/L Fe_2_(SO_4_)_3_) and potassium bromide (5 g/L KBr). Sterile Amberlite XAD-16 resin (10 g) was added to each flask on day 2. The culture flasks were shaken at 200 rpm at 28 °C for 14 days.

### Extraction and Isolation

The whole fermentation broth (20 L) was filtered through a cloth to separate the resin from the supernatant. The resin was then extracted with acetone (2 L), which was further removed under vacuum, while the residual aqueous phase was extracted with ethyl acetate (3 × 500 mL). The resulting EtOAc fraction was vacuum-dried, yielding an organic extract (9.0 g), which was fractionated on a C18 cartridge by elution with MeOH/H_2_O (70:30; 80:20) and MeOH to give three fractions (A–C). Fraction A (5.0 g) was chromatographed using a C18 cartridge and eluted with MeOH/H_2_O (30:70; 40:60; 50:50; 60:40; 70:30; 80:20) and MeOH to give seven fractions (AA–AG). Fractions AB (660 mg) and AD (910 mg) were fractionated over a Sephadex LH-20 column (50 cm × 3.5 cm id) using MeOH as eluent (flow rate of 2 drops/min) to give subfractions (AB1-AB5) and AD1–AD5, after TLC analysis, respectively. Subfraction AB3 (120.7 mg) was purified by semipreparative HPLC (MeCN/H_2_O, 50–80%, 30 min, flow rate of 3.0 mL/min) to give compounds **2** (3.0 mg, *t*_R_ = 9.2 min), **3** (0.6 mg, *t*_R_ = 10.6 min), and **4** (2.5 mg, *t*_R_ = 12.3 min). Subfraction AD2 (118.0 mg) was directly purified by semipreparative HPLC using the method MeCN/H_2_O, 50–80%, 30 min, flow rate of 3.0 mL/min, to give compound **1** (15.0 mg, *t*_R_ = 14.3 min). Fraction B (1.1 g) was fractionated over a Sephadex LH-20 column and eluted with MeOH (50 cm × 3.5 cm i.d., flow rate of 2 drops/min) to yield subfractions B1–B6. Subfraction B2 (55.0 mg) was directly purified by semipreparative HPLC (hexane/acetone, 30–100%, 20 min, flow rate of 3.0 mL/min) to give compound **5** (1.5 mg, *t*_R_ = 10.5 min).

#### Salinirifamycin A (**1**)

Yellowish solid; ${[\alpha ]}_{D}^{22}$ + 65.9 (*c* 0.13, MeOH); UV (MeOH) λ_max_ (log *ε*) 232 (3.96), 269 (3.73), 318 (3.69), 417 (3.00) nm; IR (ν_max_) 3375, 2968, 2933, 1701, 1607, 1540, 1418, 1368, 1241, 1165, 1083, 972, 814, 761 cm^–1^; ^1^H and ^13^C NMR data, see Table 1; HRESIMS *m*/*z* 768.3240 [M – H]^−^ (calcd for C_40_H_50_NO_14_, 768.3237).

#### Salinirifamycin B (**2**)

Yellowish solid; ${[\alpha ]}_{D}^{22}$ + 66.1 (*c* 0.10, MeOH); UV (MeOH) λ_max_ (log *ε*) 233 (3.87), 271 (3.65), 318 (3.60), 413 (2.88) nm; IR (ν_max_) 3342, 3242, 2976, 2884, 1670, 1606, 1542, 1427, 1358, 1262, 1190, 1076, 973, 801, 721 cm^–1^; ^1^H and ^13^C NMR data, see Table 1; HRESIMS *m*/*z* 784.3205 [M – H]^−^ (calcd for C_40_H_50_NO_15_, 784.3186).

#### Salinirifamycin C (**3**)

Yellowish solid; ${[\alpha ]}_{D}^{22}$ + 60.5 (*c* 0.10, MeOH); UV (MeOH) λ_max_ (log *ε*) 232 (3.61), 269 (3.63), 320 (3.37), 405 (2.70) nm; IR (ν_max_) 3364, 2970, 2924, 1716, 1638, 1603, 1544, 1466, 1371, 1242, 1175, 1064, 964, 808, 729 cm^–1^; ^1^H and ^13^C NMR data, see Table 2; HRESIMS *m*/*z* 754.3095 [M – H]^−^ (calcd for C_39_H_48_NO_14_, 754.3080).

#### Salinirifamycin D (**4**)

Yellowish solid; ${[\alpha ]}_{D}^{22}$ + 70.8 (*c* 0.13, MeOH); UV (MeOH) λ_max_ (log *ε*) 227 (3.60), 268 (3.43), 323 (3.12), 420 (2.34) nm; IR (ν_max_) 3350, 2924, 2852, 1707, 1674, 1608, 1543, 1433, 1348, 1236, 1163, 1070, 974, 804, 721 cm^–1^; ^1^H and ^13^C NMR data, see Table 2; HRESIMS *m*/*z* 694.2872 [M – H]^−^ (calcd for C_37_H_44_NO_12_, 694.2869).

#### Salinirifamycin E *(* **5** *)*

Red powder; ${[\alpha ]}_{D}^{22}$ + 23.1 (*c* 0.10, MeOH); UV (MeOH) λ_max_ (log *ε*) 223 (4.00), 246 (4.01), 294 (3.72), 319 (3.67), 382 (3.25), 420 (3.17), 505 (3.09) nm; IR (ν_max_) 3410, 3238, 2925, 2855, 1735, 1643, 1592, 1526, 1456, 1373, 1258, 1238, 1160, 1087, 1062, 1048, 1024, 974, 803, 723 cm^–1^; ^1^H and ^13^C NMR data, see Table 3; HRESIMS *m*/*z* 777.3597 [M + H]^+^ (calcd for C_42_H_53_N_2_O_12_, 777.3593).

### Computational NMR Chemical Shift Calculation and DP4+ Analyses

To determine the relative configuration of the rifamycin derivatives **1**–**3**, focusing on the C-4 stereocenter, the two epimeric isomers 4*R* and 4*S* were investigated: **1** (**1a** and **1b**), **2** (**2a** and **2b**), and **3** (**3a** and **3b**). For **4**, taking into account the stereocenters C-4, C-23, and C-27, four isomers were drawn: **4a** (4*R*, 23*R*, and 27*S*), **4b** (4*S*, 23*R*, and 27*S*), **4c** (4*R*, 23*S*, and 27*R*), and **4d** (4*S*, 23*S*, and 27*R*). Likewise, the relative configuration of C-5′ for rifamycin **5** was investigated. The geometries of the studied structures were optimized by using standard techniques.^26^ Optimization calculations were performed by using the Density Functional Theory (DFT)^27^ method at the mPW1PW91 functional,^28^ along with a 6–31G­(d,p) basis set implemented in the Gaussian 16 package.^22^ Vibrational modes of the optimized geometries were calculated to determine whether the resulting geometries are true minima or transition states. All optimization calculations were performed in solution by using the Polarizable Continuum Model (PCM)^22^ with the Integral Equation Formalism (IEF)^29^ using DMSO as the solvent. The NMR isotropic shielding constants were determined from the optimized geometries of isomers **1**–**5** with the mPW1PW91/6–31G­(d,p) level of theory based on the Gauge-Independent Atomic Orbitals (GIAO) proposal,^30^ implemented in Gaussian 16. The IEF-PCM solvation method was performed using DMSO as an implicit solvent to simulate the medium of chemical shifts of isomers **1**–**5**. To correlate the theoretically calculated chemical shifts with the experimental ones, the theoretical isotropic shielding constants $(\sigma_{\mathrm{calcd}}$ ) of carbon atoms were compared with the calculated isotropic shielding constants $(\sigma_{\mathrm{TMS}}$ ) using tetramethylsilane (TMS) as reference: $\delta_{C\left(\mathrm{calcd}\right)}=\sigma_{C\left(\mathrm{TMS}\right)}-\sigma_{\mathrm{calcd}}$ , where the $\sigma_{C\left(\mathrm{TMS}\right)}$ = 196.7782 ppm. To elucidate the most likely isomers of **1**–**5**, a supplemental DP4+analysis,^31^ combining quantum chemical calculations of NMR parameters with refined statistical data, was performed.

### Computational Details

For time-dependent density functional theory (TD-DFT) calculations, geometry optimizations were previously performed using DFT with the B3LYP functional and the 6-311g­(d,p) basis set. The Polarizable Continuum Model (PCM), using the integral equation formalism variant (IEFPCM), was used to describe the solvent effect.^22^ Input geometries for the new rifamycin derivatives were constructed based on relative configurations obtained from NMR data and DP4+ analyses and their ent-structures. A vibrational frequency analysis was performed on the optimized structures, and no negative frequencies were observed for the compounds studied. Based on the optimized structures, circular dichroism (CD) spectra were computed to determine the absolute configurations using time-dependent density functional theory (TD-DFT) at the M06-2X/6-31G­(d) level of theory. The CD data were extracted from the output files using GaussSum 3.0 software.^32^

### Antimicrobial Assay

The antibacterial activity of rifamycin derivatives **1**, **2**, **4**, and **5** was assayed on 4 g-positive bacteria: *Staphylococcus aureus* (ATCC 29213), methicillin-resistant *Staphylococcus aureus* (ATCC 43300, MRSA), *Enterococcus faecalis* (ATCC 29212), and vancomycin-resistant *Enterococcus faecalis* (ATCC 51212, VRE). Initially, the strains were seeded in Petri dishes containing Mueller Hinton agar (Difco), which were incubated for 24 h at a temperature of 35 ± 2 °C under aerobic conditions. Subsequently, the isolated colonies were collected and suspended in a sterile saline solution (NaCl 0.85% (*w/v*)). This suspension was then used to obtain the bacterial inoculum in Mueller Hinton Broth -MHB (Difco) with a final bacterial concentration of 5 × 10^5^ CFU/mL. The method used was broth microdilution in a 96-well plate, and the minimum inhibitory concentrations (MICs) of the substances were determined according to the Clinical Laboratory Standards Institute (2015).^33^ Rifampicin was used as a positive control, and the microplates were incubated under the same conditions as those mentioned above. The MIC value was determined after observing the absence of bacterial growth in the culture medium.

## Supplementary Material





## Data Availability

The NMR data for salinirifamycin A, C, and E (**1**, **3**, and **5**) have been deposited at the Natural Products Magnetic Resonance Database (NP-MRD; https://np-mrd.org/) and can be found at NP-MRD ID NP0351016 for salinirifamycin A (**1**), NP0351017 for salinirifamycin C (**3**), and NP0351018 for salinirifamycin E (**5**).

## Notes

Raw NMR data for **2** (ZIP) Raw NMR data for **4** (ZIP)
HRESIMS, NMR data, and ECD data of **1**–**5** (PDF)

## References

[1] Subramami R., Sipkema D. (2019). Marine rare Actinomycetes: A promising source of structurally
diverse and unique novel natural products. Mar.
Drugs.

[2] Manivasagan P., Kang K. H., Sivakumar K., Li-Chan E. C. Y., Oh H. M., Kim S. K. (2014). Marine Actinobactéria: An important source of
bioactive natural products. Environ. Toxicol.
Pharacol..

[3] Jensen P. R., Moore B. S., Fenical W. (2015). The marine actinomycete genus *Salinispora*: a model organism for secondary metabolite discovery. Nat. Prod. Rep..

[4] Maldonado L. A., Fenical W., Jensen P. R., Kauffman C. A., Mincer T. J., Ward A. C., Bull A. T., Goodfellow M. (2005). *Salinispora
arenicola* gen. nov., sp. nov. and *Salinispora tropica* sp. nov., obligate marine actinomycetes belonging to the family
Micromonosporaceae. Int. J. Syst. Evol. Microbiol..

[5] Román-Ponce B., Millán-Aguiñaga N., Guillen-Matus D., Chase A. B., Ginigini J. G. M., Soapi K., Feussner K. D., Jensen P. R., Trujillo M. E. (2020). Six novel species
of the obligate
marine actinobacterium *Salinispora*, *Salinispora
cortesiana* sp. nov., *Salinispora fenicalii* sp. nov., *Salinispora goodfellowii* sp. nov., *Salinispora mooreana* sp. nov., *Salinispora oceanensis* sp. nov. and *Salinispora vitiensis* sp. nov., and
emended description of the genus *Salinispora*. Int. J. Syst. Evol. Microbiol..

[6] Feling R. H., Buchanan G. O., Mincer T. J., Kauffman C. A., Jensen P. R., Fenical W. (2003). Salinosporamide A:
a highly cytotoxic proteasome inhibitor
from a novel microbial source, a marine bacterium of the new genus *Salinospora*. Angew. Chem., Int. Ed..

[7] Murphy B. T., Narender T., Kauffman C. A., Woolery M., Jensen P. R., Fenical W. (2010). Saliniquinones A-F
new members of the highly cytotoxic
anthraquinone-γ-pyrones from the marine actinomycete *Salinispora arenicola*. Aust. J. Chem..

[8] Williams P. G., Miller E. D., Asolkar R. N., Jensen P. R., Fenical W. (2007). Arenicolides
A-C 26-Membered ring macrolides from the marine actinomycete *Salinispora arenicola*. J. Org. Chem..

[9] Williams P. G., Asolkar R. N., Kondratyuk T., Pezzuto J. M., Jensen P. R., Fenical W. (2007). Saliniketals A and
B, bicyclic polyketides from the
marine actinomycete *Salinispora arenicola*. J. Nat. Prod..

[10] Asolkar R. N., Freel K. C., Jensen P. R., Fenical W., Kondratyuk P., Park E. J., Pezzuto J. M. (2009). Arenamides A-C, cytotoxic NFkappaB
inhibitors from the marine actinomycete *Salinispora arenicola*. J. Nat. Prod..

[11] Duncan K. R., Crüsemann M., Lechner A., Sarkar A., Li J., Ziemert N., Wang M., Bandeira N., Moore B. S., Dorrestein P. C., Jensen P. R. (2015). Molecular networking and pattern-based
genome mining improves discovery of biosynthetic gene clusters and
their products from *Salinispora* species. Chem. Biol..

[12] Sensi P. (1957). Applications
of paper chromatography & countercurrent distribution to steroids
& antibiotics. Boll. Chim. Farm..

[13] Aristoff P.
A., Garcia G. A., Kirchhoff P. D., Showalter H. D. H. (2010). Rifamycins
– Obstacles and opportunities. Tuberculosis.

[14] Ramos-E-Silva M., Rebello P. F. B. L. (2001). Recognition
and treatment. Am.
J. Clin. Dermatol..

[15] Sepkowitz K. A., Raffalli J., Riley L., Kiehn T. E., Armstrong D. (1995). Tuberculosis
in the AIDS era. Clin. Microbiol. Rev..

[16] Pinto F. C. L., Silveira E. R., Vasconcelos A. C. L., Florêncio K. G. D., Oliveira F. A. S., Sahm B. B., Costa-Lotufo L. V., Bauermeister A., Lopes N. P., Wilke D. V., Pessoa O. D. L. (2020). Dextrorotatory
Chromomycins from the Marine *Streptomyces* sp. Associated
to *Palythoa caribaeorum*. J.
Braz. Chem. Soc..

[17] Sousa T. D. S., Jimenez P. C., Ferreira E. G., Silveira E. R., Braz-Filho R., Pessoa O. D. L., Costa-Lotufo L. V. (2012). Anthracyclinones
from *Micromonospora* sp. J.
Nat. Prod..

[18] Chen M., Roush W. R. (2013). Crotylboron-Based
synthesis of the polypropionate units
of Chaxamycins A/D, Salinisporamycin, and Rifamycin S. J. Org. Chem..

[19] August P. R., Tang L., Yoon Y. J., Ning S., Muller R., Yu T. W., Taylor M., Hoffmann D., Kim C. G., Zhang X., Hutchinson C. R., Floss H. G. (1998). Biosynthesis of
the ansamycin antibiotic rifamycin: deductions from the molecular
analysis of the rif biosynthetic gene cluster of *Amycolatopsis
mediterranei* S699. Chem. Biol..

[20] Kim H., Kim S., Kim M., Lee C., Yang I., Nam S. J. (2020). Bioactive
natural products from the genus *Salinospora*: a review. Arch. Pharm. Res..

[21] Silverstein, R. M. ; Webster, F. X. Spectrometric Identification of Organic Compounds, 7th ed.; John Wiley & Sons, Inc: New York, 2005.

[22] Frisch, M. J. ; Trucks, G. W. ; Schlegel, H. B. ; Scuseria, G. E. ; Robb, M. A. ; Cheeseman, J. R. ; Scalmani, G. ; Barone, V. ; Petersson, G. A. ; Nakatsuji, H. , Gaussian 16, Revision C.01; Gaussian, Inc.: Wallingford CT, 2016.

[23] Tomasi J., Mennucci B., Cammi R. (2005). Quantum mechanical
continuum solvationmodels. Chem. Rev..

[24] Wilson M. C., Gulder T. A. M., Mahmud T., Moore B. S. (2010). Shared biosynthesis
of the Saliniketals and Rifamycins in *Salinispora arenicola* is controlled by the sare1259-Encoded Cytochrome P450. J. Am. Chem. Soc..

[25] Bauermeister A., Velasco-Alzate K., Dias T., Macedo H., Ferreira E. G., Jimenez P. C., Lotufo T. M. C., Lopes N. P., Gaudêncio S. P., Costa-Lotufo L. V. (2018). Metabolomic fingerprinting of *Salinispora* from atlantic oceanic islands. Front. Microbiol.

[26] Fletcher, R. Practical methods of optimization, 2nd ed.; Wiley: New York, 1981.

[27] Zinola, C. F. Electrocatalysis: computational, Experimental, and Industrial Aspects, 1st ed.; CRC Press: Boca Raton, 2010.

[28] Perdew J. P., Burke K., Ernzerhof M. (1996). Generalized gradient approximation
made simple. Phys. Rev. Lett..

[29] Mennucci B. (2012). Polarizable
continuum model. WIREs Comput. Mol. Sci..

[30] Wolinski K., Hinton J. F., Pulay P. (1990). Efficient
implementation of the Gauge-Independent
Atomic Orbital method for NMR chemical shift calculations. J. Am. Chem. Soc..

[31] Smith S. G., Goodman J. M. (2010). Assigning stereochemistry to single diastereoisomers
by GIAO NMR calculation: The DP4 Probability. J. Am. Chem. Soc..

[32] O’Boyle N. M., Tenderholt A. L., Langner K. M. (2008). cclib: A library for package-independent
computational chemistry algorithms. J. Comput.
Chem..

[33] CLSI. Clinical Laboratory Standards Institute Approved standard M07-A10; CLSI: Wayne, PA, 2014.

