# Supplementary material for: Salinirifamycins A–E: Rifamycin S Derivatives from the Brazilian Marine Actinomycete Salinispora arenicola
Source: J Nat Prod. 2026 Jan 1;89(1):304–12. doi: 10.1021/acs.jnatprod.5c01381 (PMC12836363; doi:10.1021/acs.jnatprod.5c01381)
Supplement: Supplementary file 2 [file np5c01381_si_002.pdf]

SUPPORTING INFORMATION

Salinirifamycins A-E: Rifamycin S Derivatives from the Brazilian Marine Actinomycete *Salinispora arenicola*

Alison B. da Silva,[†] Francisco Chagas L. Pinto,[†] Edilberto R. Silveira,[†] Tercio de Freitas Paulo,[†] Diego V. Wilke,[‡] Elthon G. Ferreira,[‡] Leticia V. Costa-Lotufo,[§] Kirley M. Canuto,[⊥] José Delano Barreto Marinho Filho,[ǁ] Ayslan B. Barros,[ǁ] Genoveffa Nuzzo,[∇] Angelo Fontana,[∇] Norberto Kássio. V. Monteiro,[#] and Otilia D. L. Pessoa*[,†]

[†]*Departamento de Química Orgânica e Inorgânica, Universidade Federal do Ceará, 60.021-970, Fortaleza-CE, Brazil*

[‡]*Núcleo de Pesquisa e Desenvolvimento de Medicamentos, Universidade Federal do Ceará, 60.430-275, Fortaleza-CE, Brazil*

[§]*Departamento de Farmacologia, Universidade de São Paulo, 05508-900, São Paulo-SP, Brazil*

[⊥]*Embrapa Agroindústria Tropical, 60.511-110, Fortaleza-CE, Brazil*

[ǁ] *Núcleo de Pesquisa e Pós-graduação, Universidade Federal do Delta do Parnaíba, 64202-020, Parnaíba-PI, Brazil*

[∇]*CNR, Istituto di Chimica Biomolecolare, Bio-Organic Chemistry Unit, 80078, Pozzuoli, Naples, Italy*

[#]*Departamento de Química Analítica e Físico-Química, Universidade Federal do Ceará, 60020-181, Fortaleza-CE, Brazil*

*Phone: +55-85-33669441. E-mail: opessoa@ufc.br

Table of Contents

Figure S1. ^{1}H NMR (600 MHz) spectrum of **1** in DMSO-d_6
Figure S2. Expanded ^{1}H NMR (600 MHz) spectrum of **1** in DMSO-d_6
Figure S3. Expanded ^{1}H NMR (600 MHz) spectrum of **1** in DMSO-d_6
Figure S4. ^{13}C-APT NMR (150 MHz) spectrum of **1** in DMSO-d_6
Figure S5. COSY NMR spectrum of **1** in DMSO-d_6
Figure S6. Edited HSQC NMR spectrum of **1** in DMSO-d_6
Figure S7. HMBC NMR spectrum of **1** in DMSO-d_6
Figure S8. Expanded HMBC NMR spectrum of **1** in DMSO-d_6
Figure S9. ROESY NMR spectrum of **1** in DMSO-d_6
Figure S10. HRESIMS spectrum of **1**
Figure S11. ^{1}H NMR (600 MHz) spectrum of **2** in DMSO-d_6
Figure S12. Expanded ^{1}H NMR (600 MHz) spectrum of **2** in DMSO-d_6
Figure S13. Expanded ^{1}H NMR (600 MHz) spectrum of **2** in DMSO-d_6
Figure S14. ^{13}C NMR (150 MHz) spectrum of **2** in DMSO-d_6
Figure S15. COSY NMR spectrum of **2** in DMSO-d_6
Figure S16. Edited HSQC NMR spectrum of **2** in DMSO-d_6
Figure S17. HMBC NMR spectrum of **2** in DMSO-d_6
Figure S18. Expanded HMBC NMR spectrum of **2** in DMSO-d_6
Figure S19. NOESY NMR spectrum of **2** in DMSO-d_6
Figure S20. HRESIMS spectrum of **2**
Figure S21. ^{1}H NMR (500 MHz) spectrum of **3** in DMSO-d_6
Figure S22. Expanded ^{1}H NMR (500 MHz) spectrum of **3** in DMSO-d_6
Figure S23. Expanded ^{1}H NMR (500 MHz) spectrum of **3** in DMSO-d_6
Figure S24. COSY NMR spectrum of **3** in DMSO-d_6
Figure S25. HSQC NMR spectrum of **3** in DMSO-d_6
Figure S26. HSQC NMR spectrum of **3** in DMSO-d_6
Figure S27. HMBC NMR spectrum of **3** in DMSO-d_6
Figure S28. Expanded HMBC NMR spectrum of **3** in DMSO-d_6
Figure S29. NOESY NMR spectrum of **3** in DMSO-d_6
Figure S30. HRESIMS spectrum of **3**
Figure S31. ^{1}H NMR (600 MHz) spectrum of **4** in DMSO-d_6
Figure S32. Expanded ^{1}H NMR (600 MHz) spectrum of **4** in DMSO-d_6
Figure S33. Expanded ^{1}H NMR (600 MHz) spectrum of **4** in DMSO-d_6
Figure S34. COSY NMR spectrum of **4** in DMSO-d_6
Figure S35. Edited HSQC NMR spectrum of **4** in DMSO-d_6
Figure S36. HMBC NMR spectrum of **4** in DMSO-d_6
Figure S37. Expanded HMBC NMR spectrum of **4** in DMSO-d_6
Figure S38. NOESY NMR spectrum of **4** in DMSO-d_6

Figure S39. ^{1}H NMR (500 MHz) spectrum of **5** in DMSO-d_6.

Figure S40. Expanded ^{1}H NMR (500 MHz) spectrum of **5** in DMSO-d_6.

Figure S41. Expanded ^{1}H NMR (500 MHz) spectrum of **5** in DMSO-d_6.

Figure S42. COSY NMR spectrum of **5** in DMSO-d_6.

Figure S43. TOCSY NMR spectrum of **5** in DMSO-d_6.

Figure S44. HSQC NMR spectrum of **5** in DMSO-d_6.

Figure S45. HMBC NMR spectrum of **5** in DMSO-d_6.

Figure S46. Expanded HMBC NMR spectrum of **5** in DMSO-d_6.

Figure S47. NOESY NMR spectrum of **5** in DMSO-d_6.
Figure S48. HRESIMS spectrum of **5**.

Table S1. Table S1. Calculated ^{13}C nuclear magnetic shielding (δ_C) using GIAO method with mPW1PW91/6–31 G(d,p) level of theory and ^{13}C NMR experimental data ($\delta_{C\ exp.}$), Root Mean Square Error (RMSE), Mean Absolute Error (MAE) and DP4+ probability values (%) for **1-5**.

Figure S49. The correlation between the experimental chemical shift ($\delta_{C\ exp.}$) versus the calculated magnetic isotropic shielding using GIAO method with *mPW1pw91/6-311G(d,p)* level of theory for (a) **1a** (4R) and (b) **1b** (4S).

Figure S50. The correlation between the experimental chemical shift ($\delta_{C\ exp.}$) versus the calculated magnetic isotropic shielding using GIAO method with *mPW1pw91/6-311G(d,p)* level of theory for (a) **2a** (4R) and (b) **2b** (4S).

Figure S51. The correlation between the experimental chemical shift ($\delta_{C\ exp.}$) versus the calculated magnetic isotropic shielding using GIAO method with *mPW1pw91/6-311G(d,p)* level of theory for (a) **3a** (4R) and (b) **3b** (4S).

Figure S52. The correlation between the experimental chemical shift ($\delta_{C\ exp.}$) versus the calculated magnetic isotropic shielding using GIAO method with *mPW1pw91/6-311G(d,p)* level of theory for (a) **4a** (4R, 23R,27S), (b) **4b** (4S, 23R,27S), (c) **4c** (4R, 23S,27R), and (d) **4c** (4S, 23R,27S).

Figure S53. The correlation between the experimental chemical shift ($\delta_{C\ exp.}$) versus the calculated magnetic isotropic shielding using GIAO method with *mPW1pw91/6-311G(d,p)* level of theory for (a) **5a** (5'R) and (b) **5b** (4S).

Figure S54. Experimental and calculated ECD spectra of **1-5** in methanol.

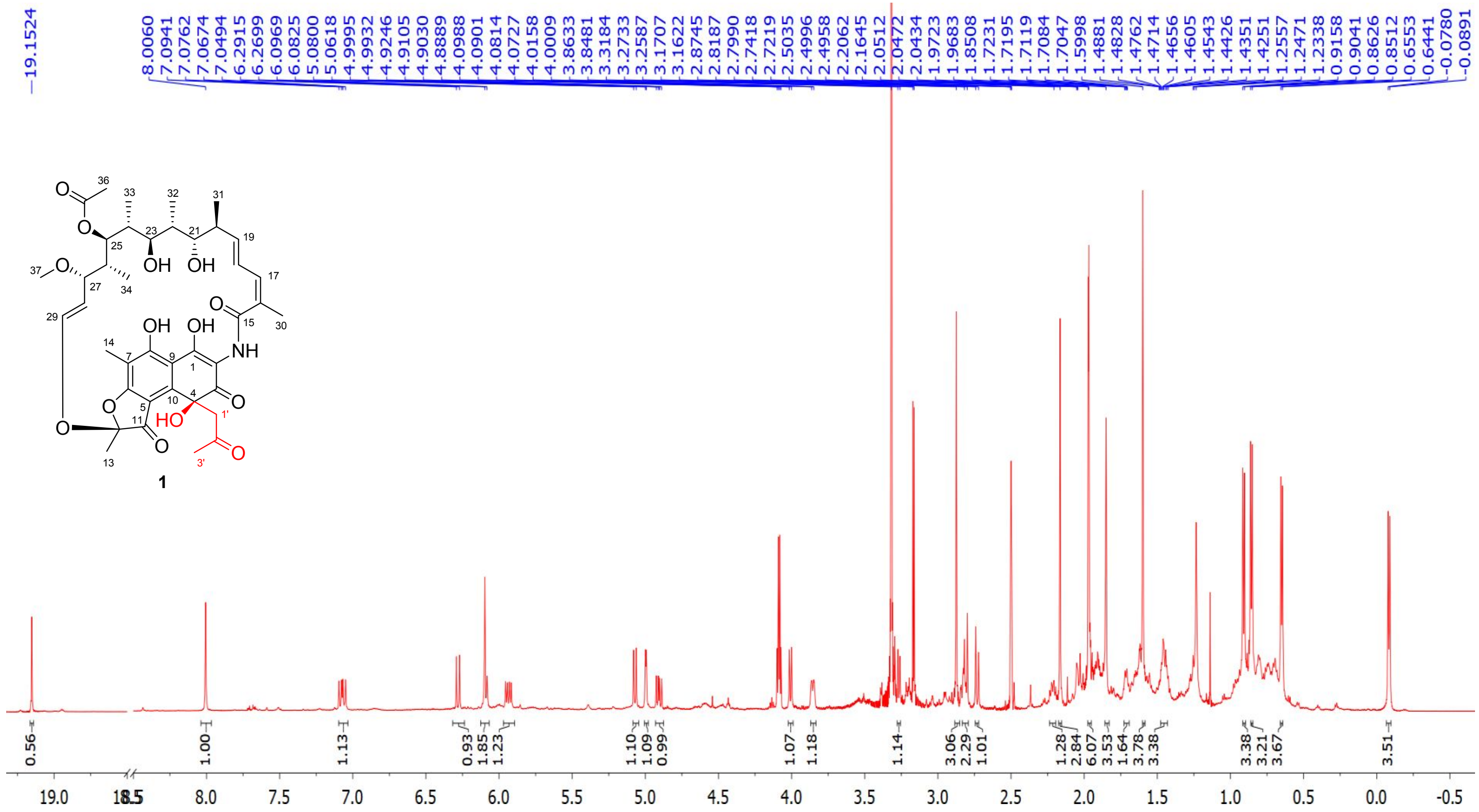

Figure S1. ^{1}H NMR (600 MHz) spectrum of **1** in DMSO-d_6.

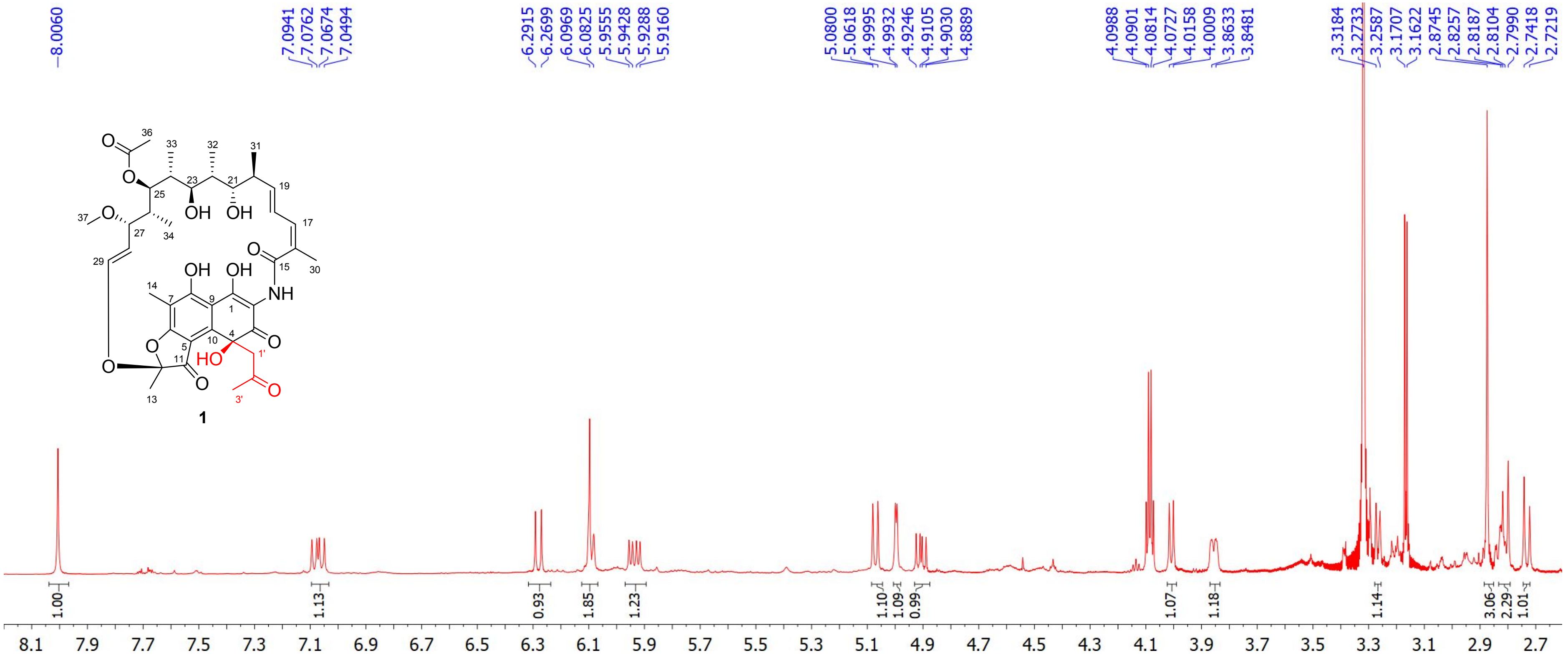

Figure S2. Expanded ^{1}H NMR (600 MHz) spectrum of **1** in DMSO-d_6.

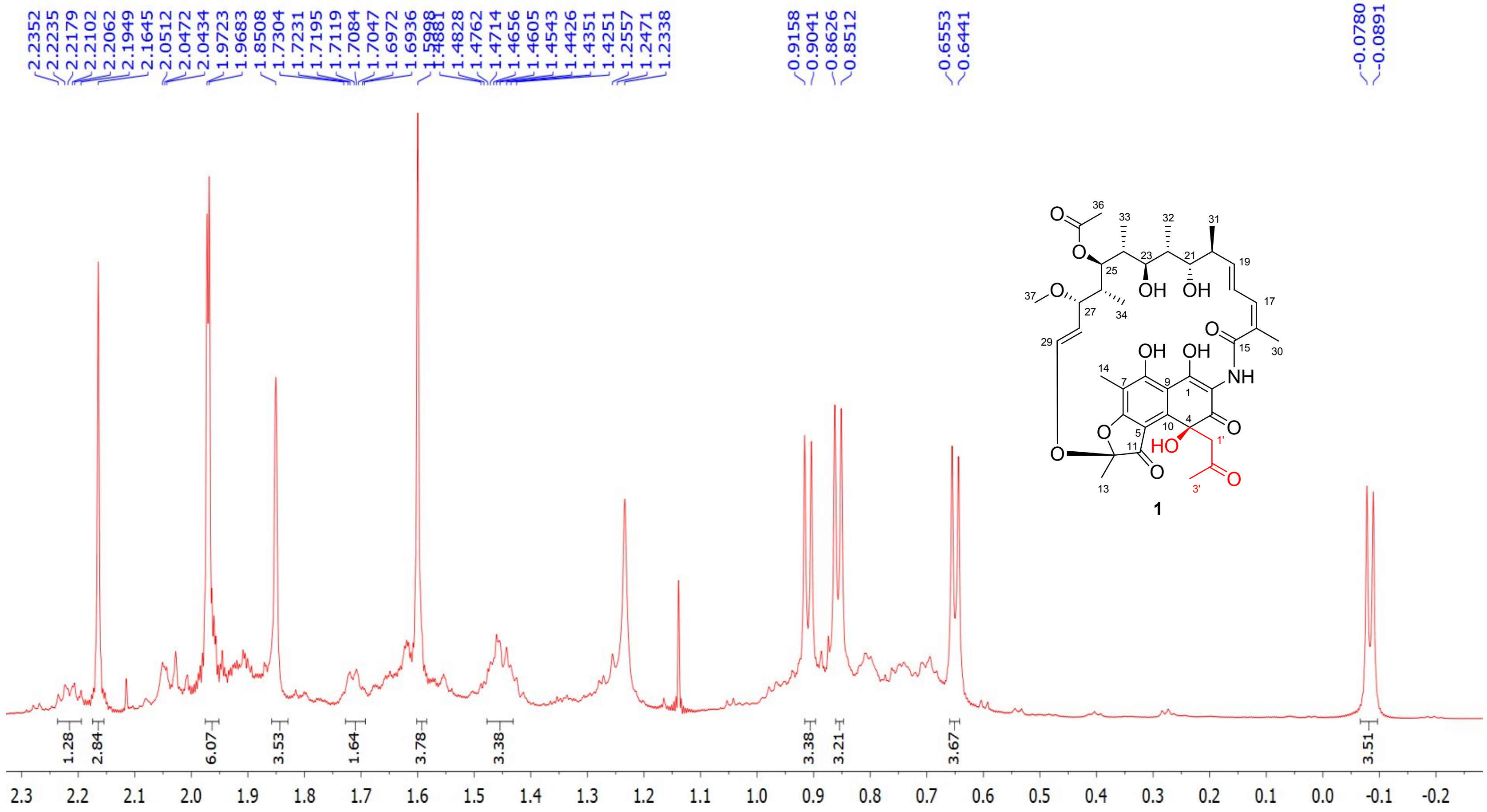

Figure S3. Expanded ^{1}H NMR (600 MHz) spectrum of **1** in DMSO-d_6.

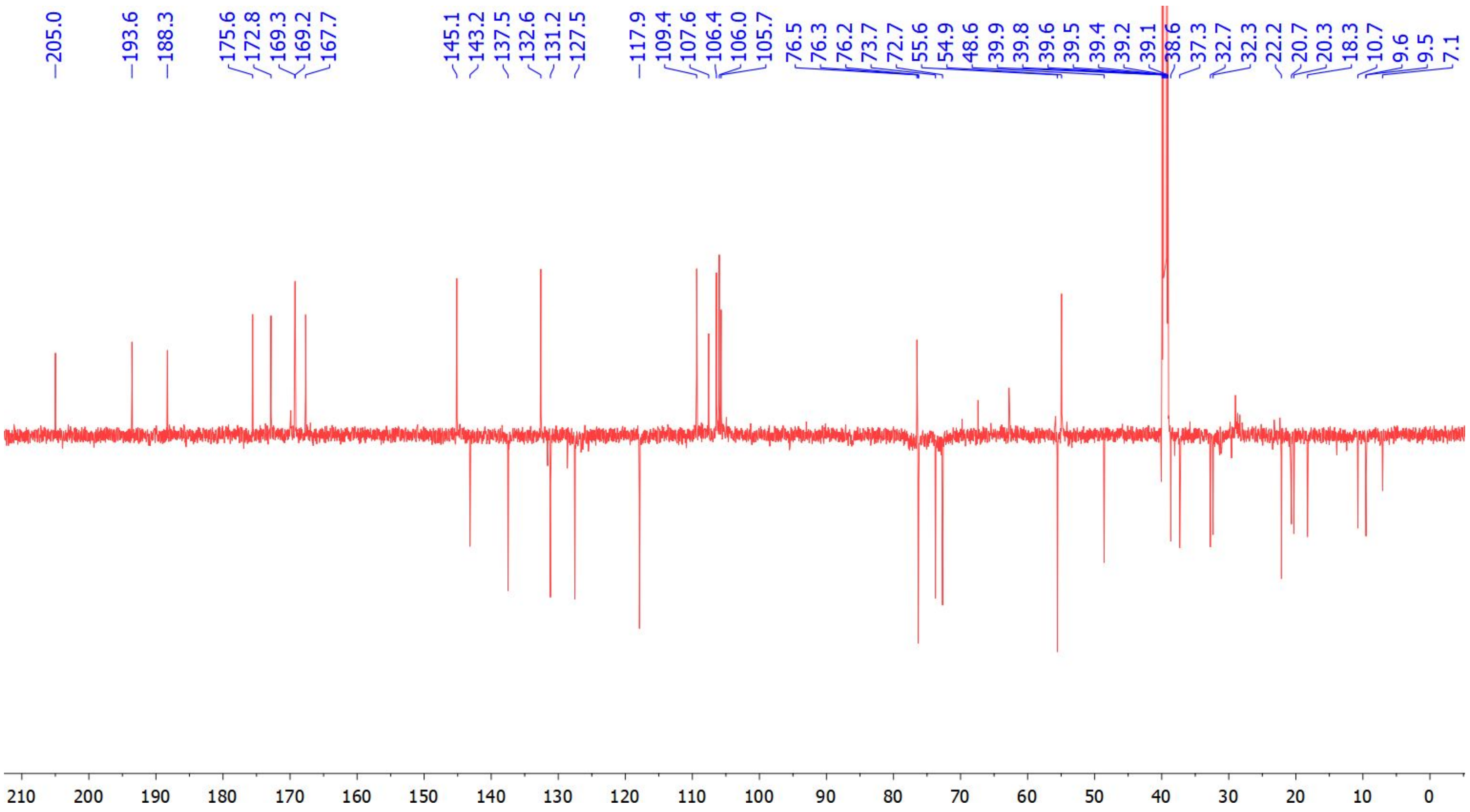

Figure S4. ^{13}C-APT NMR (150 MHz) spectrum of **1** in DMSO-d_6.

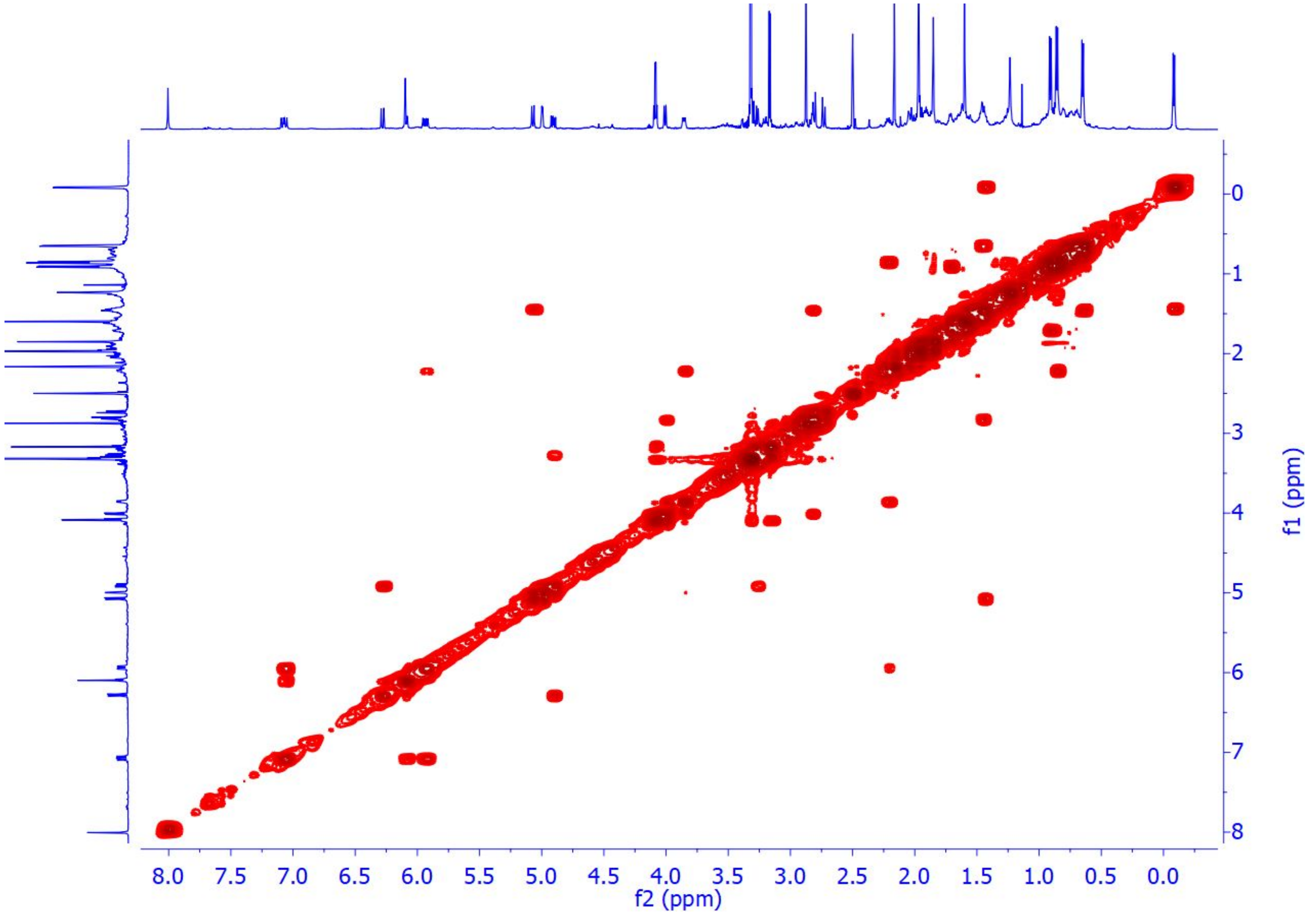

Figure S5. COSY NMR spectrum of **1** in DMSO-d_6.

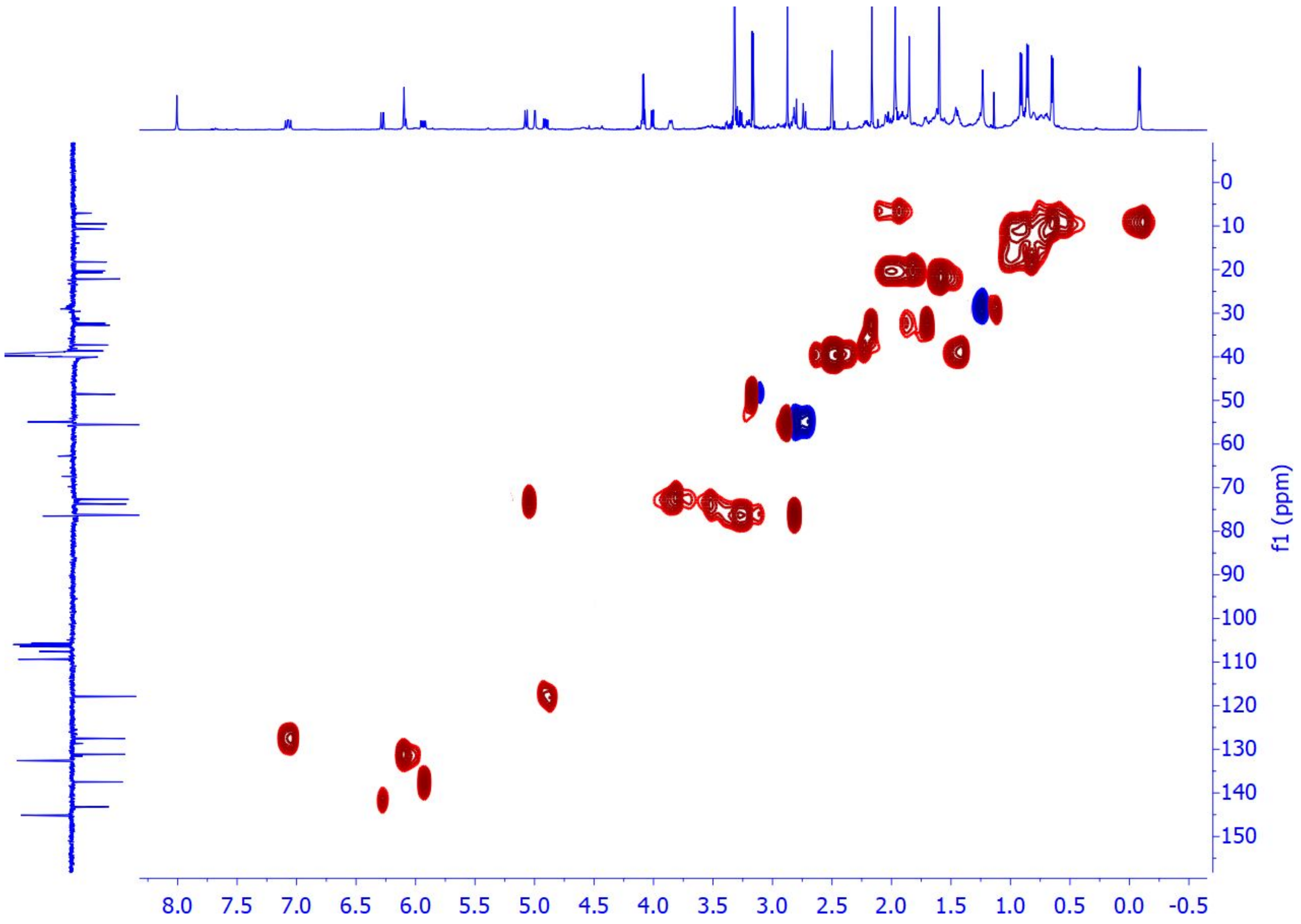

Figure S6. Edited HSQC NMR spectrum of **1** in DMSO-d_6.

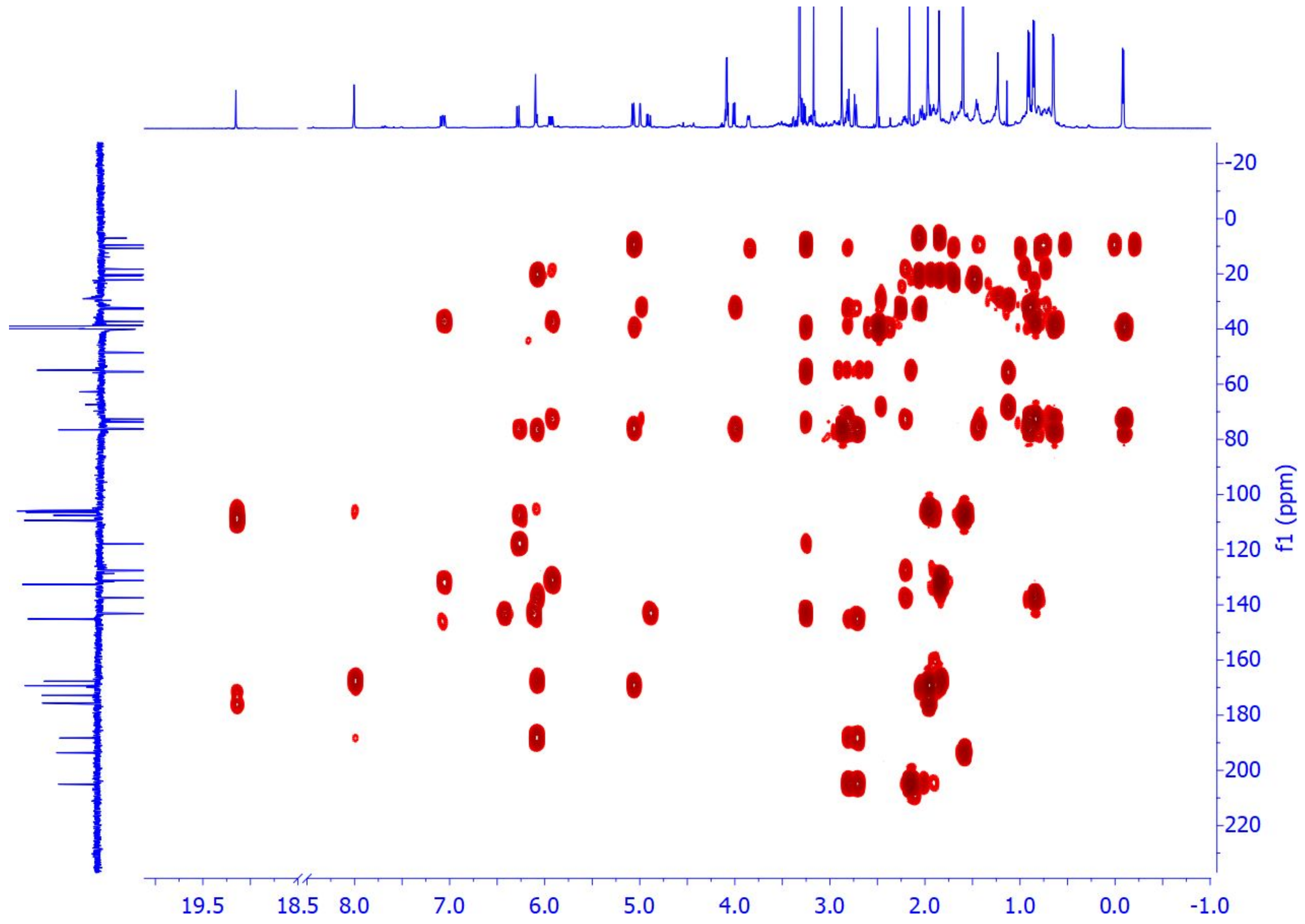

Figure S7. HMBC NMR spectrum of **1** in DMSO-d_6.

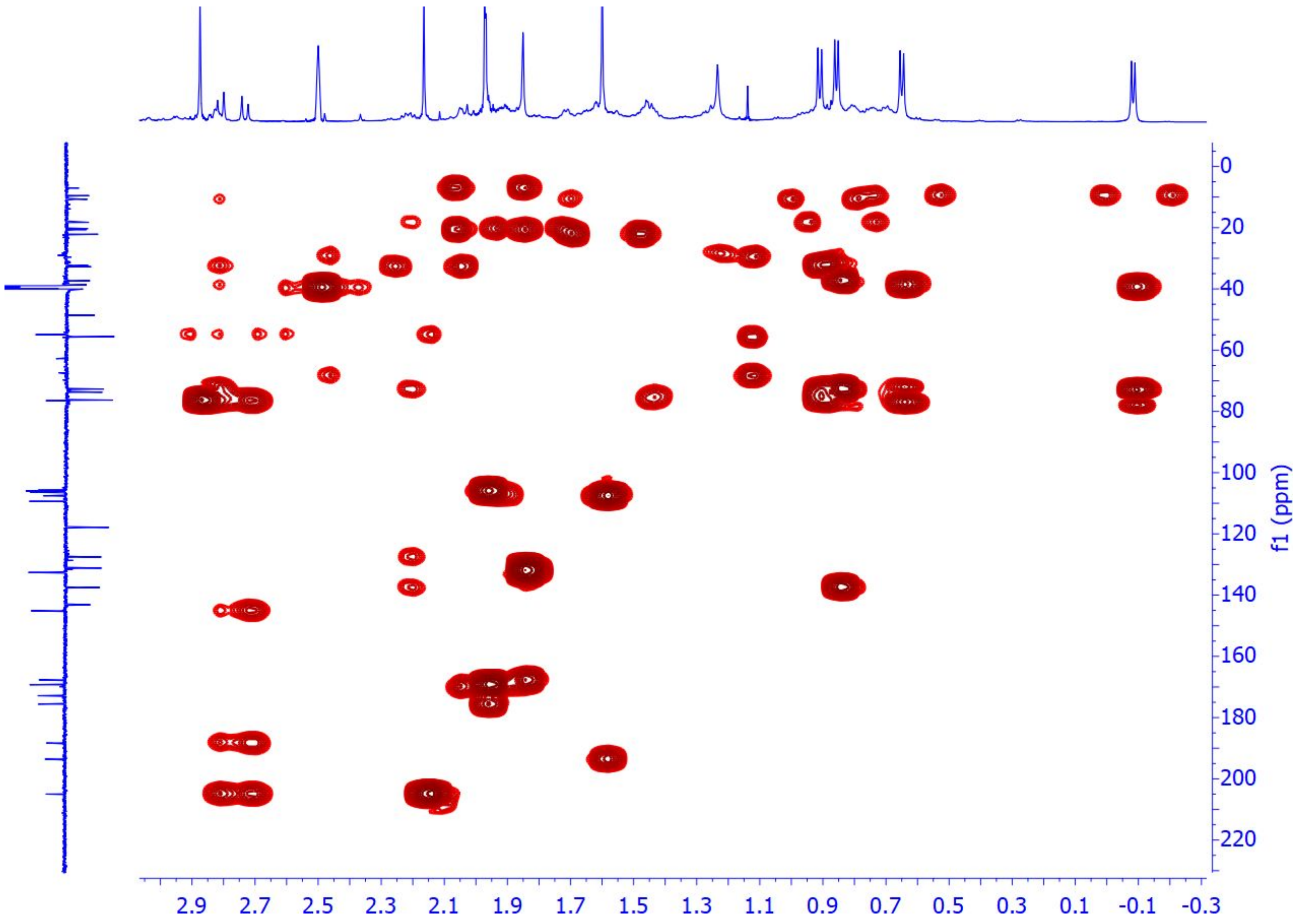

Figure S8. Expanded HMBC NMR spectrum of **1** in DMSO-d_6.

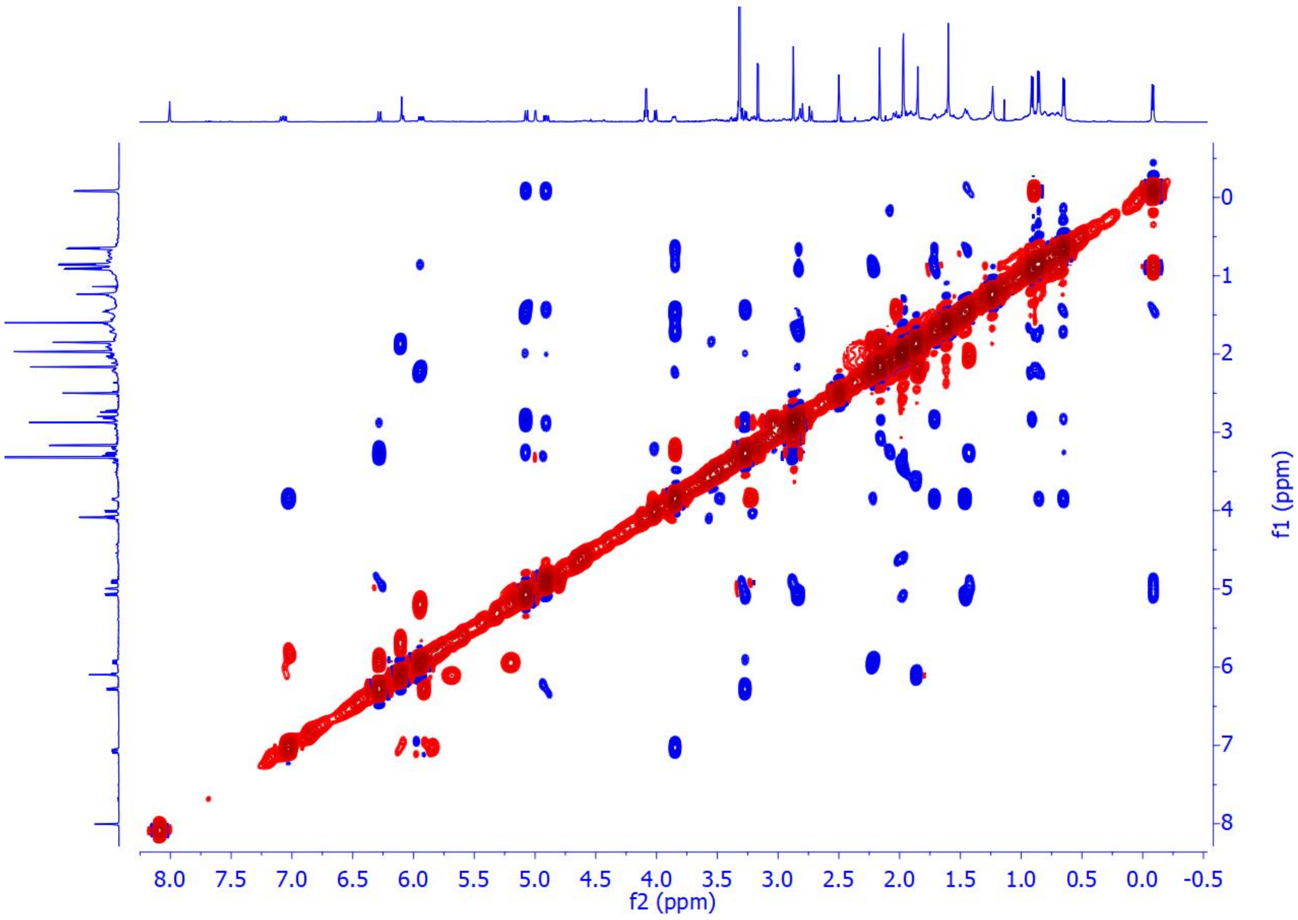

Figure S9. ROESY NMR spectrum of **1** in DMSO-d_6.

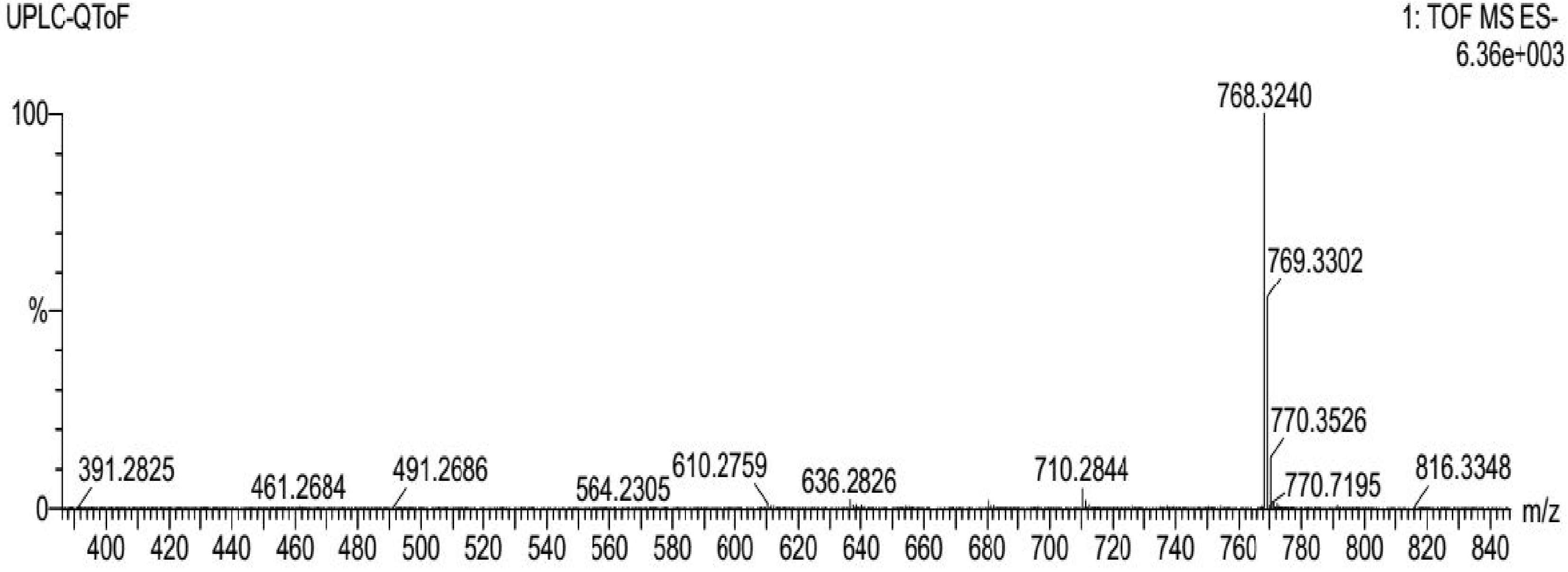

Figure S10. HRESIMS spectrum of **1**.

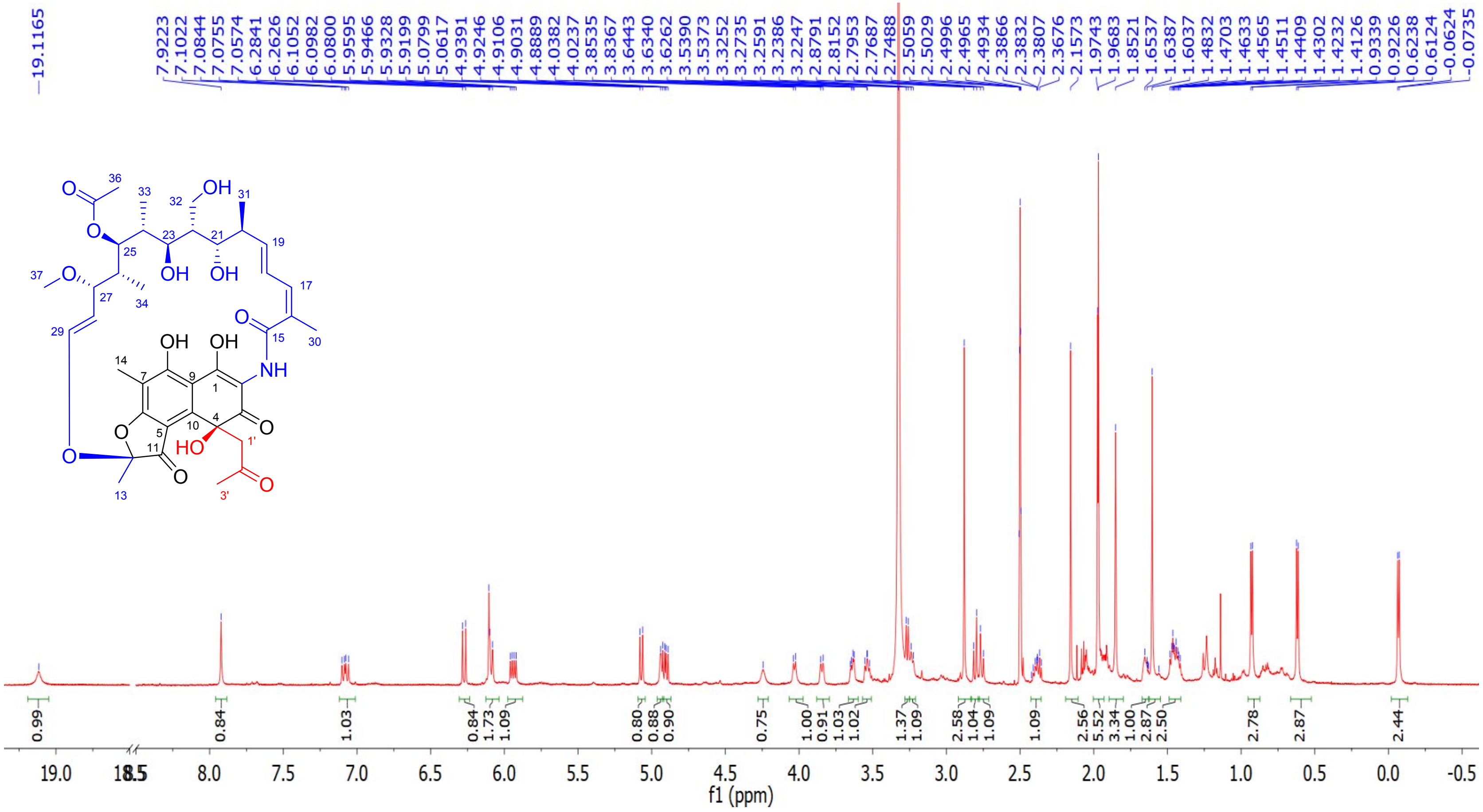

Figure S11. ^{1}H NMR (600 MHz) spectrum of **2** in DMSO-d_6.

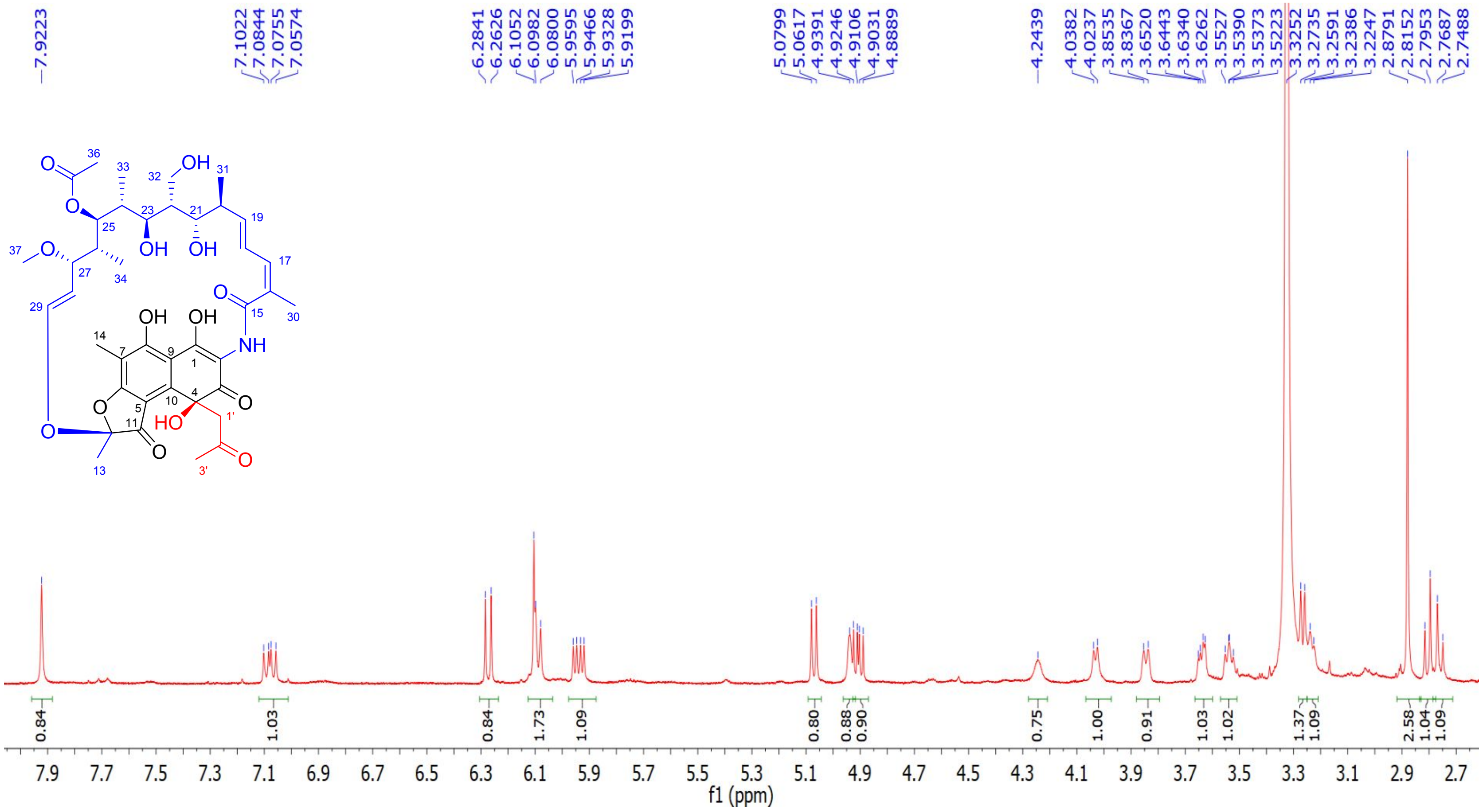

Figure S12. Expanded ^{1}H NMR (600 MHz) spectrum of **2** in DMSO-d_6.

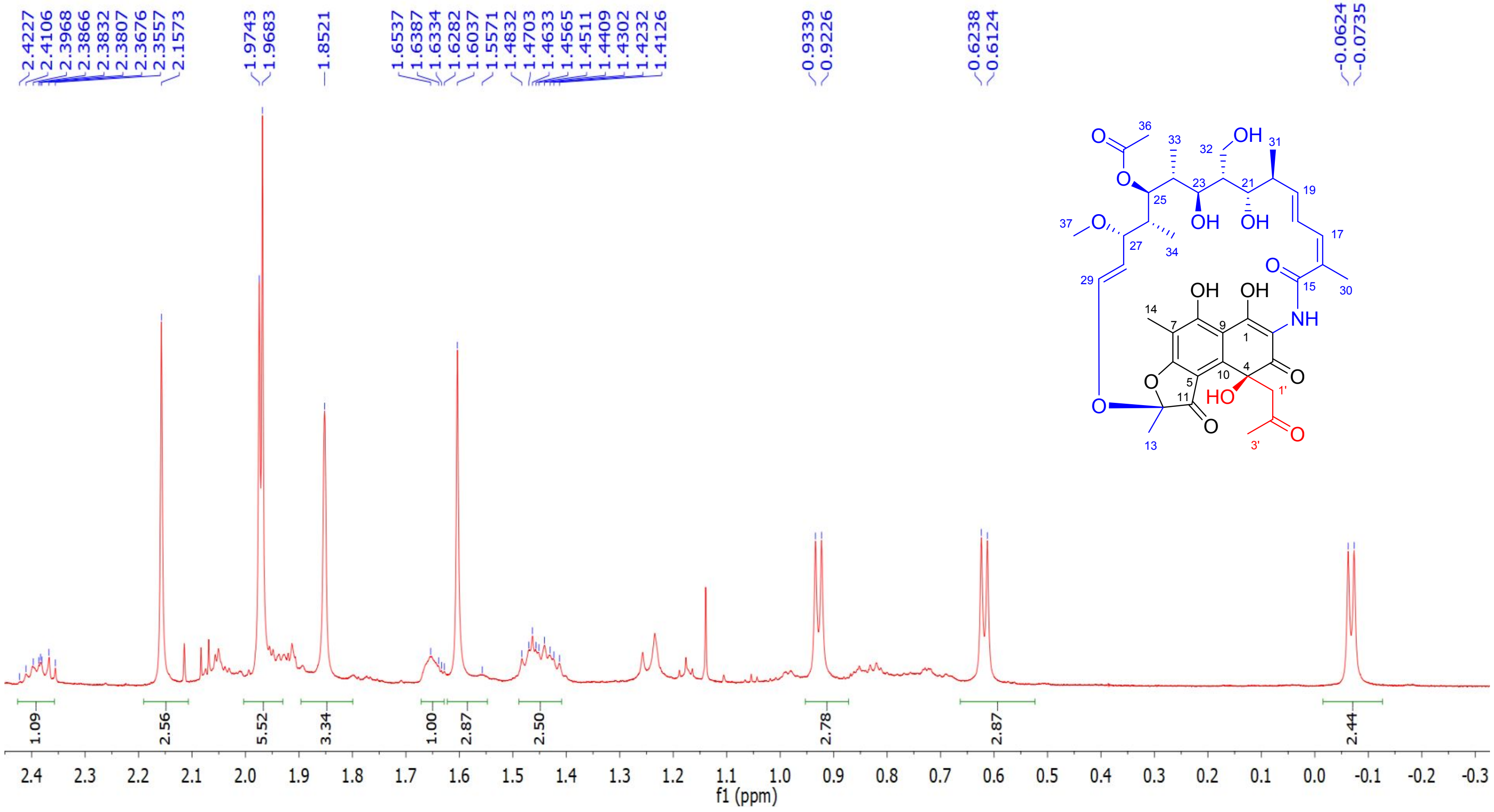

Figure S13. Expanded ^1H NMR (600 MHz) spectrum of **2** in DMSO-d_6.

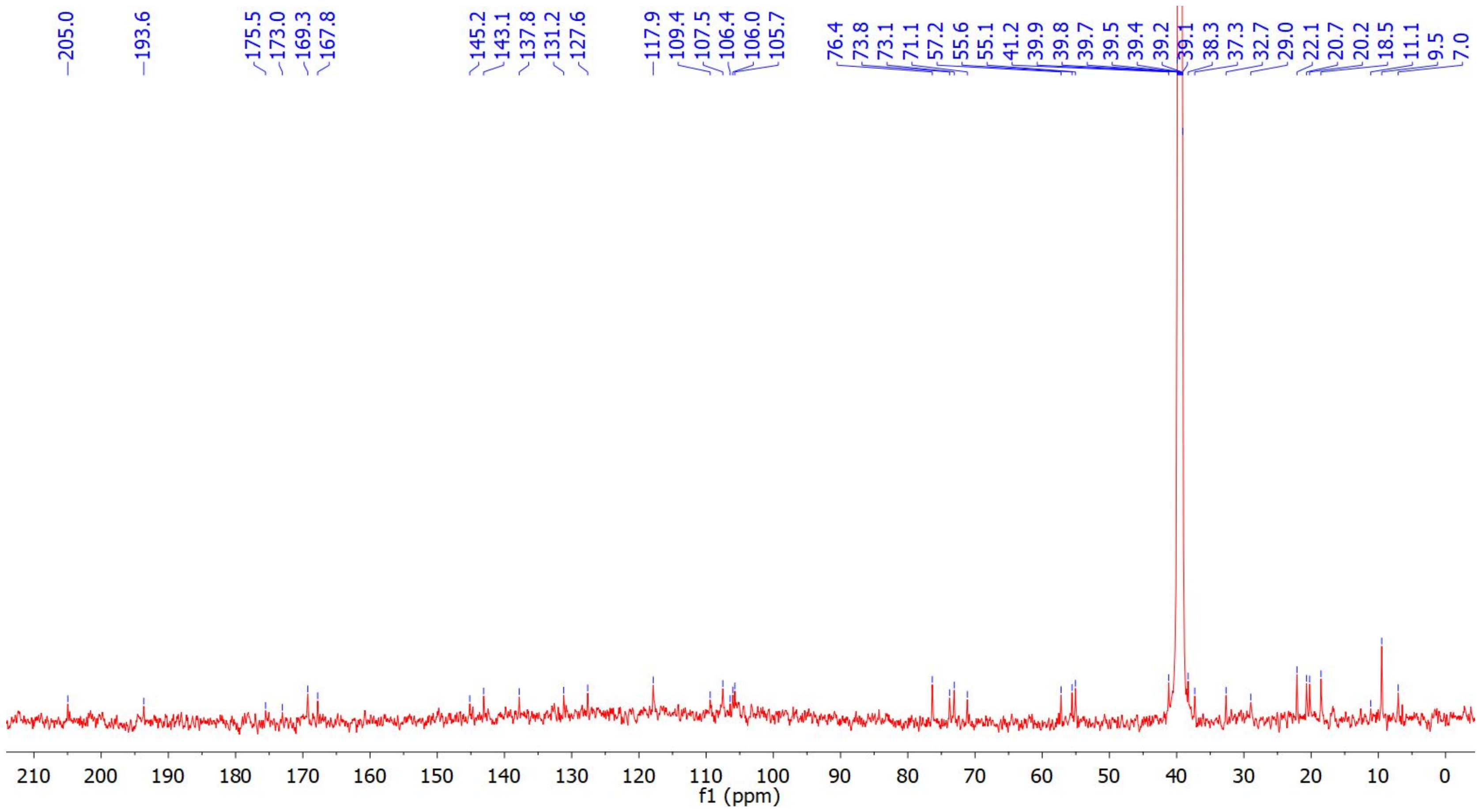

Figure S14. ^{13}C NMR (150 MHz) spectrum of **2** in DMSO-d_6.

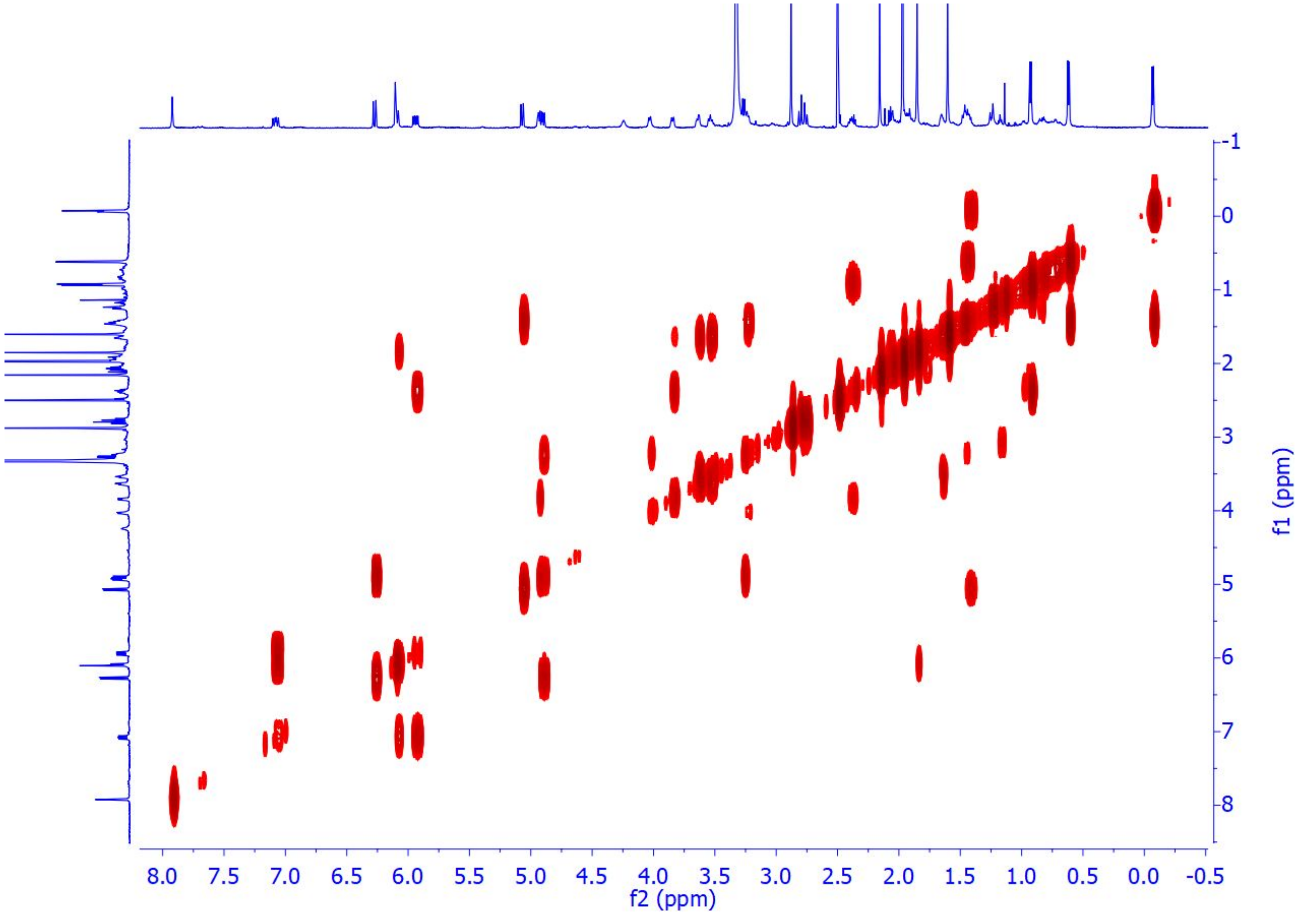

Figure S15. COSY NMR spectrum of **2** in DMSO-d_6.

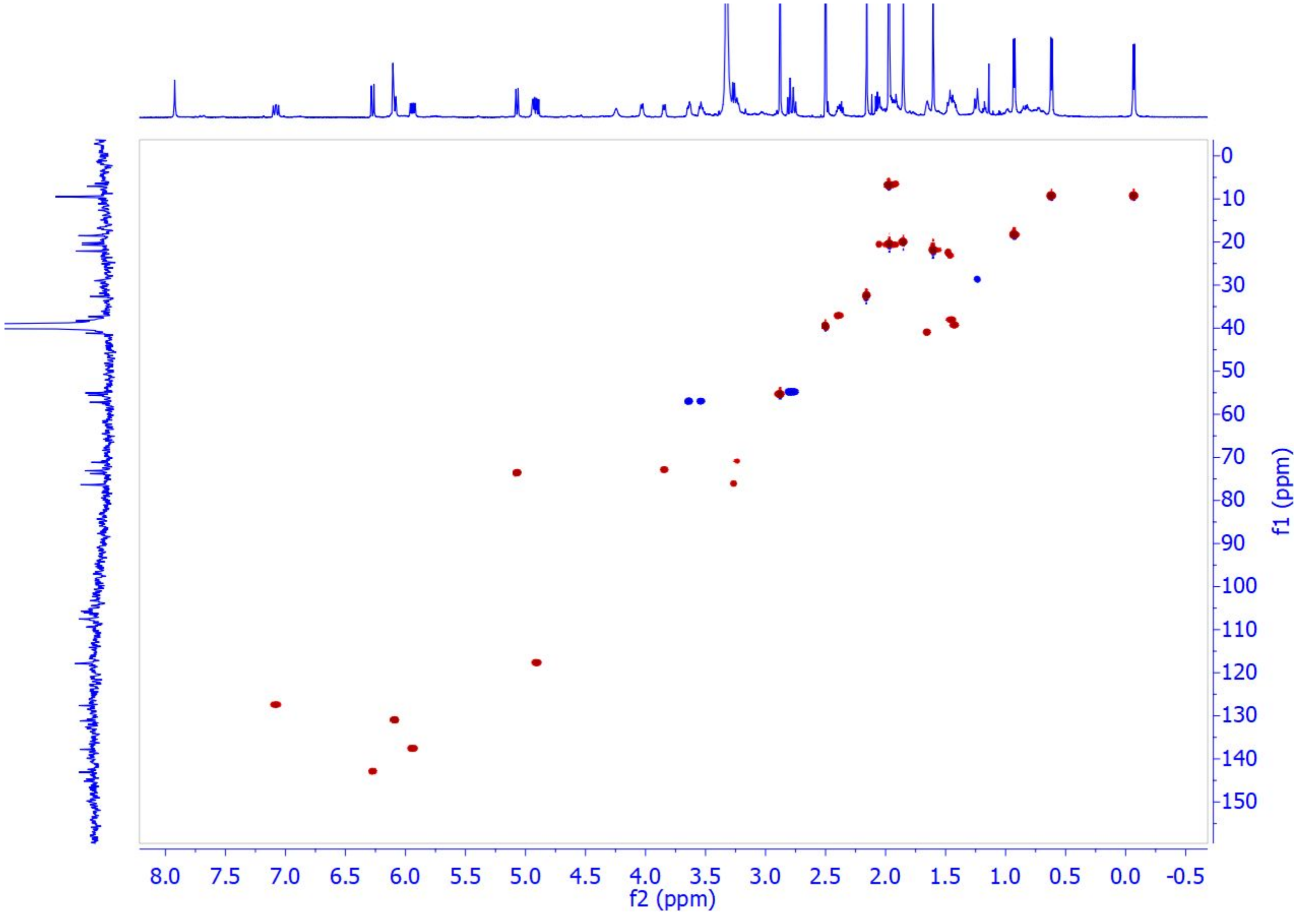

Figure S16. Edited HSQC NMR spectrum of **2** in DMSO-d_6.

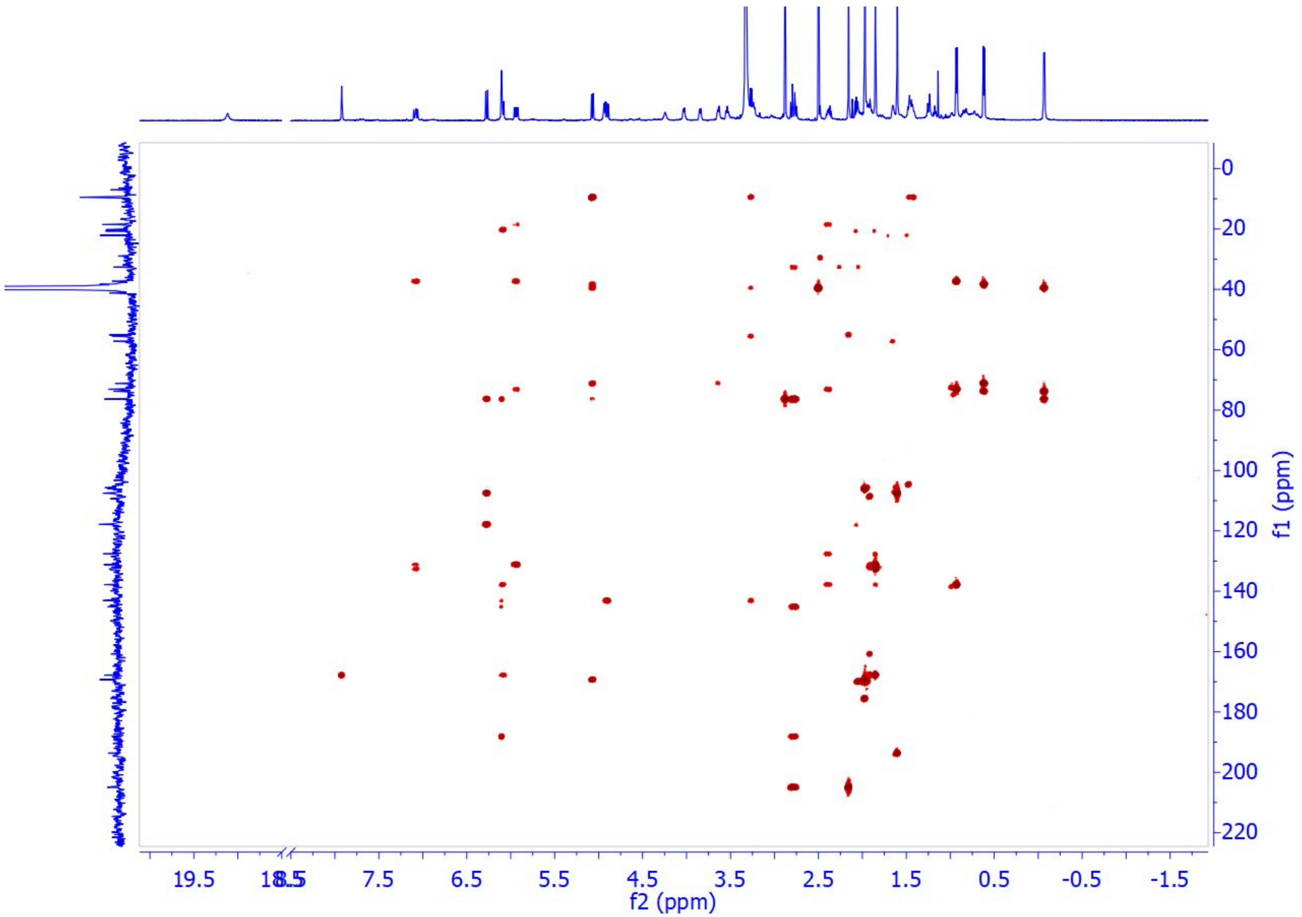

Figure S17. HMBC NMR spectrum of **2** in DMSO-d_6.

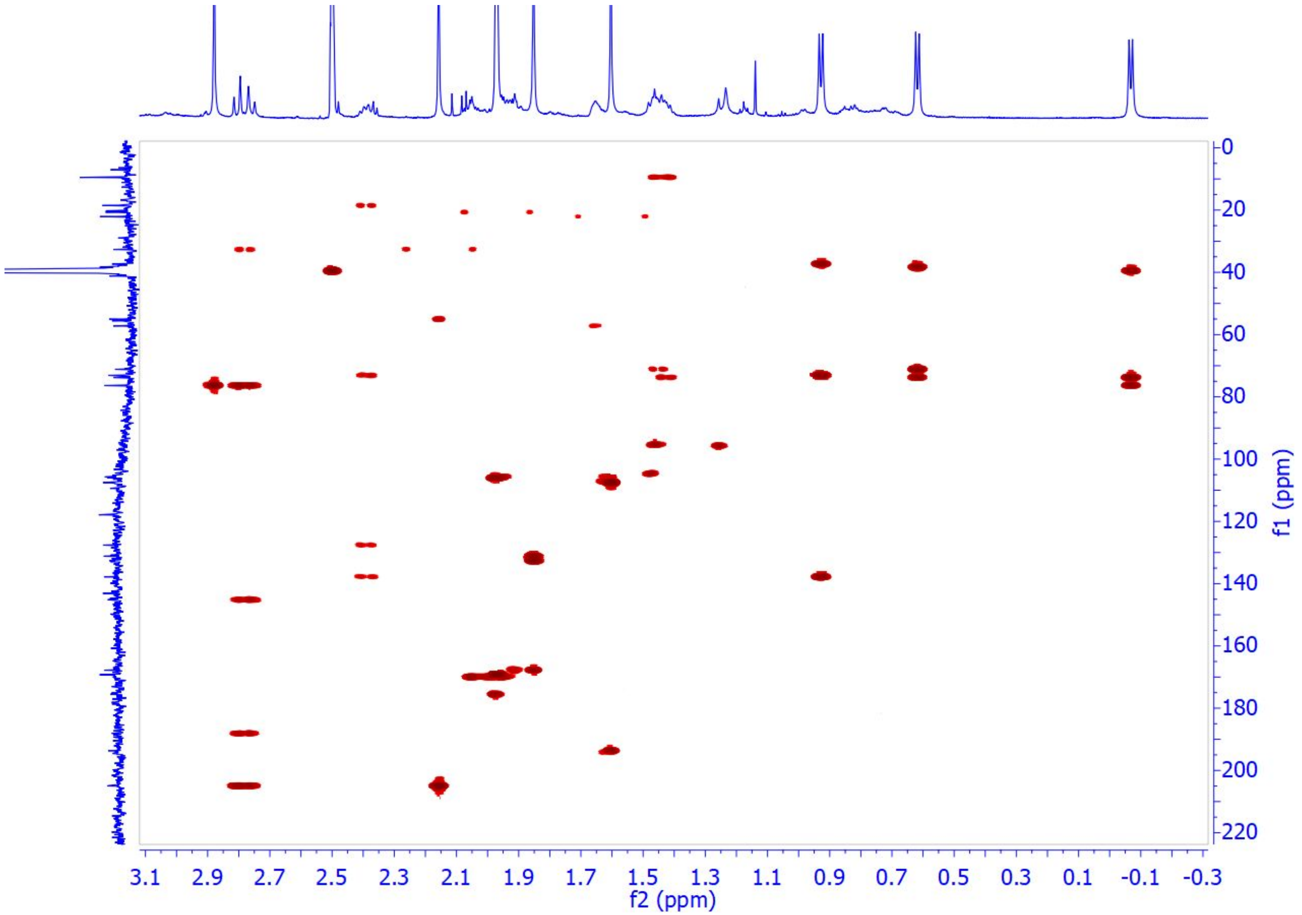

Figure S18. Expanded HMBC NMR spectrum of **2** in DMSO-d_6.

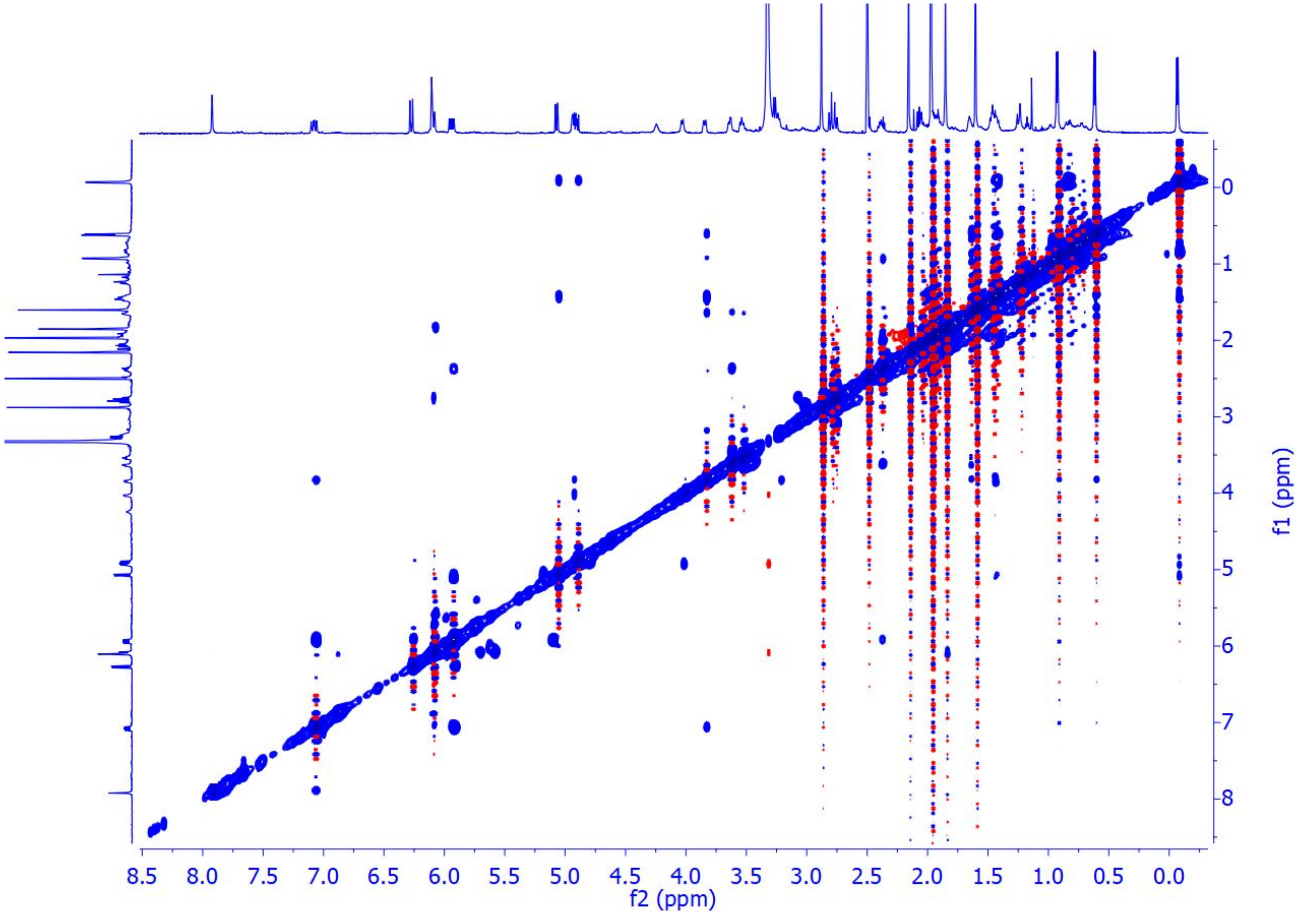

Figure S19. NOESY NMR spectrum of **2** in DMSO-d_6.

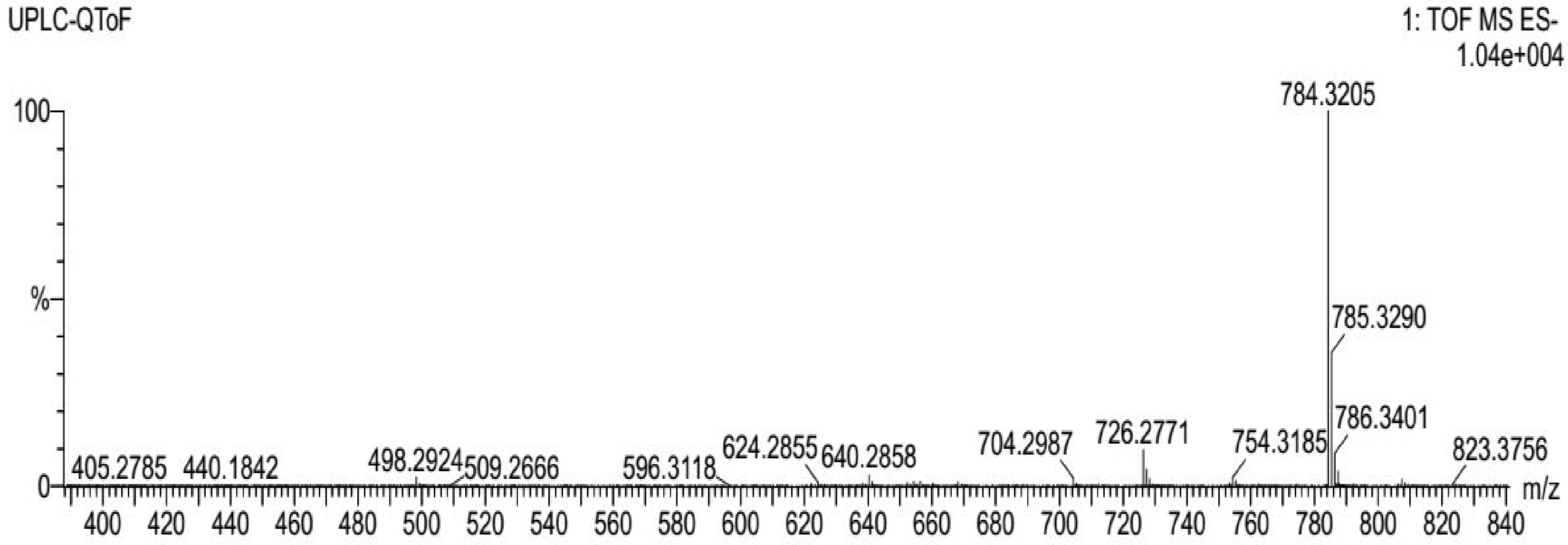

Figure S20. HRESIMS spectrum of **2**.

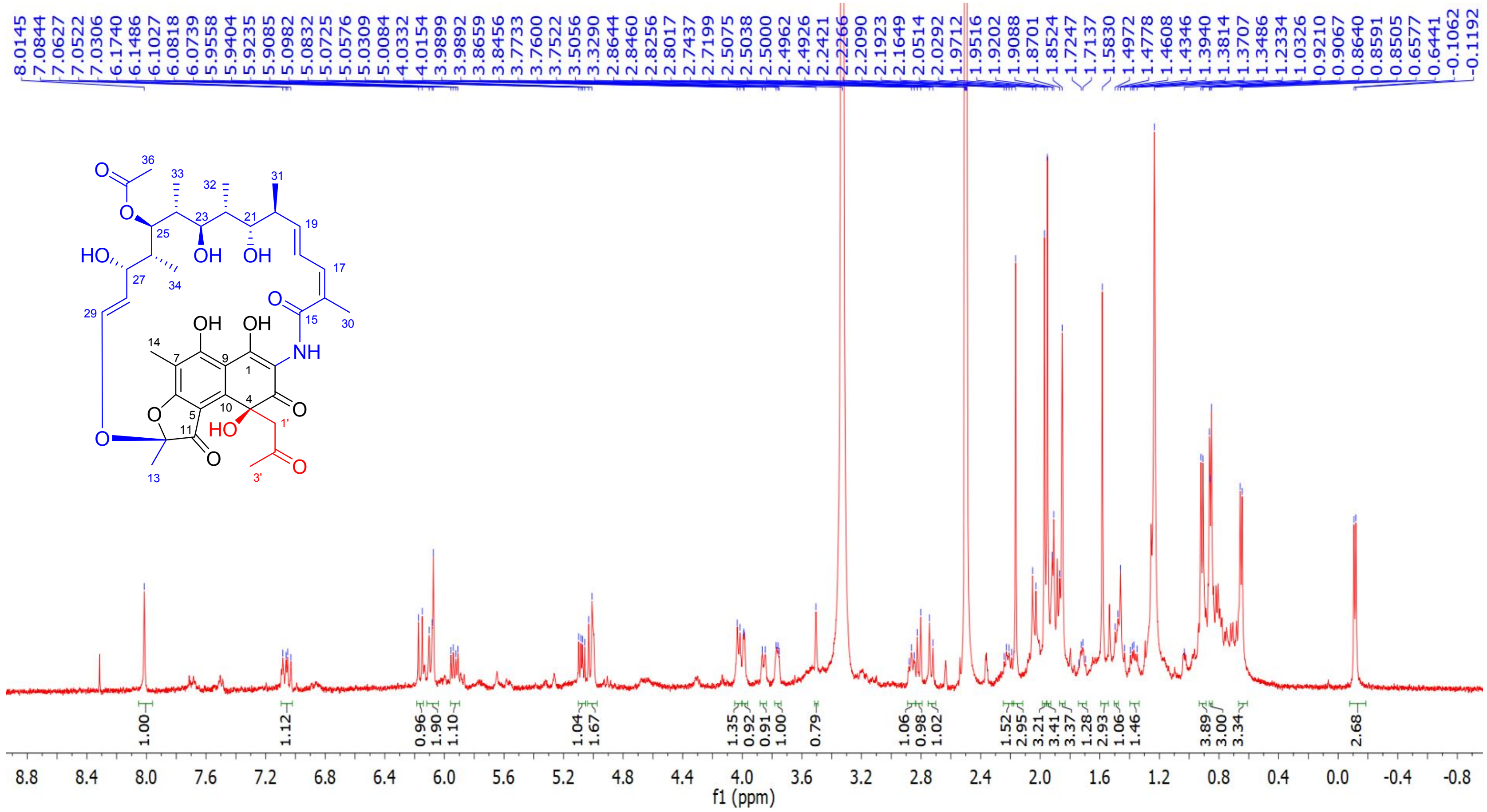

Figure S21. ^{1}H NMR (500 MHz) spectrum of **3** in DMSO-d_6.

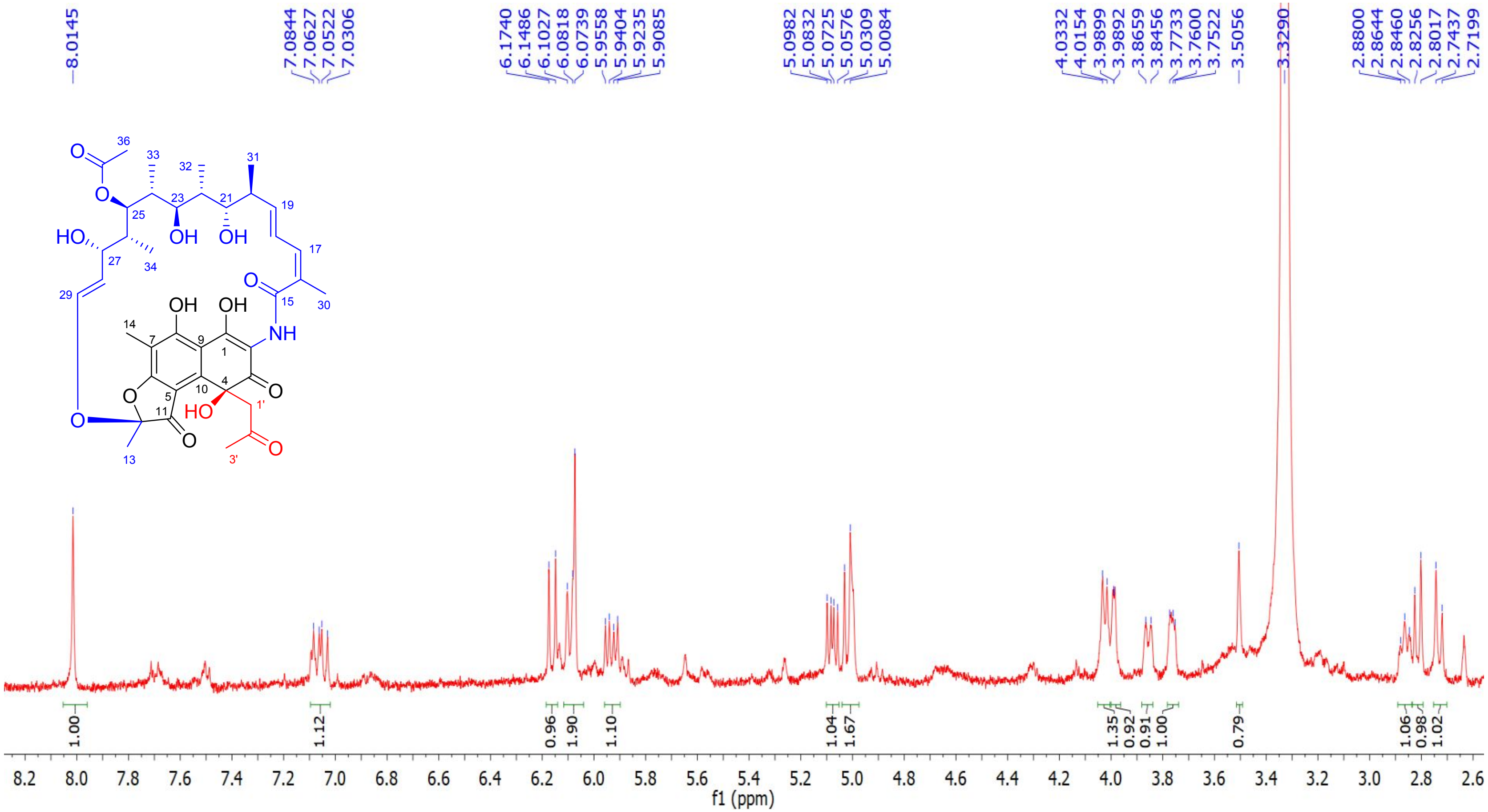

Figure S22. Expanded ^{1}H NMR (500 MHz) spectrum of **3** in DMSO-d_6.

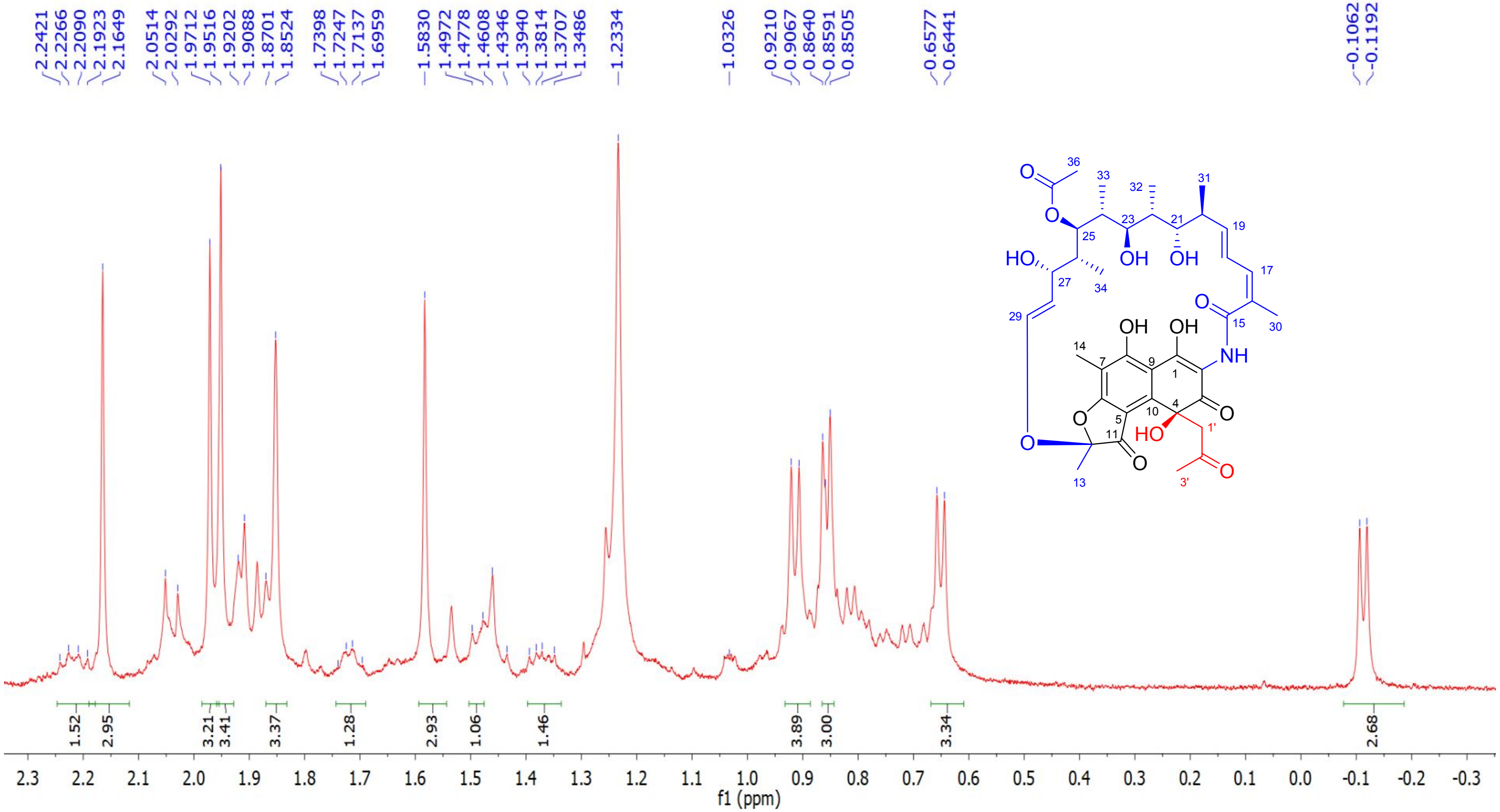

Figure S23. Expanded ^1H NMR (500 MHz) spectrum of **3** in DMSO-d_6.

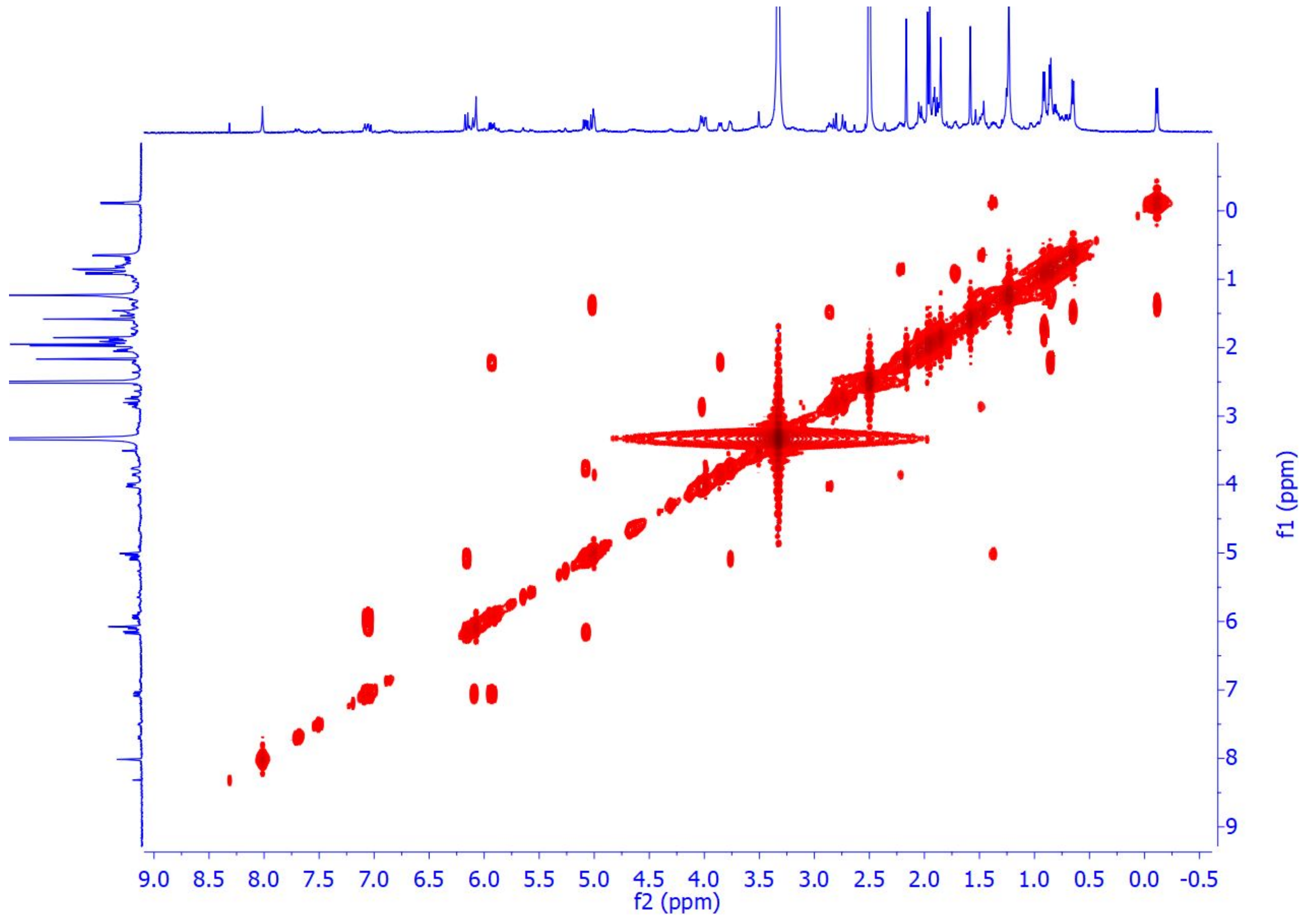

Figure S24. COSY NMR spectrum of **3** in DMSO-d_6.

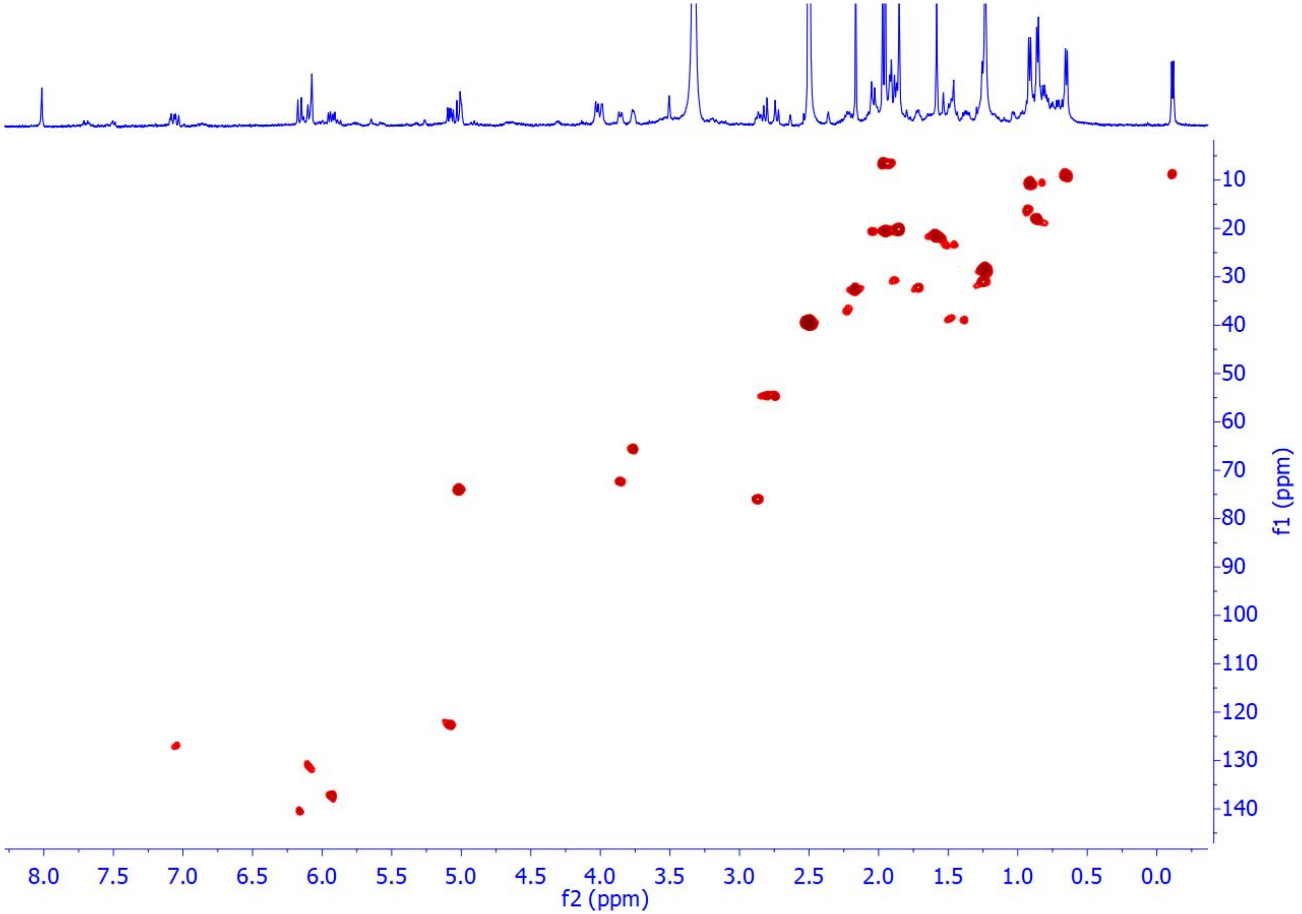

Figure S25. HSQC NMR spectrum of **3** in DMSO-d_6.

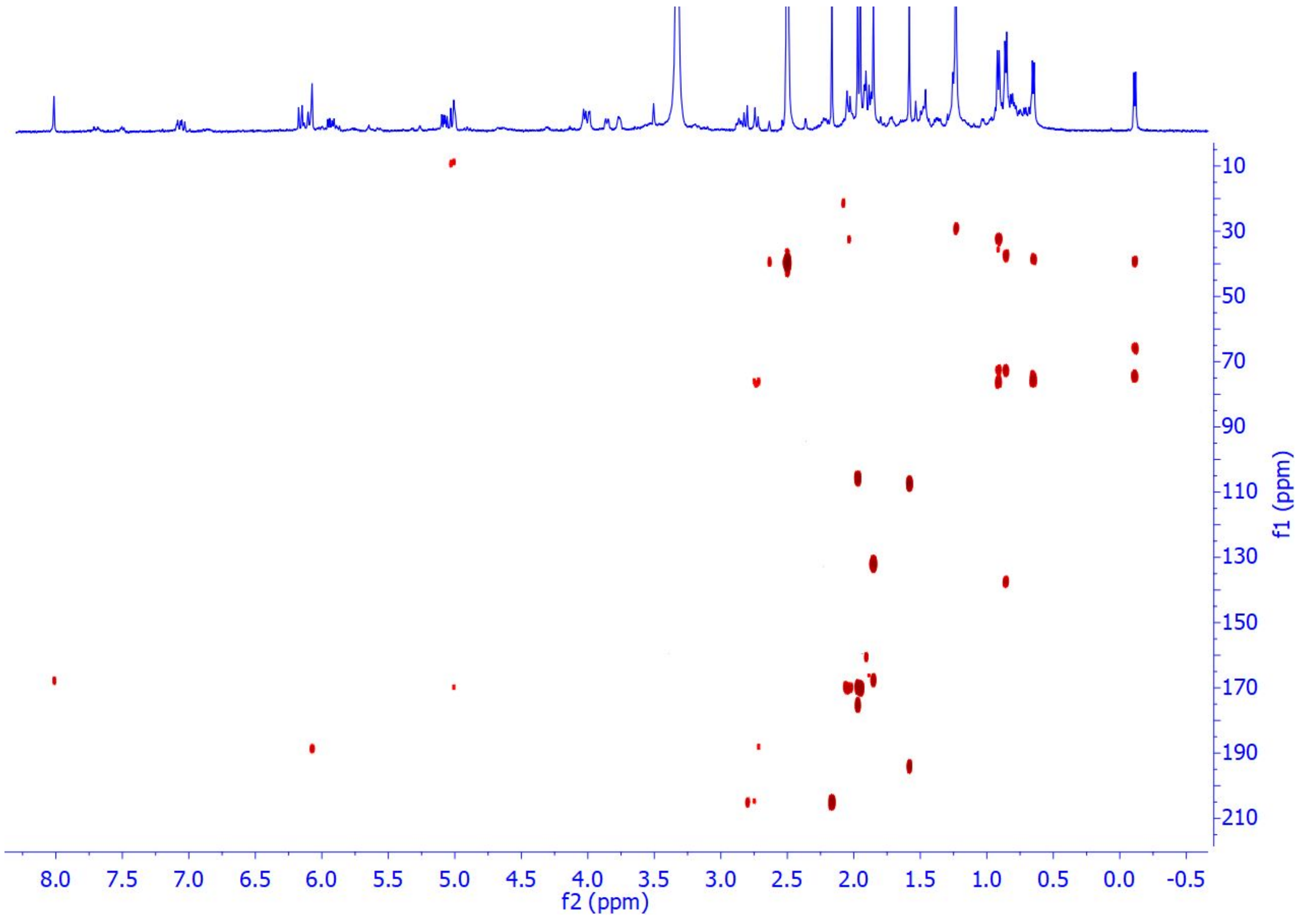

Figure S26. HMBC NMR spectrum of **3** in DMSO-d_6.

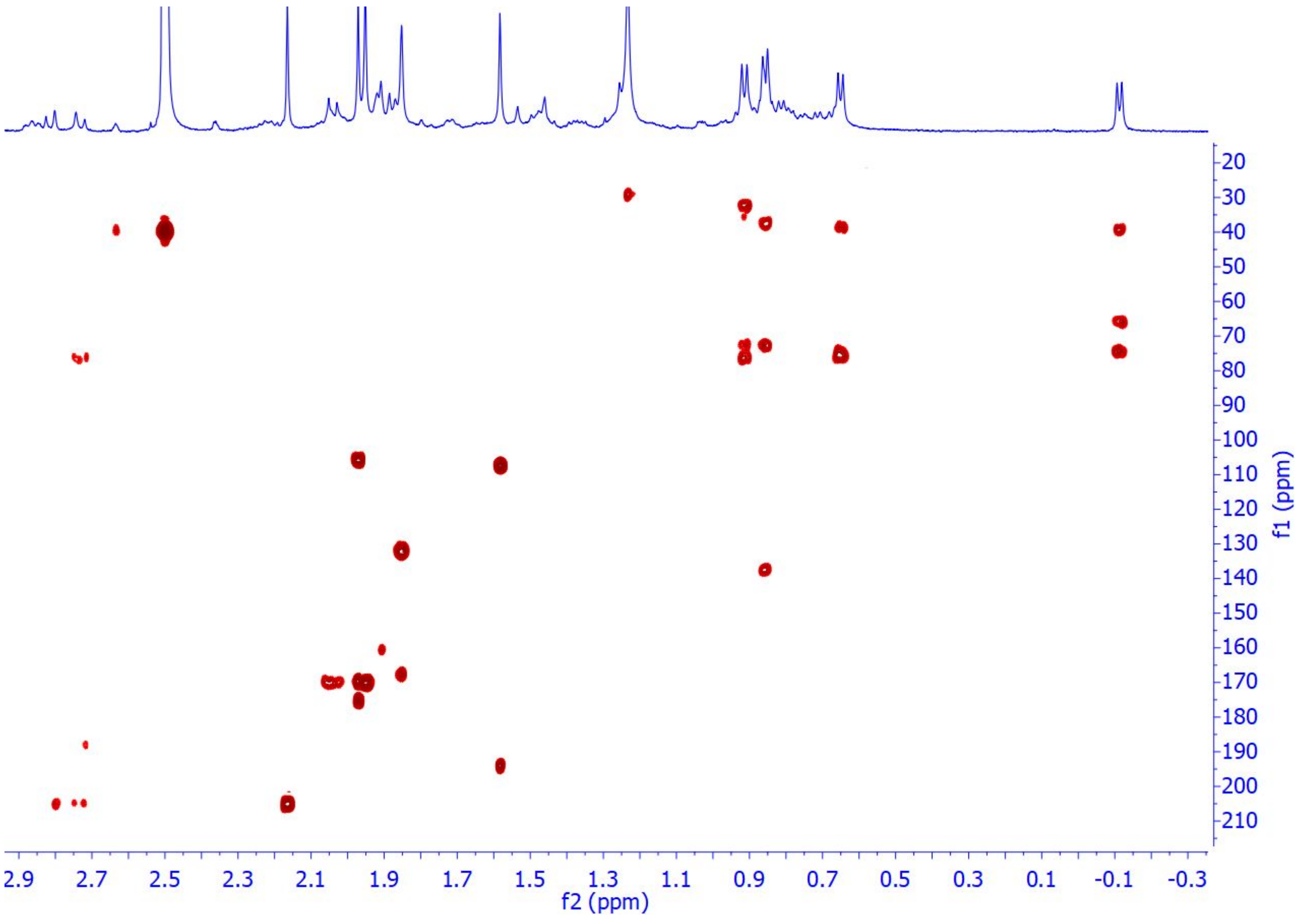

Figure S27. Expanded HMBC NMR spectrum of **3** in DMSO-d_6.

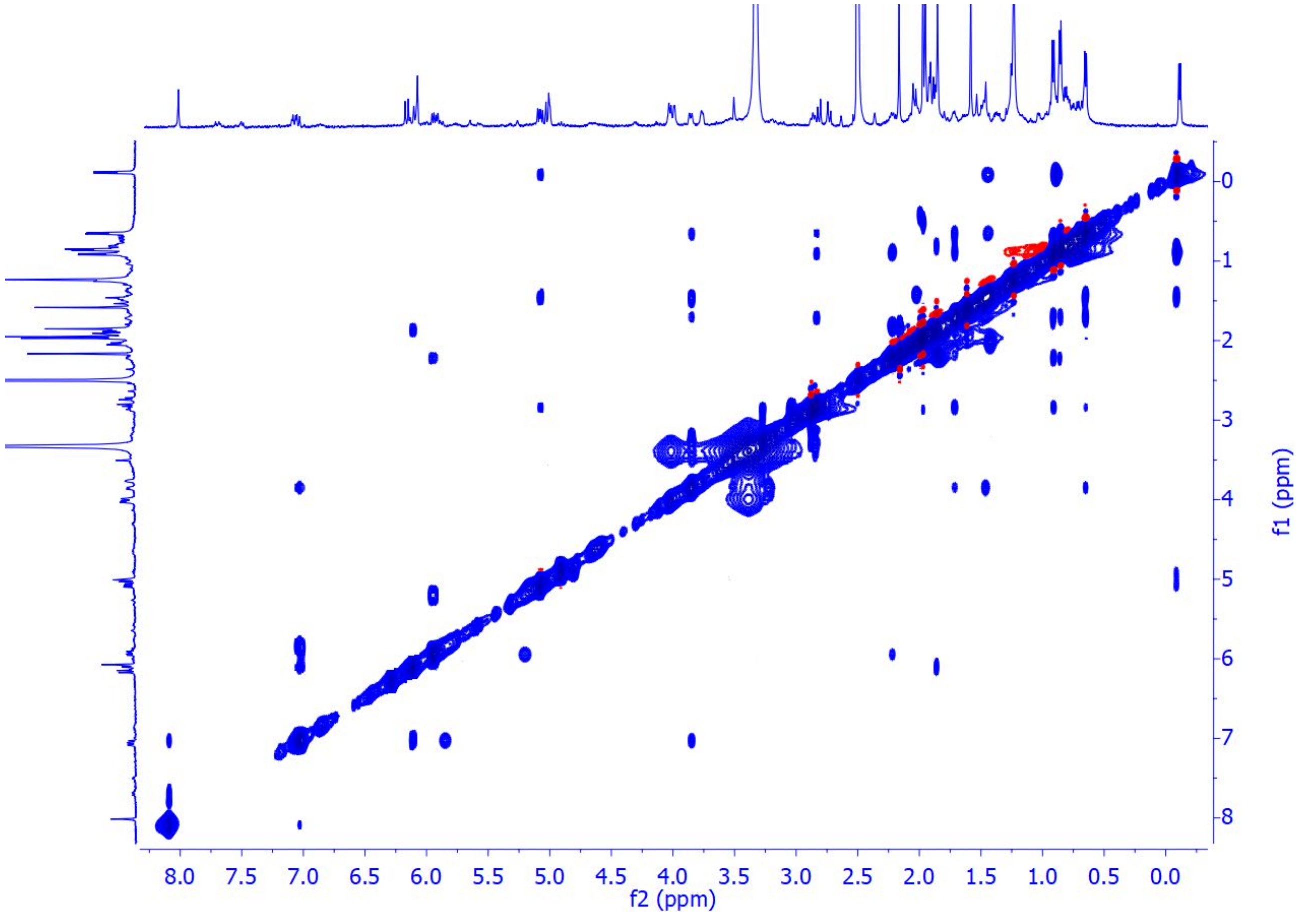

Figure S28. NOESY NMR spectrum of **3** in DMSO-d_6.

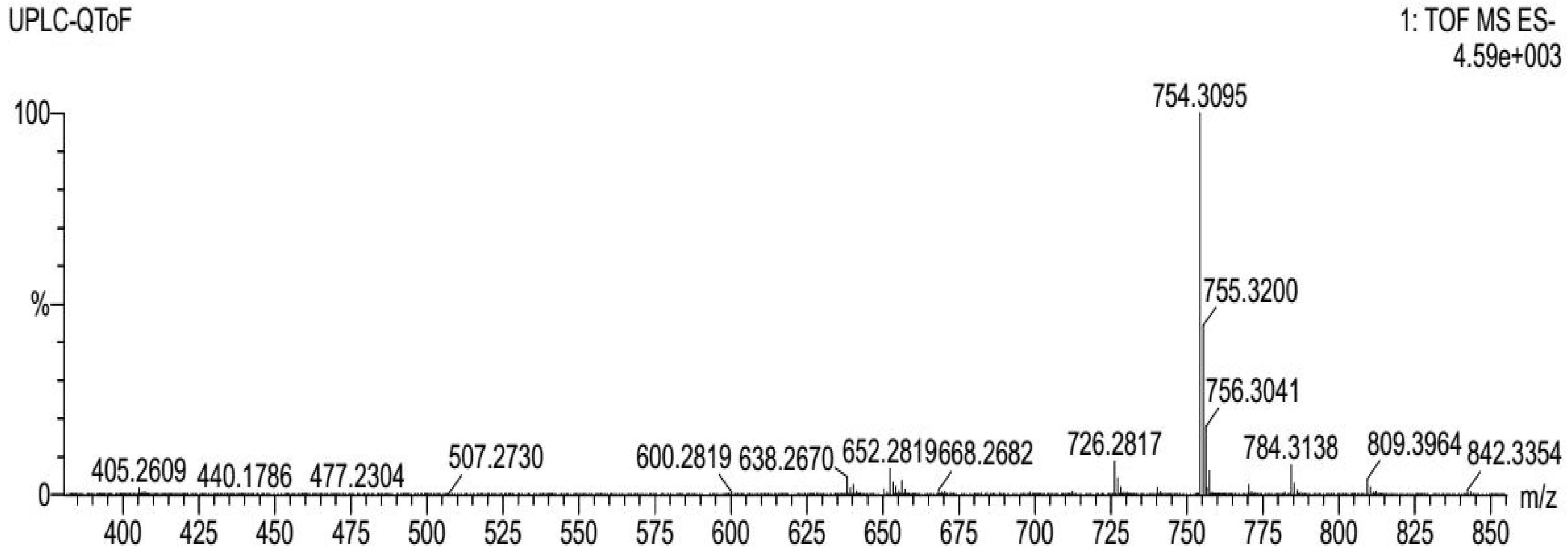

Figure S29. HRESIMS spectrum of **3**.

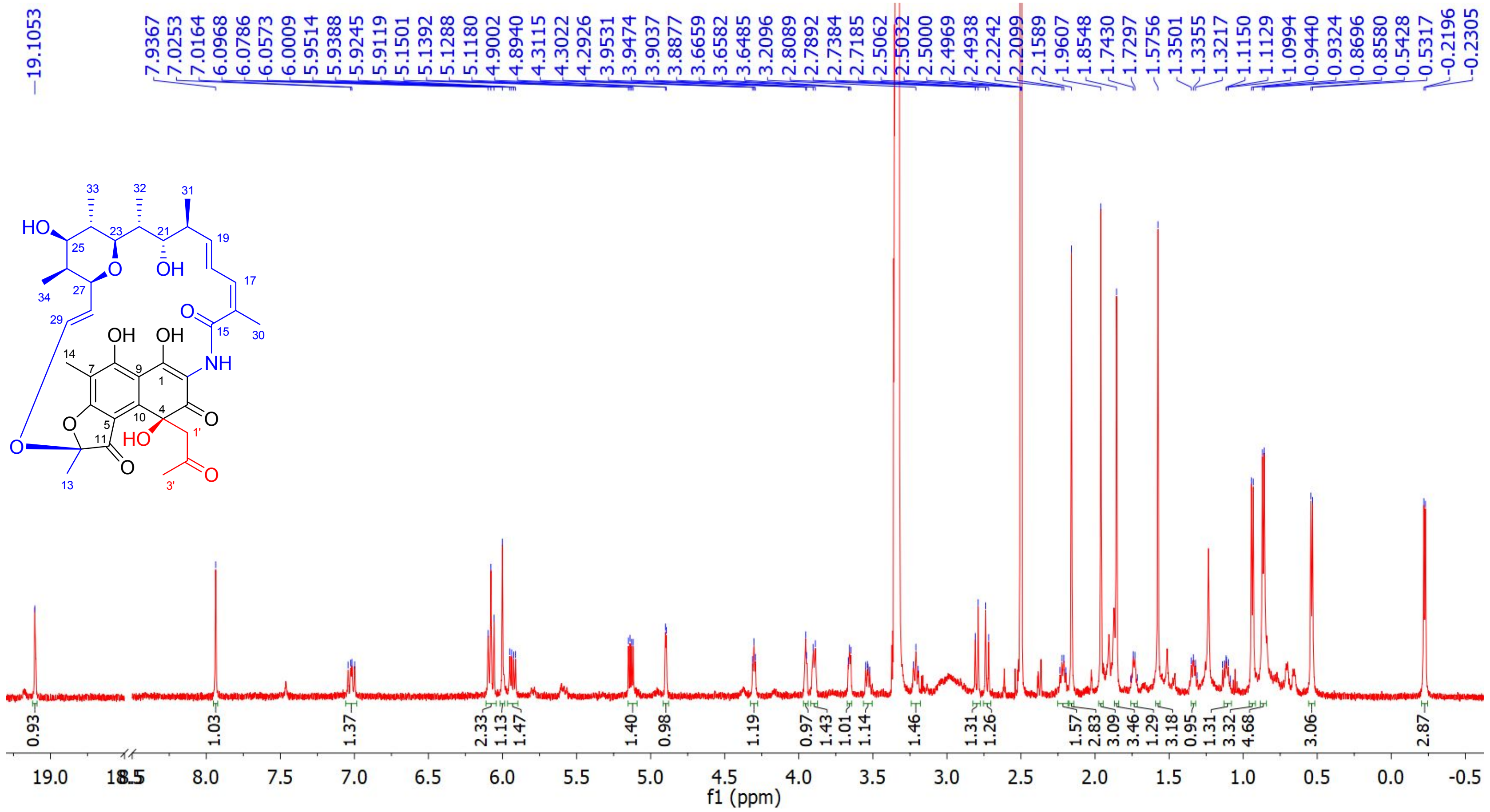

Figure S30. ^{1}H NMR (600 MHz) spectrum of **4** in DMSO-d_6.

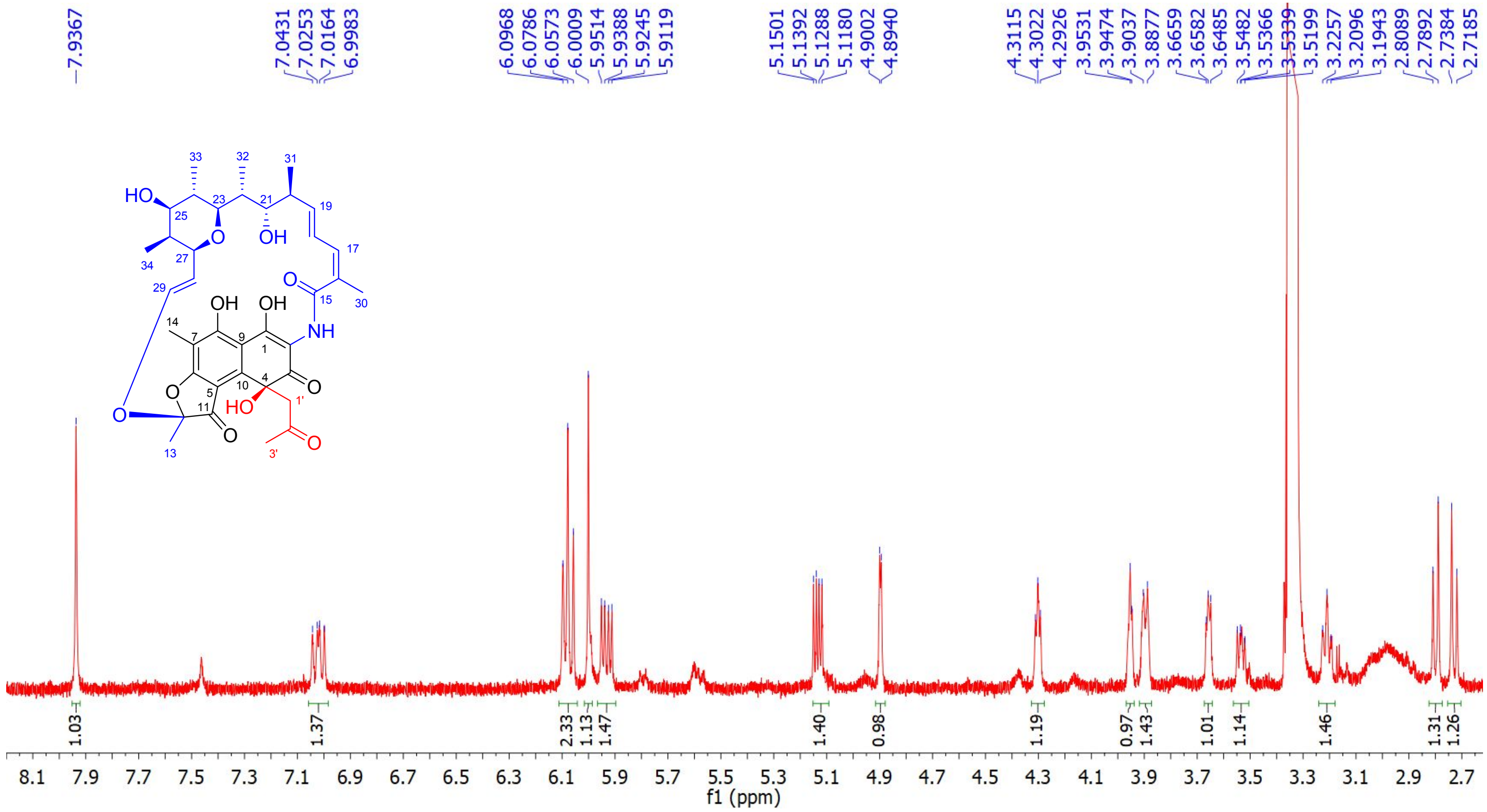

Figure S31. Expanded ^{1}H NMR (600 MHz) spectrum of **4** in DMSO-d_6.

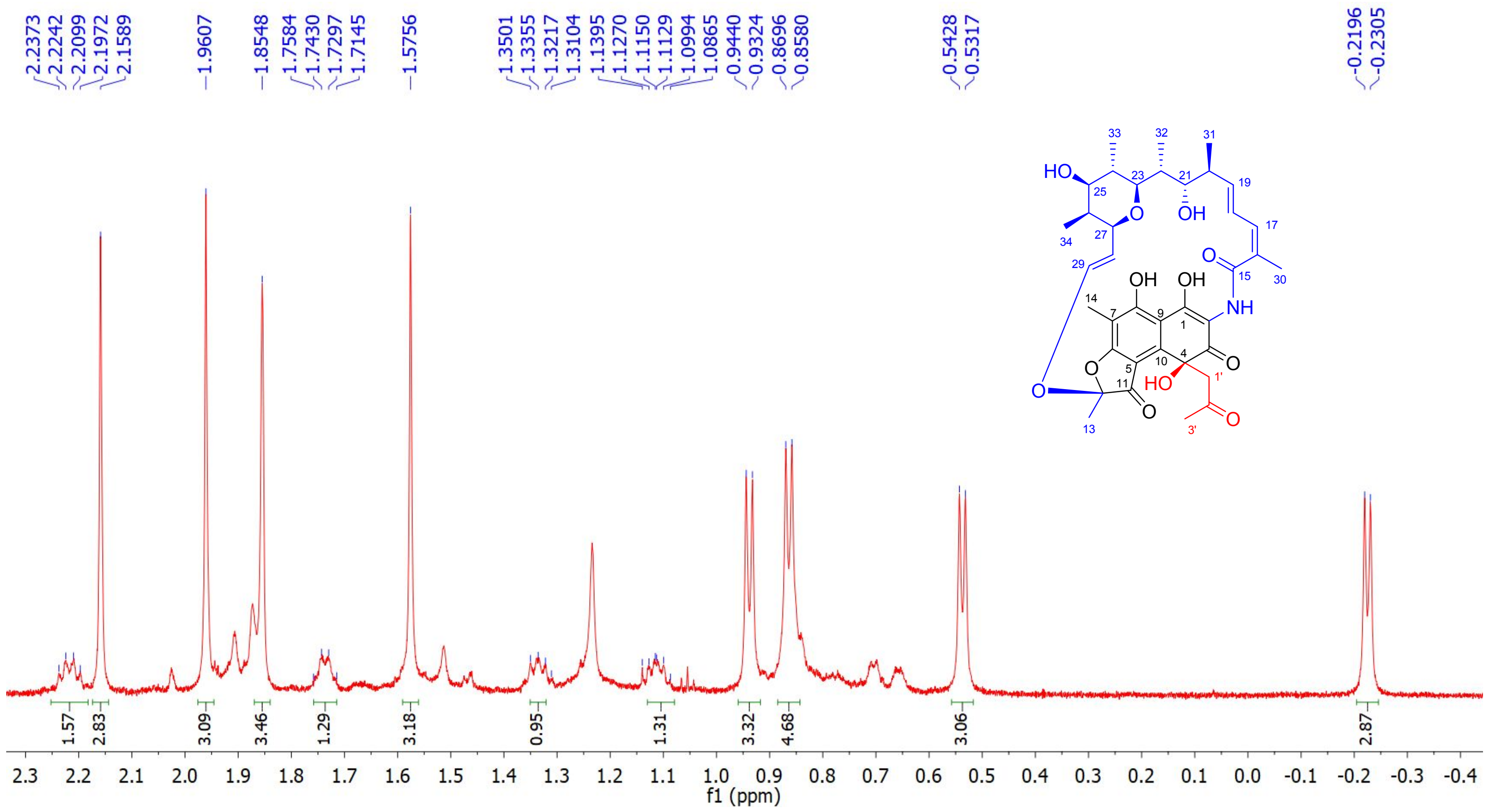

Figure S32. Expanded ^{1}H NMR (600 MHz) spectrum of **4** in DMSO-d_6.

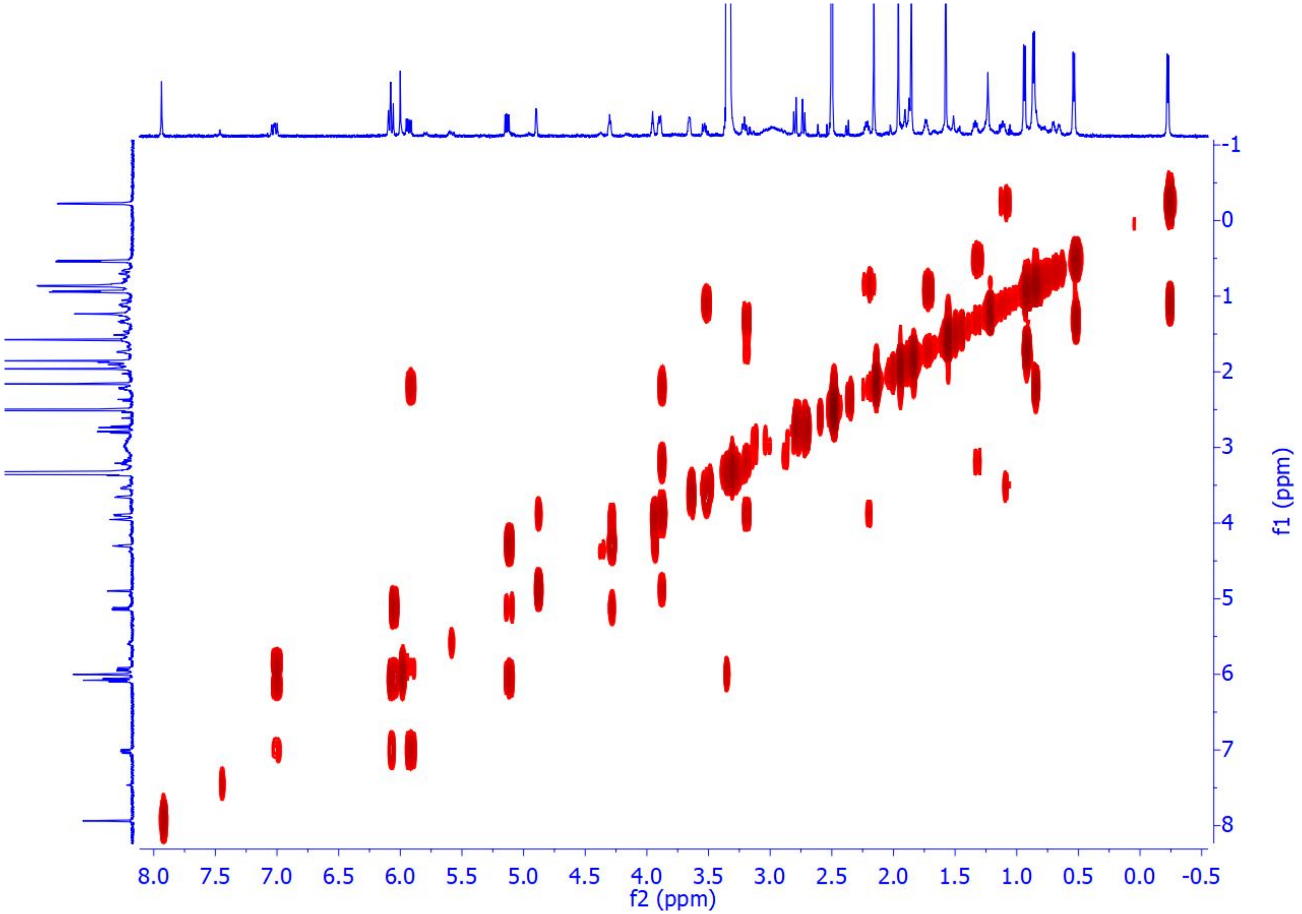

Figure S33. COSY NMR spectrum of **4** in DMSO-d_6.

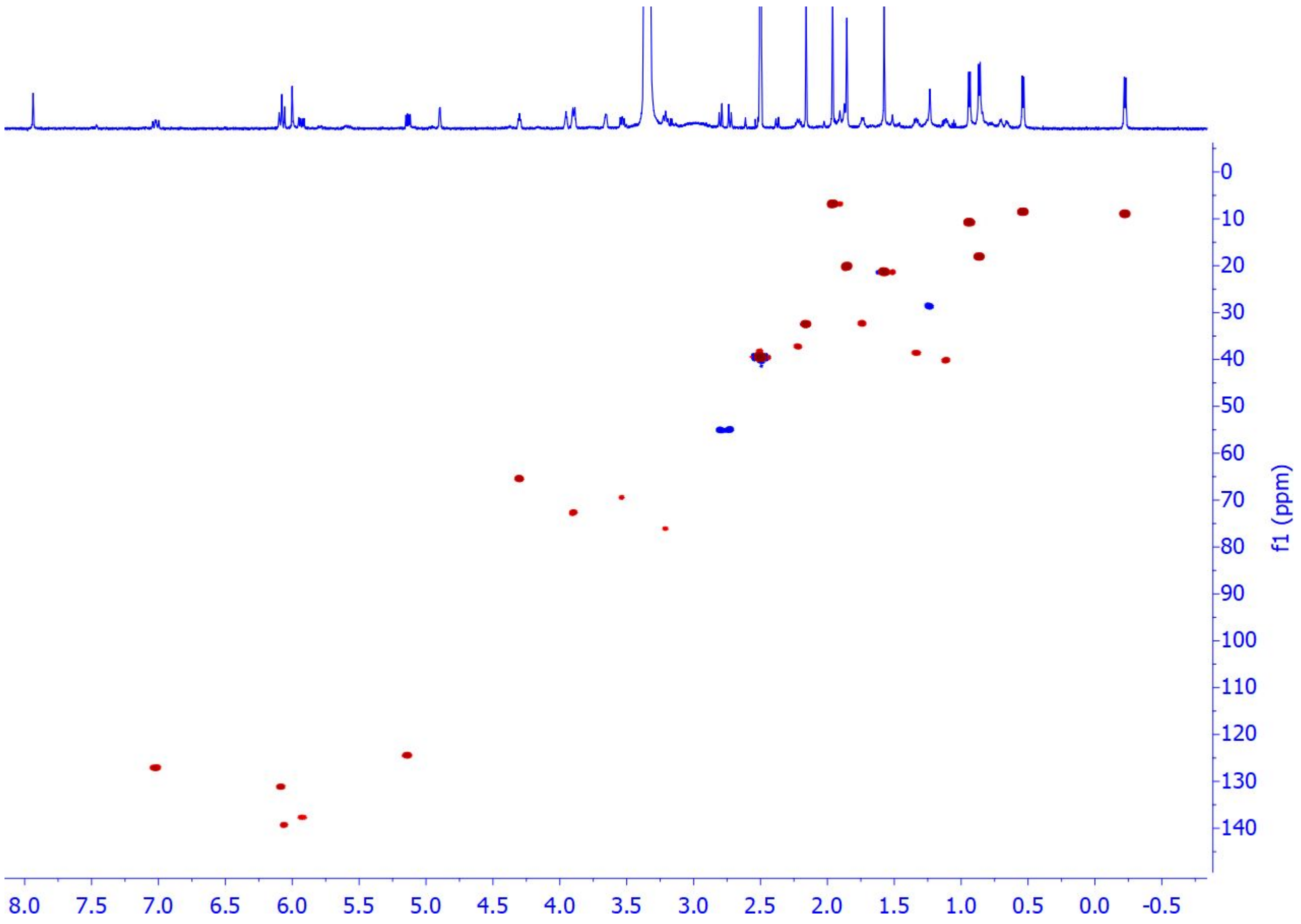

Figure S34. Edited HSQC NMR spectrum of **4** in DMSO-d_6.

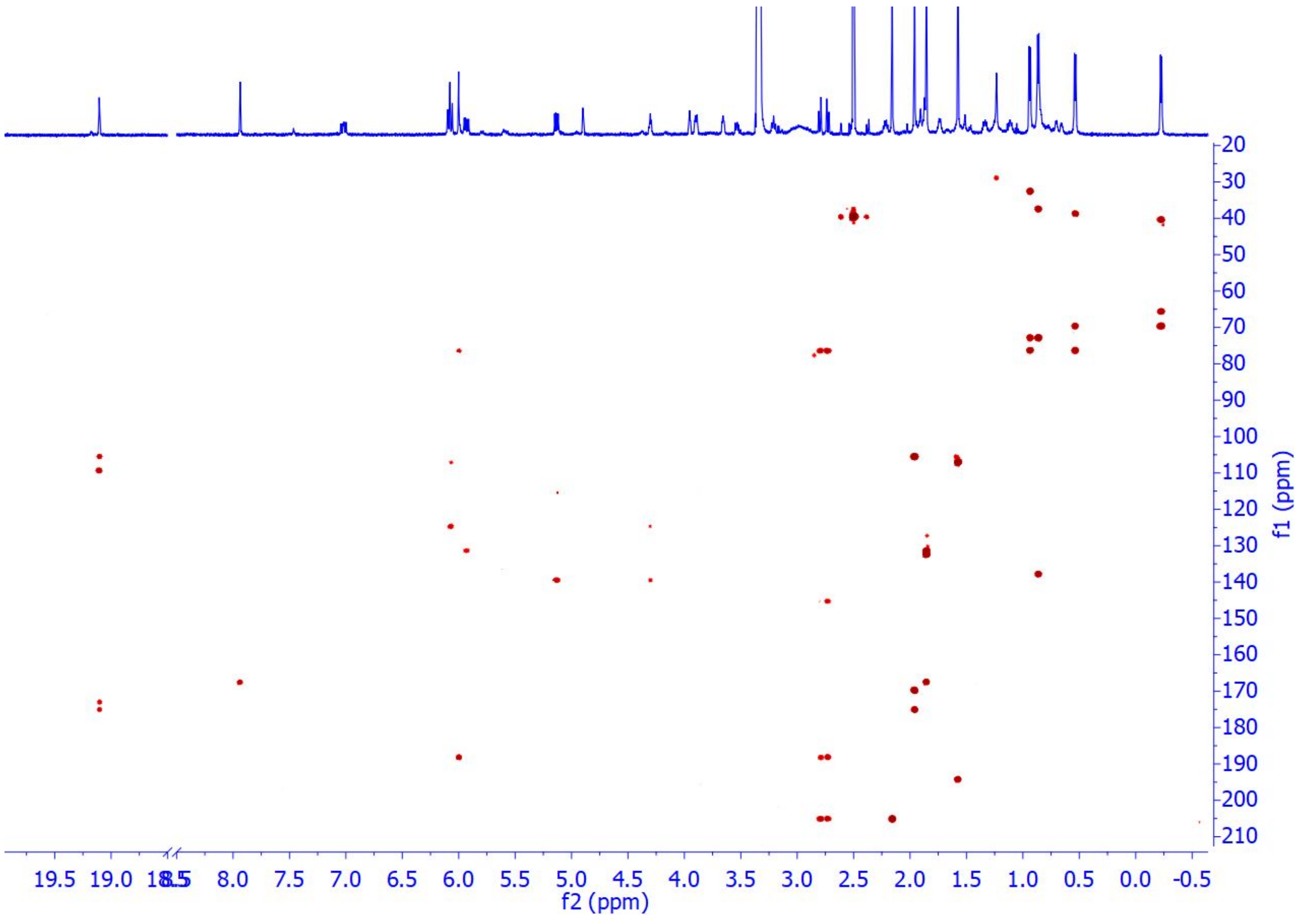

Figure S35. HMBC NMR spectrum of **4** in DMSO-d_6.

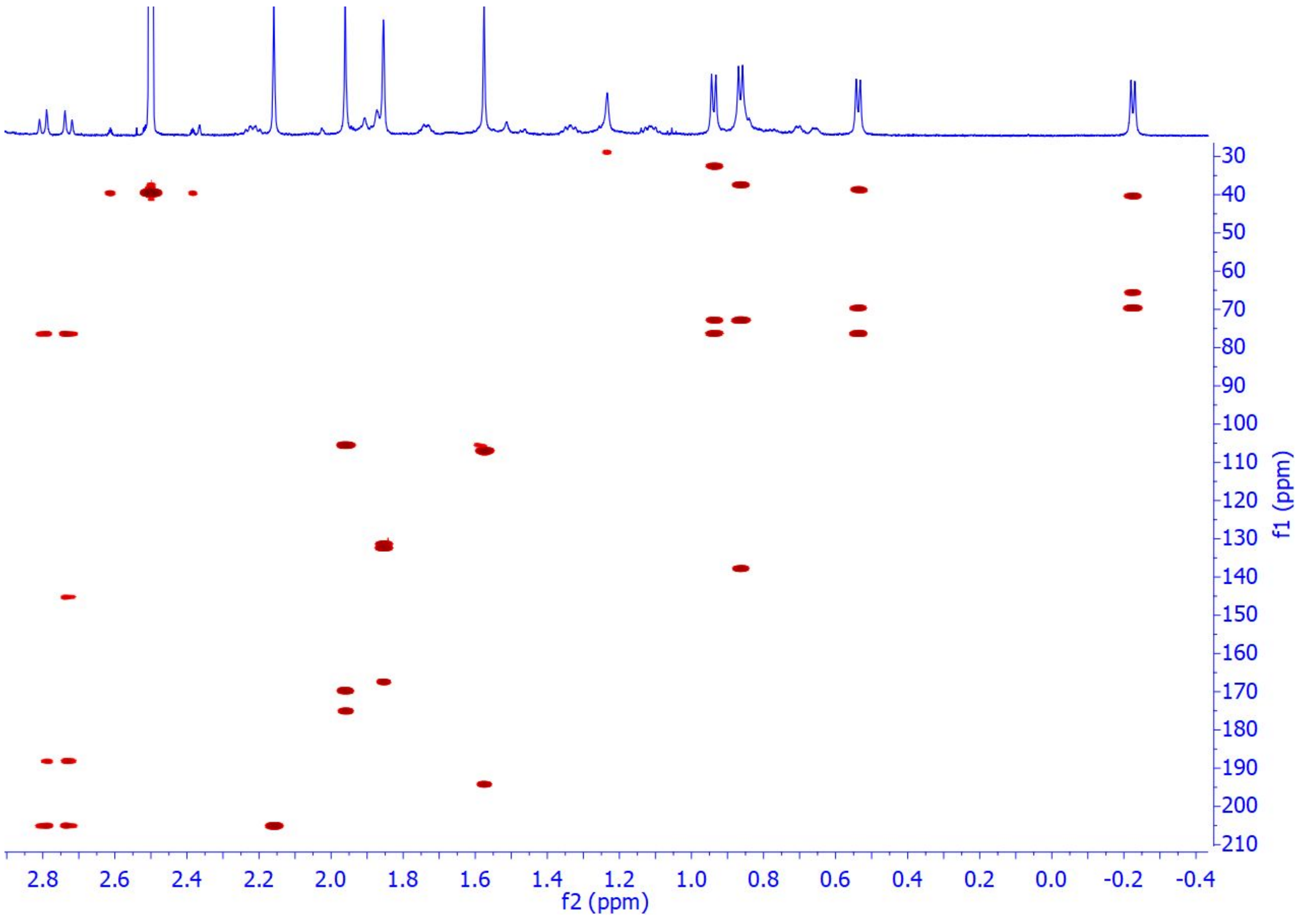

Figure S36. Expanded HMBC NMR spectrum of **4** in DMSO-d_6.

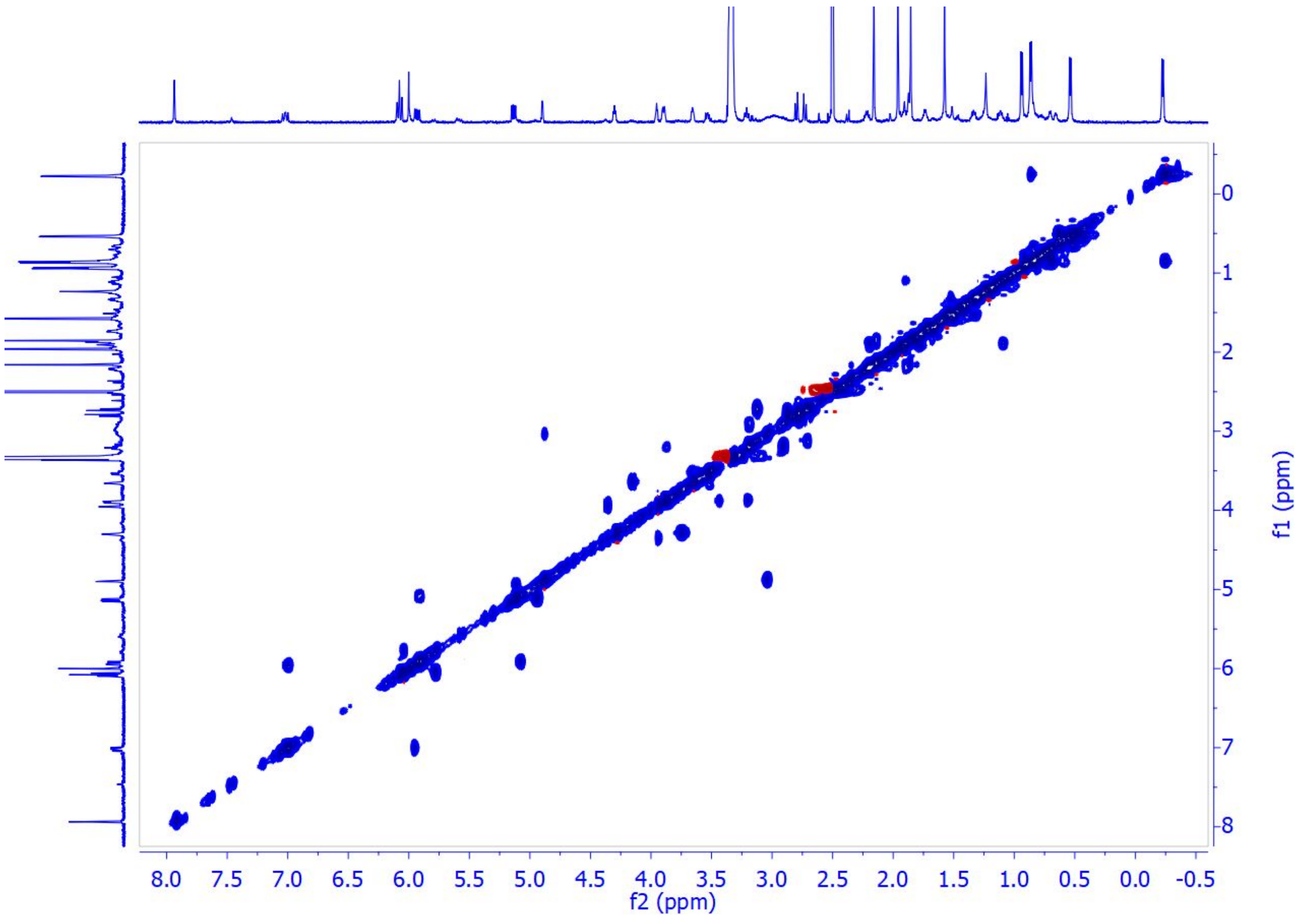

Figure S37. NOESY NMR spectrum of **4** in DMSO-d_6.

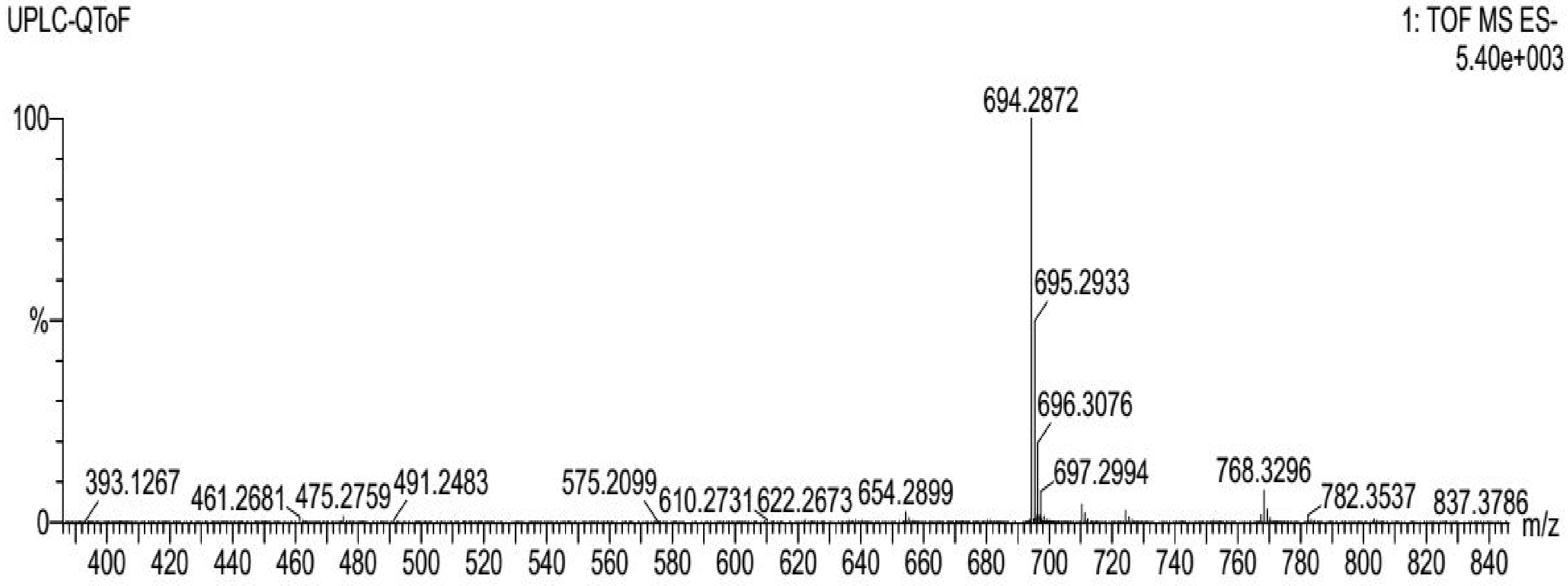

Figure S38. HRESIMS spectrum of **4**.

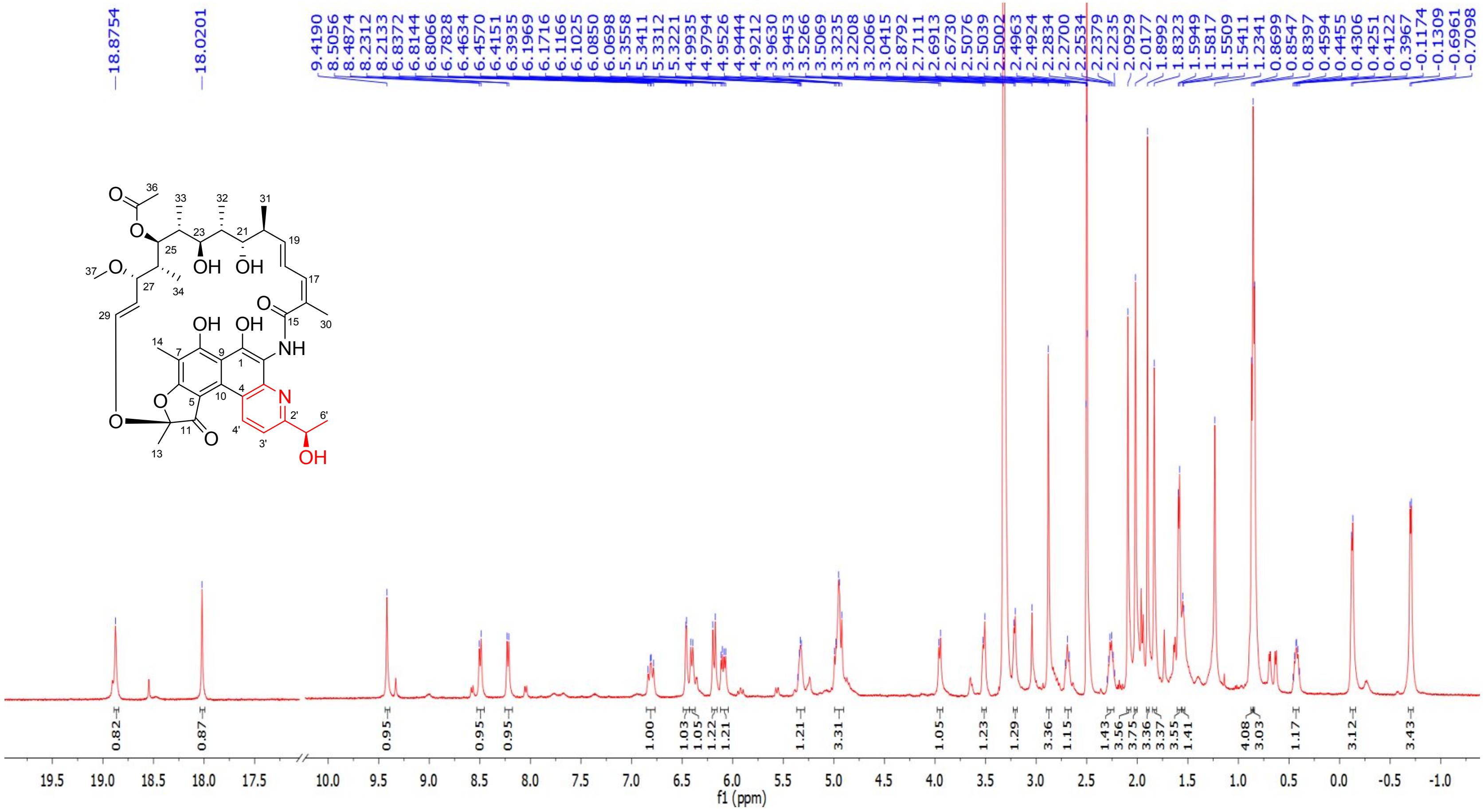

Figure S39. ^{1}H NMR (500 MHz) spectrum of **5** in DMSO-d_6.

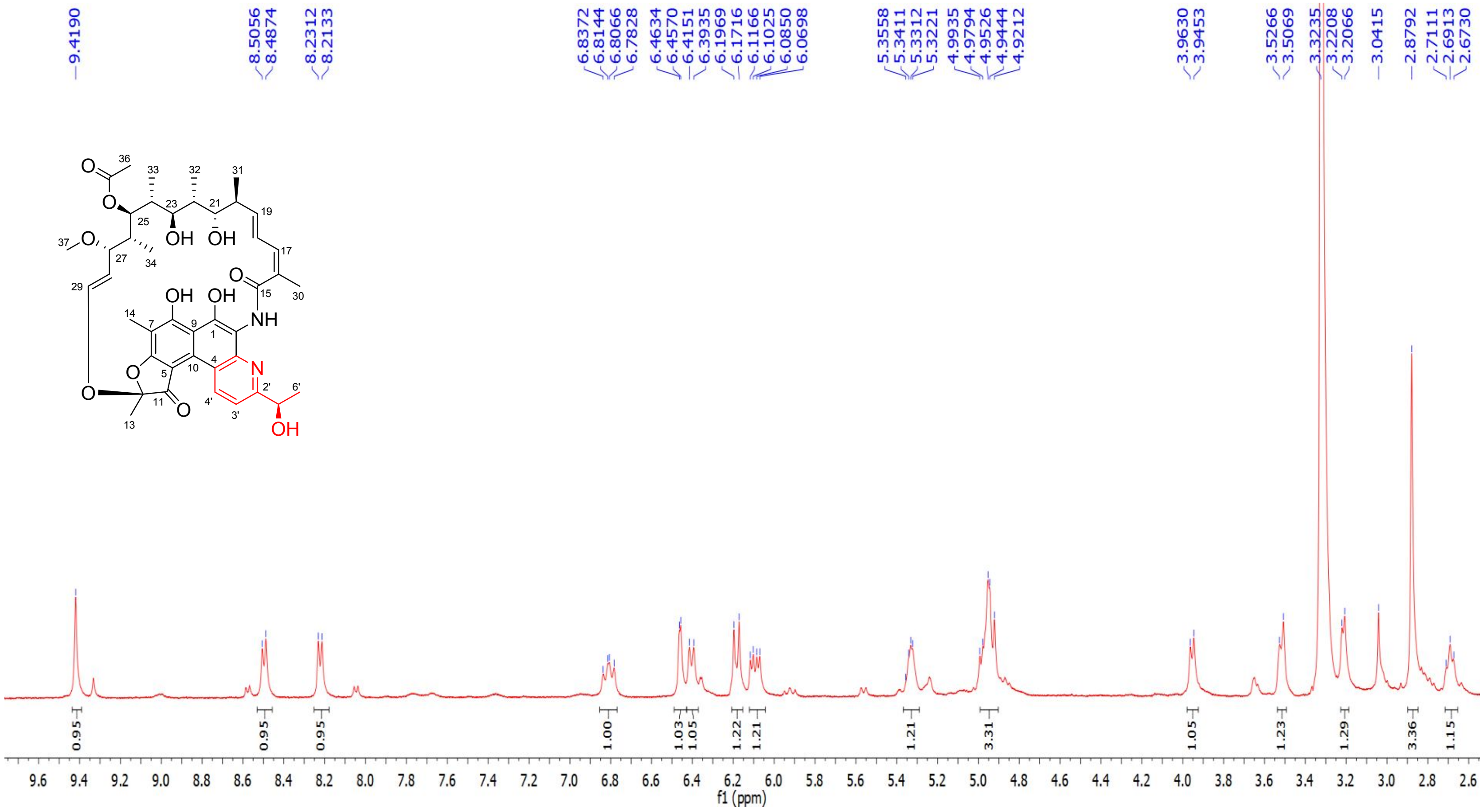

Figure S40. Expanded ^{1}H NMR (500 MHz) spectrum of **5** in DMSO-d_6.

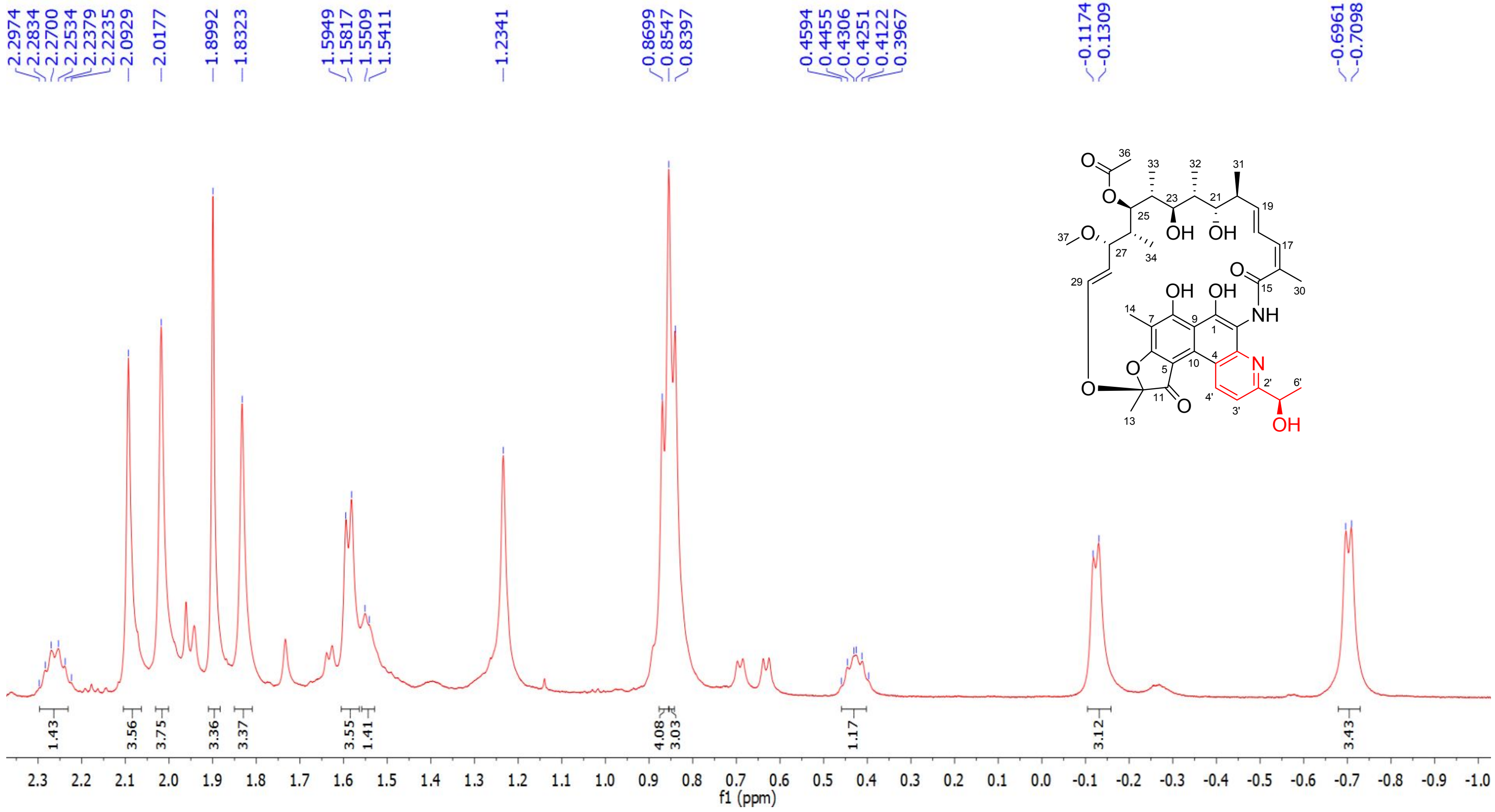

Figure S41. Expanded ^{1}H NMR (500 MHz) spectrum of **5** in DMSO-d_6.

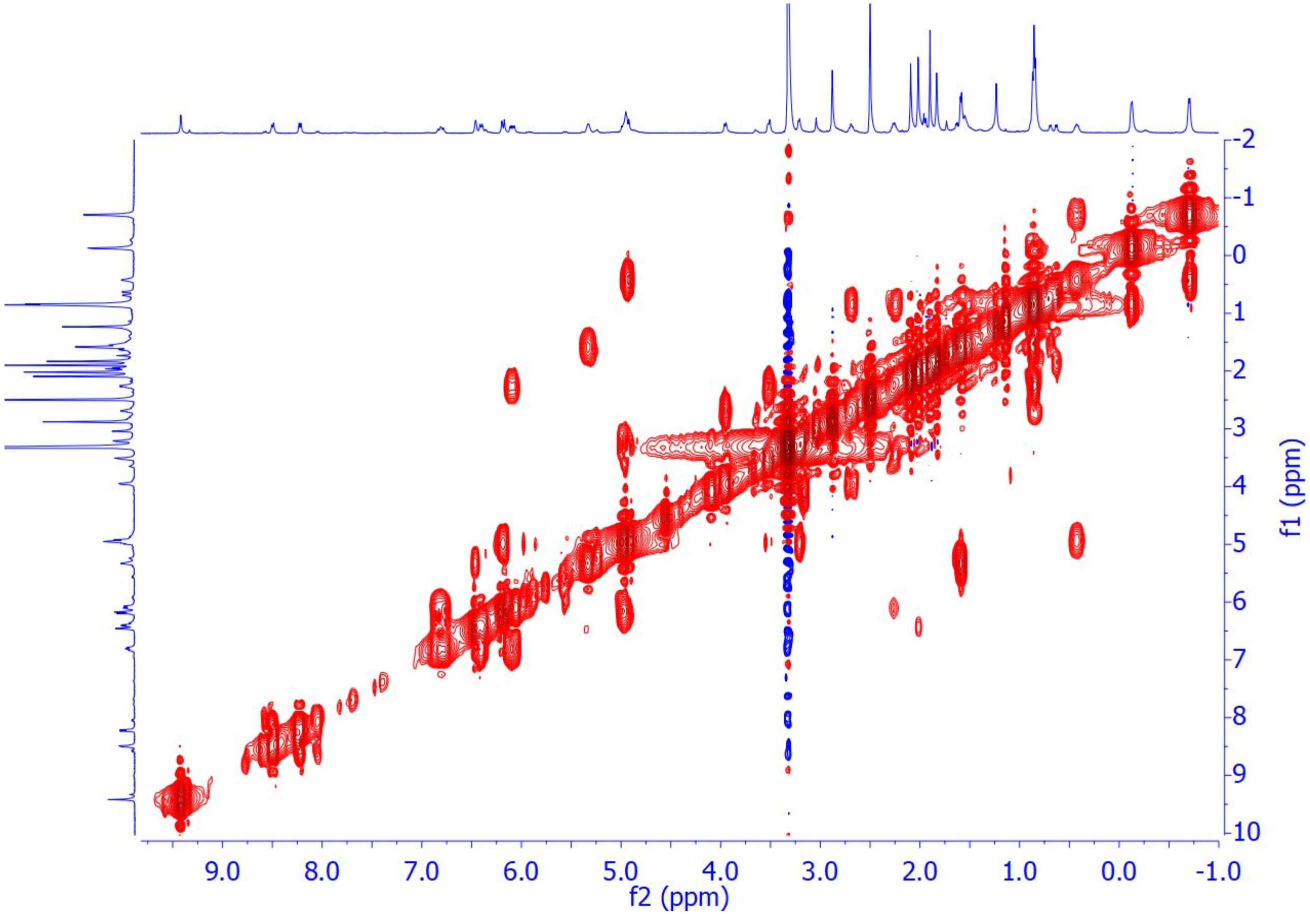

Figure S42. COSY NMR spectrum of **5** in DMSO-d_6.

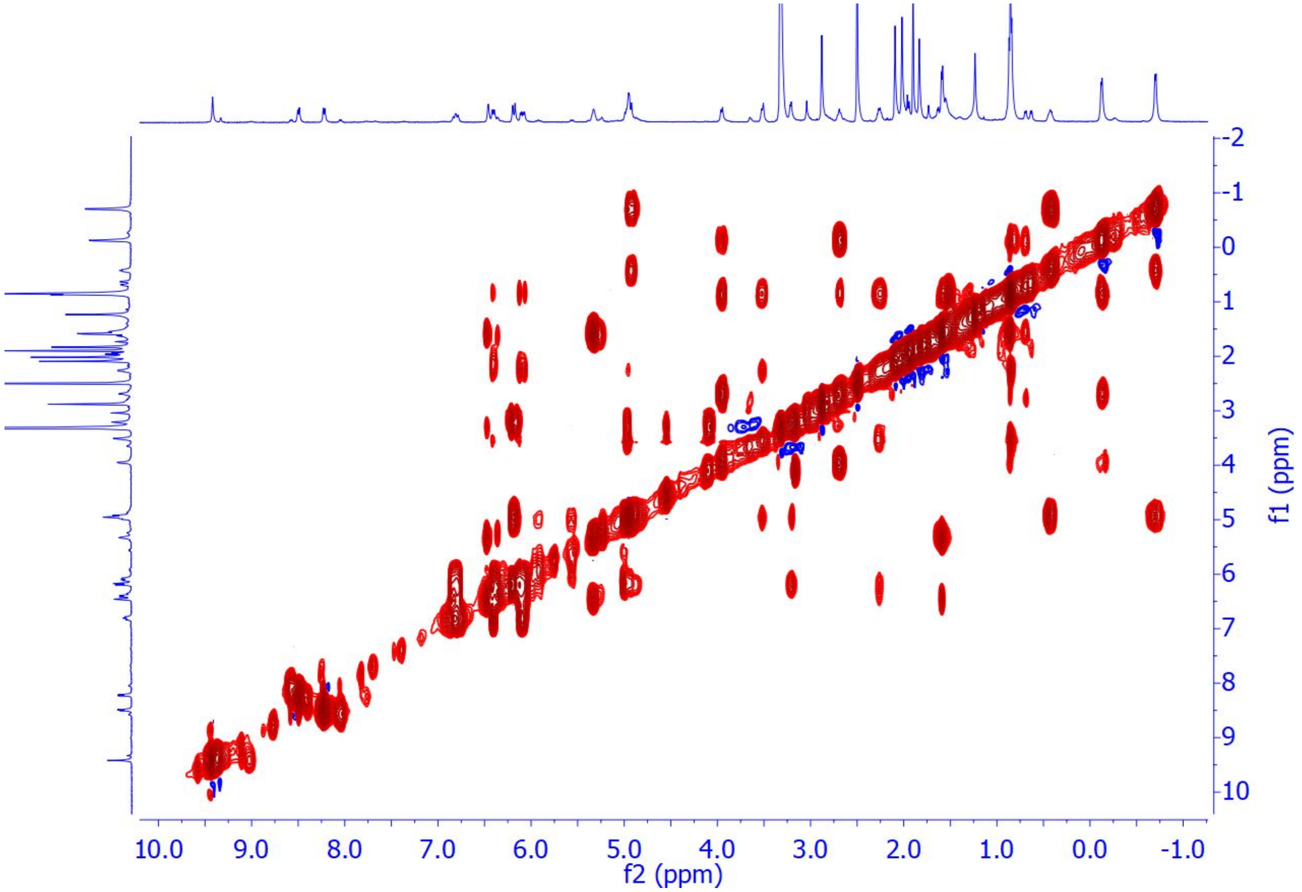

Figure S43. TOCSY NMR spectrum of **5** in DMSO-d_6.

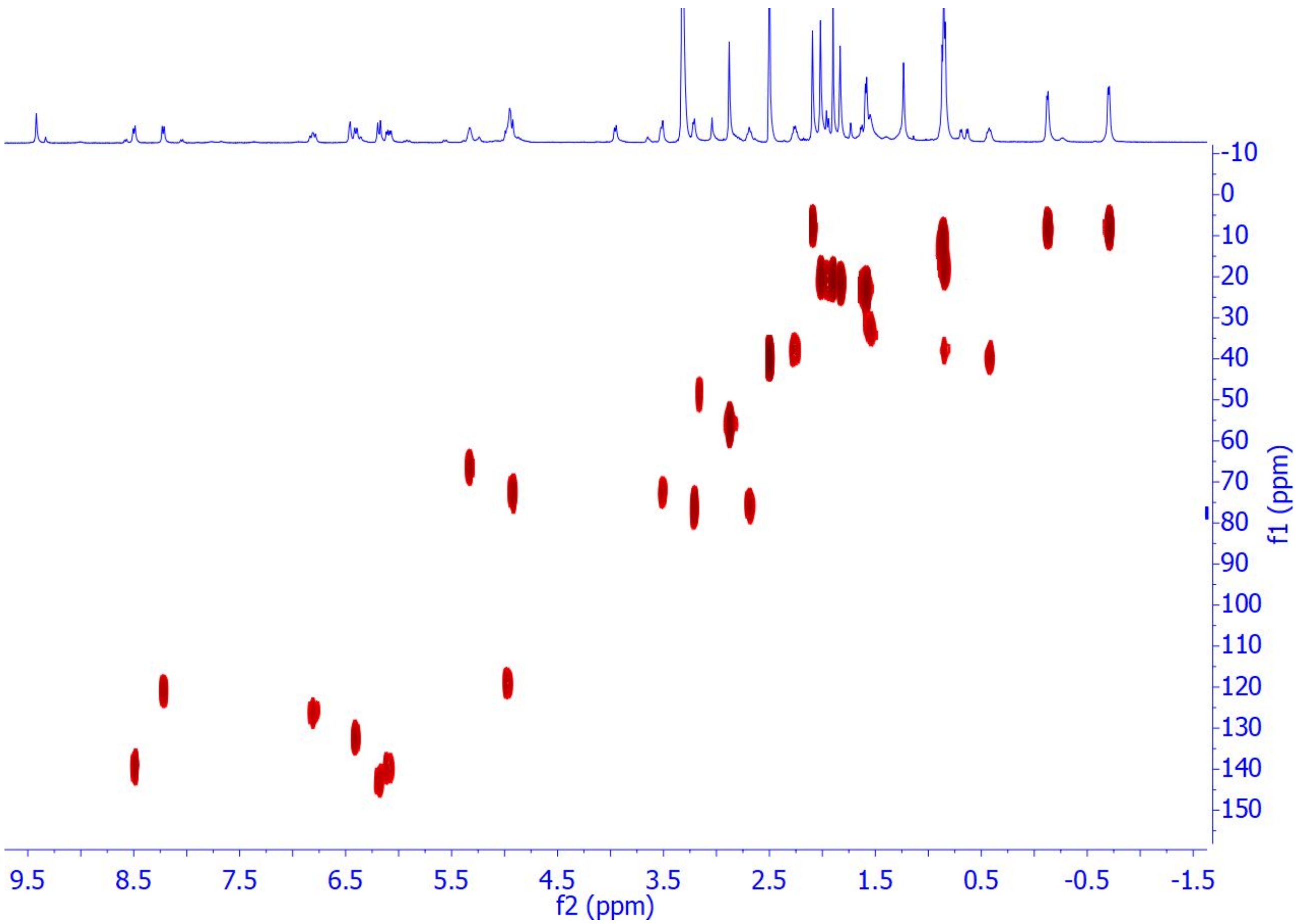

Figure S44. HSQC NMR spectrum of **5** in DMSO-d_6.

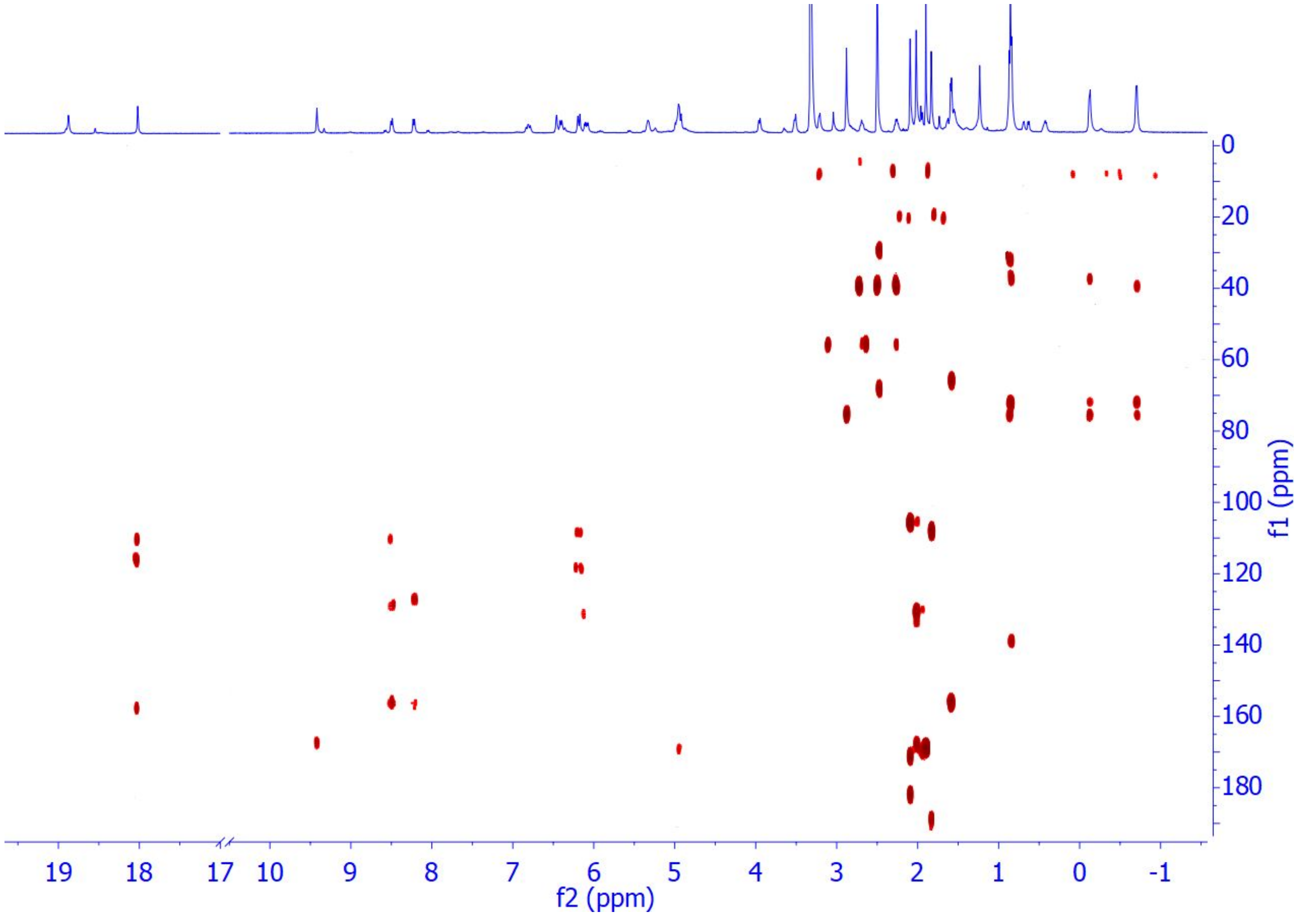

Figure S45. HMBC NMR spectrum of **5** in DMSO-d_6.

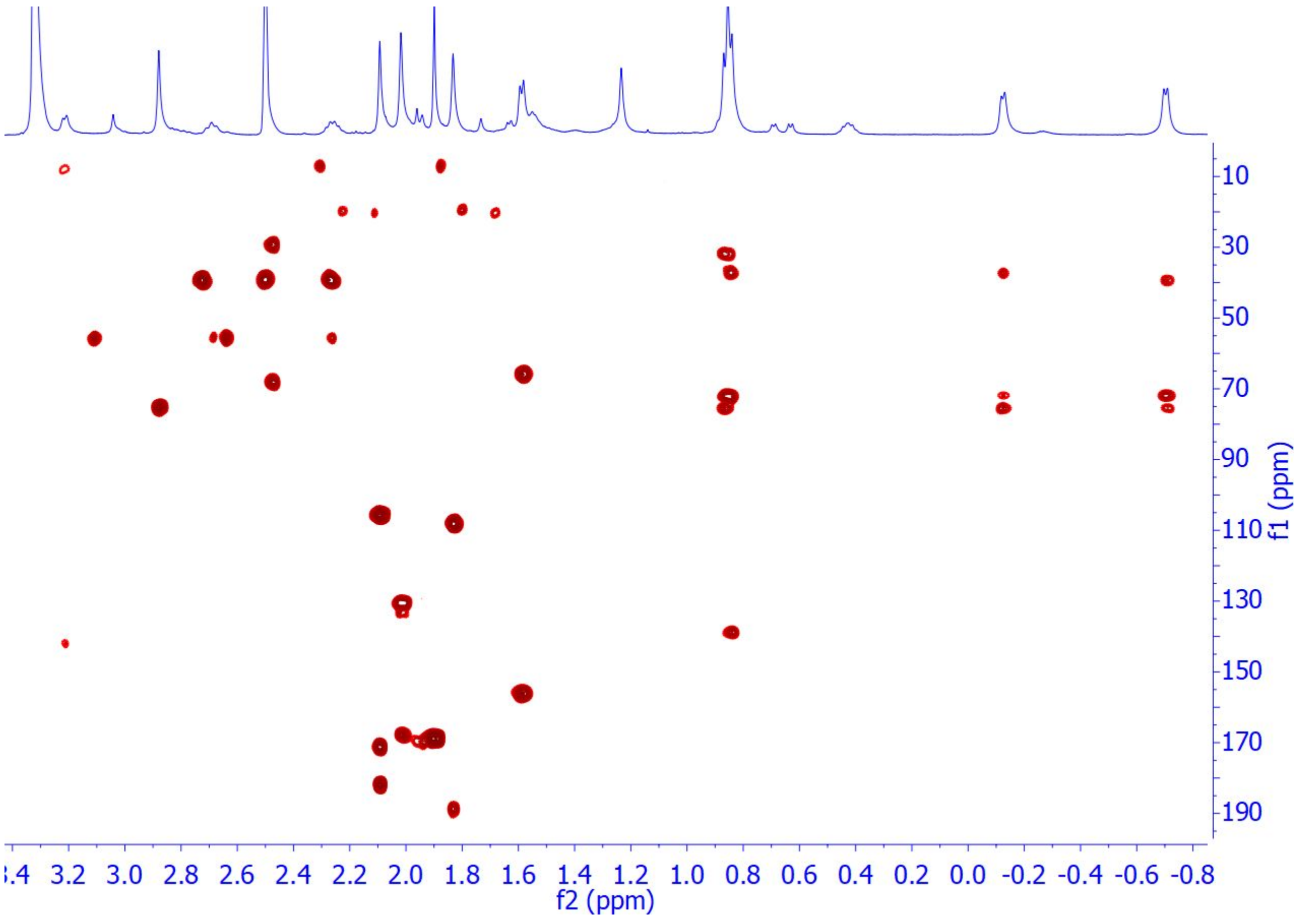

Figure S46. Expanded HMBC NMR spectrum of **5** in DMSO-d_6.

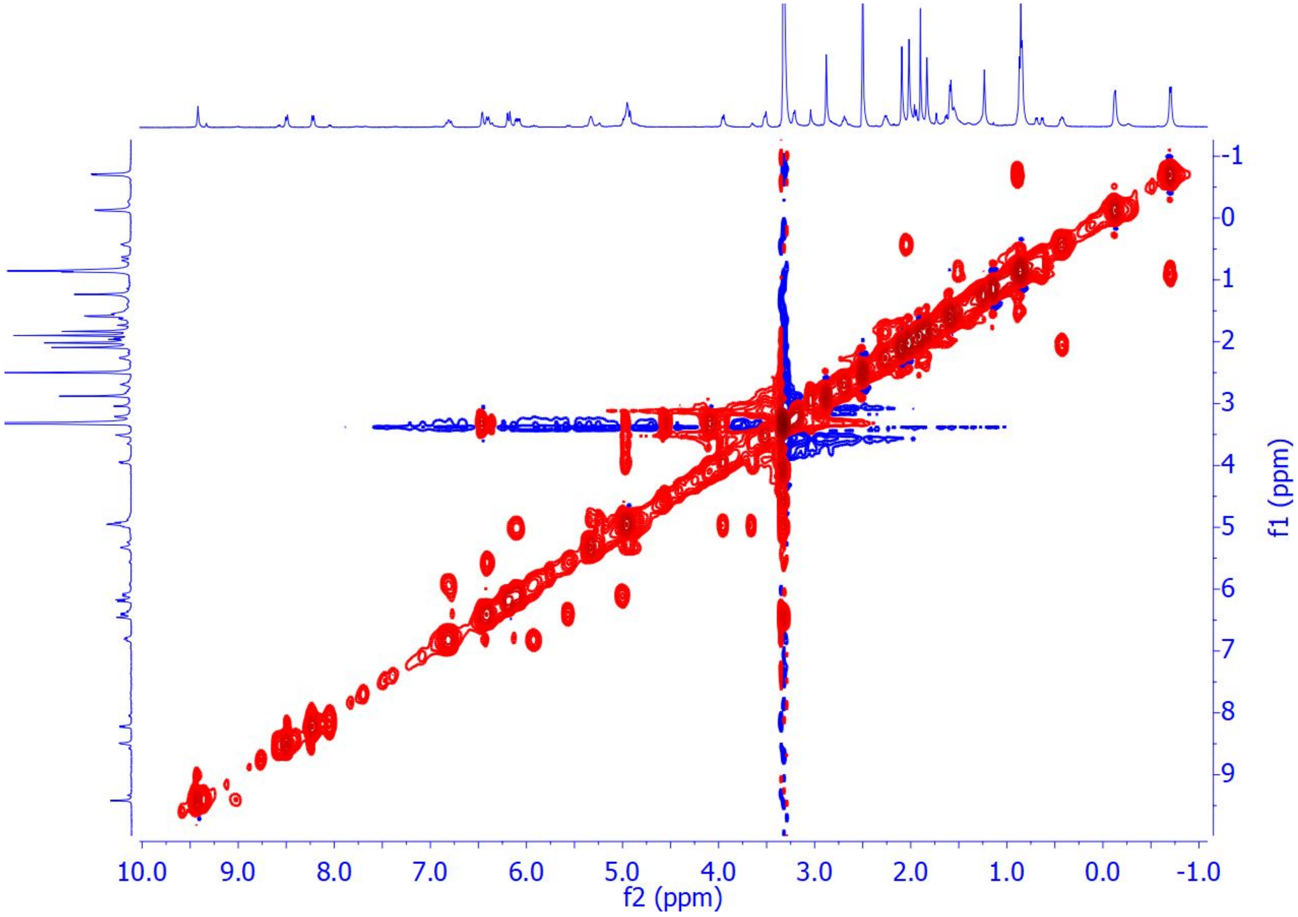

Figure S47. NOESY NMR spectrum of **5** in DMSO-d_6.

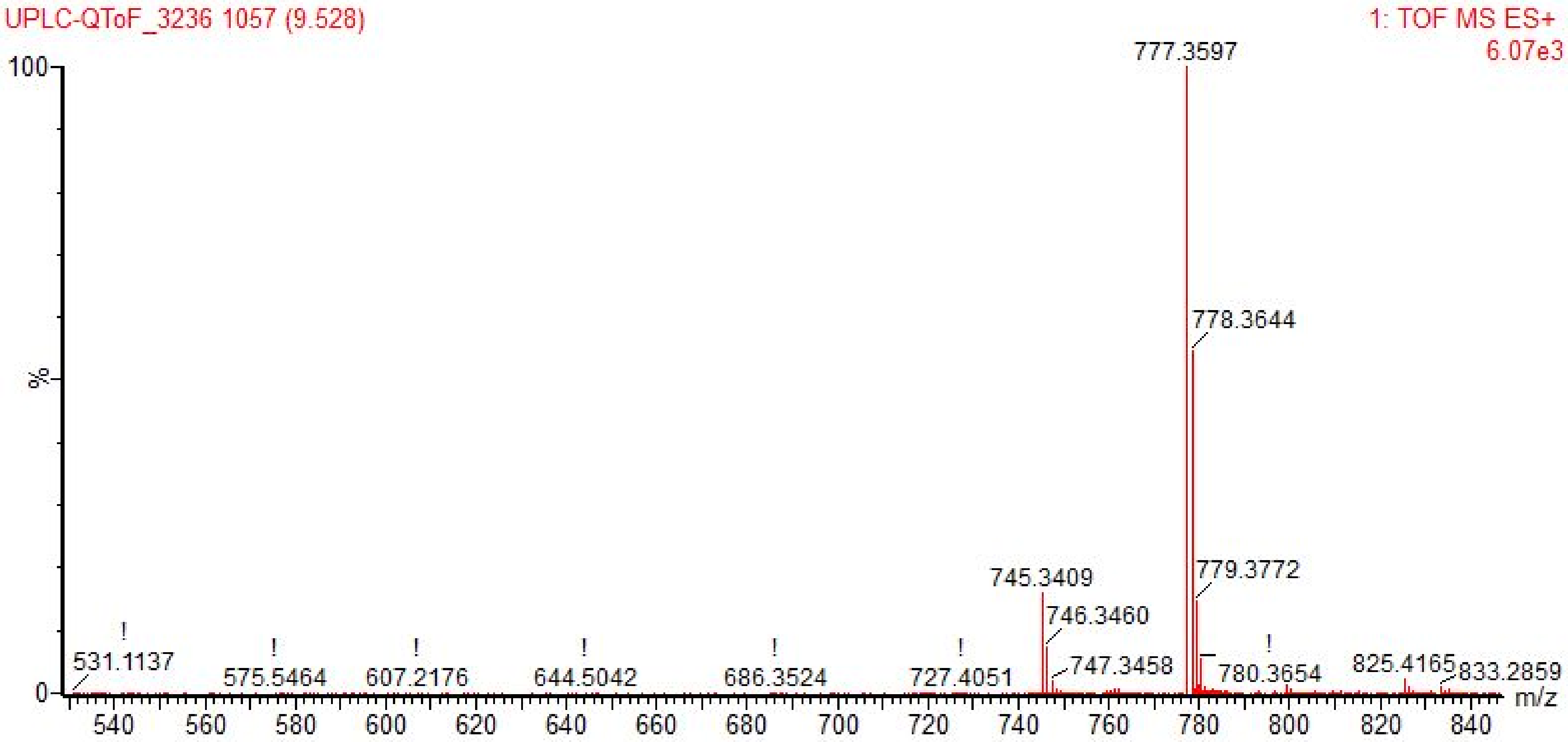

Figure S48. HRESIMS spectrum of **5**

Table S1. Calculated ^{13}C nuclear magnetic shielding (δ_C) using GIAO method with mPW1PW91/6–31 G(d,p) level of theory and ^{13}C NMR experimental data ($\delta_{C\ exp.}$), Root Mean Square Error (RMSE), Mean Absolute Error (MAE) and DP4+ probability values (%) for **1-5**.

**No.**	**1**			**2**			**3**			**4**					**5**		
	$\delta_{C\ exp.}$	$\delta_{C\ a}$	$\delta_{C\ b}$	$\delta_{C\ exp.}$	$\delta_{C\ a}$	$\delta_{C\ b}$	$\delta_{C\ exp.}$	$\delta_{C\ a}$	$\delta_{C\ b}$	$\delta_{C\ exp.}$	$\delta_{C\ a}$	$\delta_{C\ b}$	$\delta_{C\ c}$	$\delta_{C\ d}$	$\delta_{C\ exp.}$	$\delta_{C\ a}$	$\delta_{C\ b}$
**1**	172.8	159.4	157.3	173.0	159.3	158.0	nd	159.3	157.5	173.0	158.4	160.0	158.1	159.8	157.9	146.3	146.2
**2**	106.0	109.4	109.6	106.0	109.6	109.3	nd	109.5	109.6	105.6	106.2	107.8	107.0	108.2	111.0	113.7	114.2
**3**	188.3	186.4	185.6	188.1	186.7	188.3	188.6	186.5	185.4	188.2	187.0	185.5	186.0	185.3	129.1	137.5	137.6
**4**	76.5	76.8	77.1	76.4	76.7	77.4	76.2	76.8	77.1	76.4	79.6	77.5	79.2	77.9	127.2	117.3	117.7
**5**	105.7	110.4	109.6	105.7	110.3	110.2	nd	110.4	109.8	nd	110.8	110.5	111.3	110.7	nd	106.5	106.5
**6**	175.6	167.9	167.3	175.5	167.9	167.3	175.4	168.0	167.4	175.1	166.3	165.9	165.8	165.2	171.3	169.5	169.6
**7**	106.4	105.7	105.0	106.5	105.6	105.1	105.7	105.6	105.0	105.5	105.2	104.1	106.9	105.7	105.9	102.6	102.9
**8**	169.2	160.9	160.3	169.3	160.8	160.0	169.6	160.8	160.3	169.8	161.5	161.1	162.2	161.4	182.1	162.5	162.6
**9**	109.4	109.4	108.3	109.4	109.3	109.0	nd	109.4	108.3	109.3	111.1	106.4	111.9	107.7	116.3	111.2	111.6
**10**	145.1	145.9	147.4	145.2	146.1	145.7	nd	146.0	147.5	145.2	144.5	144.7	144.2	144.3	110.5	129.9	130.3
**11**	193.6	194.2	192.8	193.6	194.1	193.2	194.0	194.3	193.0	194.2	195.2	192.1	194.5	191.1	188.9	192.5	193.0
**12**	107.6	108.7	110.1	107.5	109.1	108.8	107.6	109.1	110.3	107.0	105.5	105.9	105.4	105.5	108.2	108.9	108.9
**13**	22.2	22.7	24.2	22.1	22.7	24.0	21.9	22.8	24.4	21.3	21.6	21.1	24.6	24.5	21.1	23.9	24.3
**14**	7.1	7.7	7.8	7.0	7.7	7.8	7.0	7.7	7.8	6.9	7.7	7.4	7.8	7.7	7.3	8.0	8.0
**15**	167.7	163.5	161.8	167.8	163.9	163.1	167.7	163.6	161.9	167.5	163.5	164.0	164.0	163.8	167.8	162.4	163.0
**16**	132.6	130.7	130.1	132.2	130.1	129.2	132.1	130.7	130.0	131.3	130.7	130.2	130.1	129.8	132.7	130.9	130.9
**17**	131.2	133.7	135.1	131.2	133.9	134.5	131.2	133.7	135.0	131.1	135.7	136.4	135.3	136.1	131.6	134.5	134.2
**18**	127.5	122.9	123.1	127.6	122.2	121.5	127.5	122.7	123.2	127.1	122.7	122.5	122.2	122.0	125.3	123.4	123.6
**19**	137.5	142.5	143.8	137.8	142.8	143.9	137.6	142.6	143.7	137.8	142.3	142.9	145.6	145.1	138.8	143.4	143.1
**20**	37.3	41.4	41.5	37.3	44.0	44.4	37.4	41.3	41.4	37.3	42.4	42.6	42.4	42.9	37.6	41.4	41.5
**21**	72.7	72.6	72.5	73.1	73.1	72.5	73.1	72.6	72.3	72.7	70.9	70.1	71.7	69.9	71.9	72.2	72.2
**22**	32.3	47.8	47.6	41.2	50.4	50.7	32.4	47.7	47.6	32.3	43.0	42.9	40.4	40.1	32.2	47.4	47.4
**23**	76.2	74.7	75.8	71.1	75.7	74.8	76.3	74.8	75.6	76.1	75.5	75.4	77.1	77.1	75.2	73.9	73.9
**24**	39.2	42.6	42.1	39.9	43.0	43.4	38.9	42.3	42.0	38.6	43.3	43.4	38.7	38.7	37.5	42.3	42.3
**25**	73.7	80.7	80.9	73.8	81.0	80.5	74.4	80.9	81.0	69.5	77.3	77.1	76.2	76.1	71.7	80.4	80.3
**26**	38.6	42.6	42.1	38.3	42.4	41.5	39.4	42.0	41.8	40.2	34.2	34.1	36.6	36.7	39.3	42.0	41.9
**27**	76.3	77.4	77.3	76.4	77.1	77.7	66.0	69.3	69.2	65.4	73.4	73.2	81.2	81.1	75.5	77.4	77.3
**28**	117.9	123.4	125.6	117.9	123.4	121.8	123.0	128.6	130.3	124.5	129.1	128.3	108.8	109.0	118.3	123.5	123.3
**29**	143.2	140.3	139.9	143.1	141.3	140.4	140.8	140.4	140.0	139.3	134.3	134.7	139.9	139.7	141.6	140.6	140.8
**30**	20.3	21.9	22.1	20.2	21.7	22.0	20.4	21.9	22.1	20.2	21.1	21.1	21.6	21.5	19.9	22.5	22.5
**31**	18.3	18.6	18.4	18.5	18.8	19.0	18.3	18.5	18.4	18.1	18.2	17.4	21.0	19.8	17.7	18.7	18.4
**32**	10.7	9.9	10.0	57.2	62.0	62.0	10.9	9.9	9.9	10.7	9.9	10.2	11.9	11.7	10.7	9.7	9.8

**33**	9.6	19.1	19.2	9.5	19.4	19.1	9.5	19.3	19.2	8.5	17.9	18.2	12.9	12.8	8.0	19.3	19.3
**34**	9.5	9.4	9.3	9.5	9.3	10.2	9.2	9.2	9.0	9.0	16.7	16.9	16.1	16.1	7.9	9.4	9.5
**35**	169.3	166.9	167.0	169.3	166.8	166.9	170.2	166.9	167.0						169.0	166.8	166.8
**36**	20.7	21.8	21.9	20.7	21.9	21.7	20.9	21.8	21.9						20.7	21.8	21.8
**37**	55.6	56.2	56.2	55.6	56.2	56.4									55.6	56.3	56.2
**1'**	54.9	42.4	50.5	55.1	43.1	44.4	55.0	42.7	50.2	55.1	43.3	56.2	43.3	56.1			
**2'**	205	213.8	205.5	205.0	213.4	212.5	205.2	213.8	205.5	205.1	213.4	204.7	213.5	204.5	156.5	164.2	164.1
**3'**	32.7	33.8	33.8	32.7	33.9	33.9	32.9	33.9	33.6	32.4	32.4	33.9	32.4	33.8	120.3	111.0	111.0
**4'**															138.7	138.1	138.6
**5'**															66.0	71.4	71.4
**6'**															22.6	24.8	25.1
**DP4+ (%)**		1.19	98.81		0.00	100.00		0.01	99.99		0.00	100.00	0.00	0.00		0.00	100.00
**RMSE**		5.16			5.20			4.89			5.26					6.82	
**MAE**		3.63			3.87			3.52			3.94					4.80	

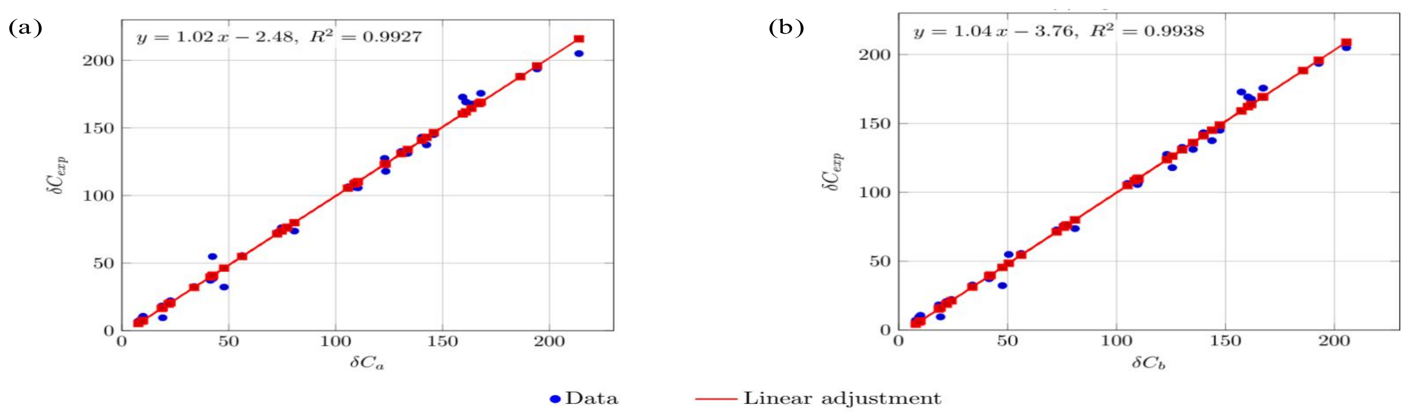

Figure S49. The correlation between the experimental chemical shift ($\delta_{\mathrm{C\ exp.}}$) versus the calculated magnetic isotropic shielding using GIAO method with *mPW1pw91/6-311G(d,p)* level of theory for (a) **1a** (4R) and (b) **1b** (4S).

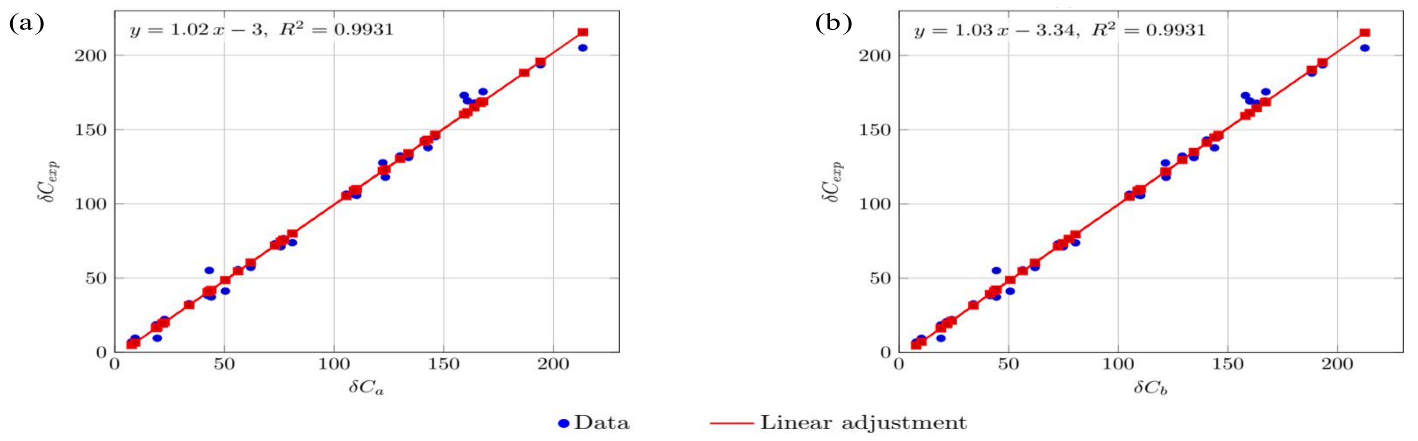

Figure S50. The correlation between the experimental chemical shift ($\delta_{C\ exp.}$) versus the calculated magnetic isotropic shielding using GIAO method with *mPW1pw91/6-311G(d,p)* level of theory for (a) **2a** (4R) and (b) **2b** (4S).

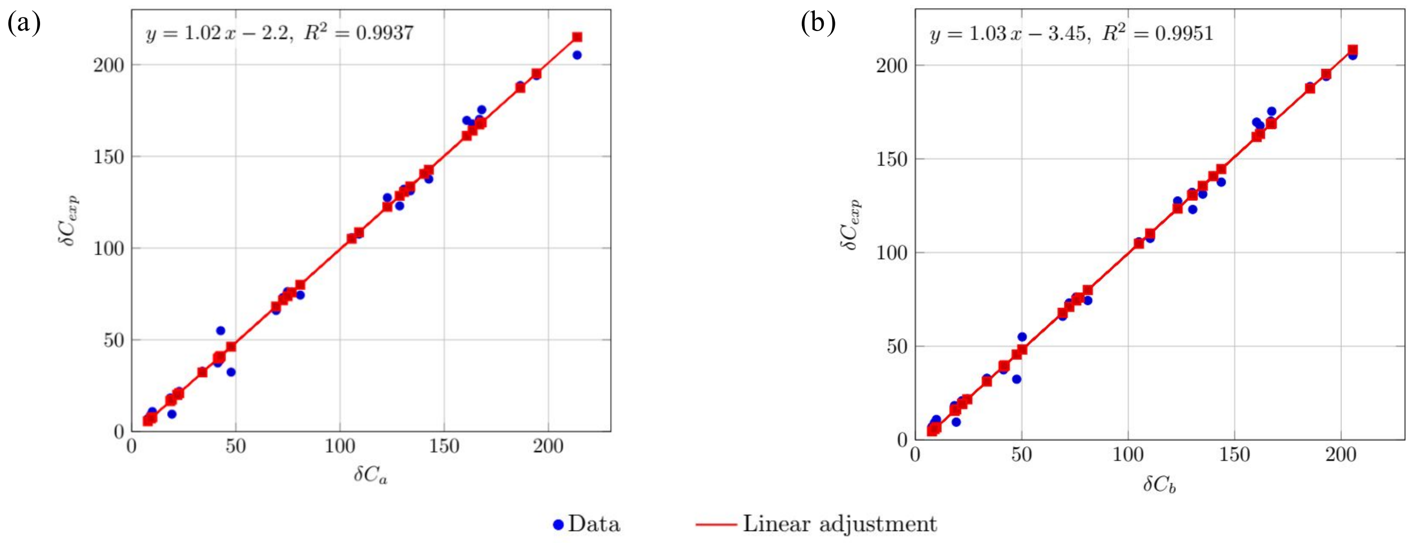

Figure S51. The correlation between the experimental chemical shift ($\delta_{\mathrm{C\ exp.}}$) versus the calculated magnetic isotropic shielding using GIAO method with *mPW1pw91/6-311G(d,p)* level of theory for (a) **3a** (4R) and (b) **3b** (4S).

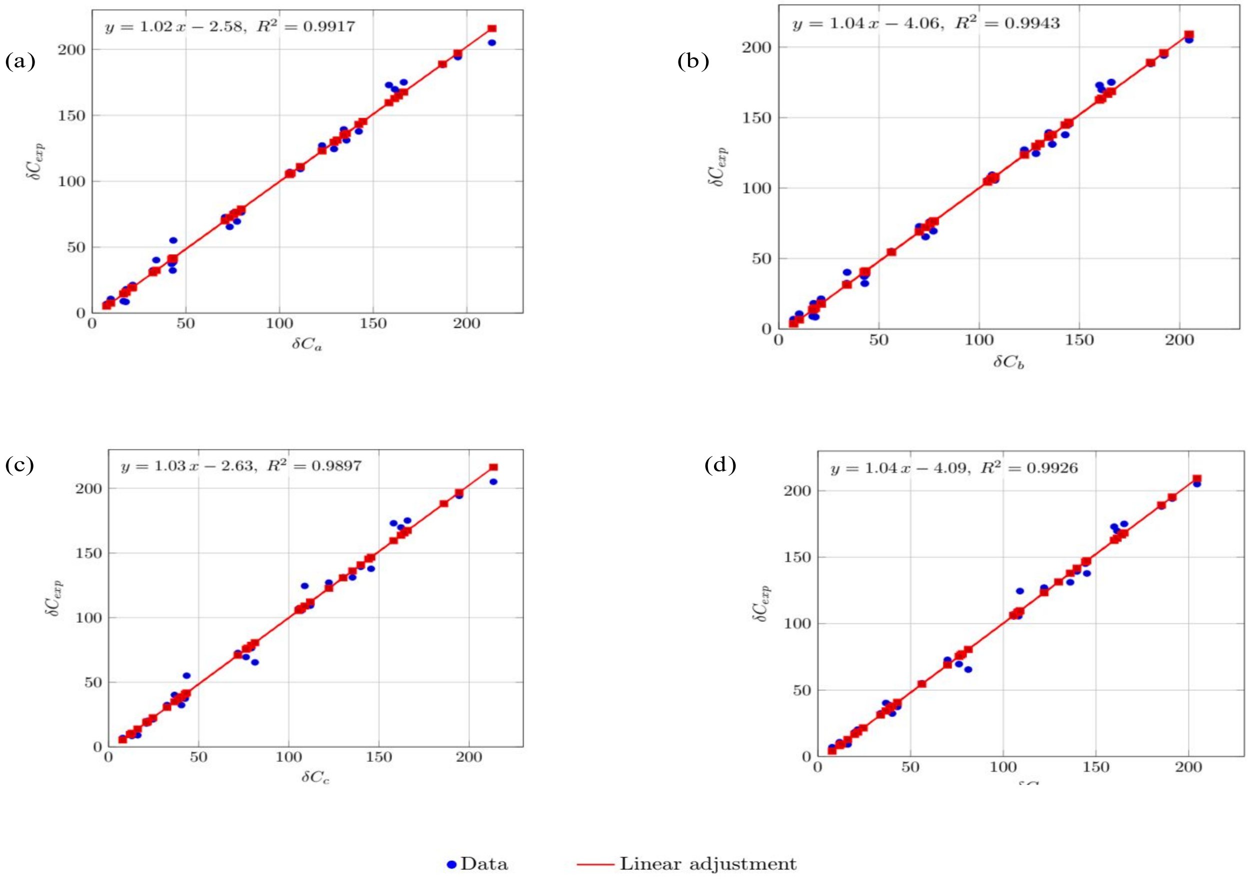

Figure S52. The correlation between the experimental chemical shift ($\delta_{C\ exp.}$) versus the calculated magnetic isotropic shielding using GIAO method with *mPW1pw91/6-311G(d,p)* level of theory for (a) **4a** (4R, 23R,27S), (b) **4b** (4S, 23R,27S), (c) **4c** (4R, 23S,27R), and (d) **4c** (4S, 23R,27S).

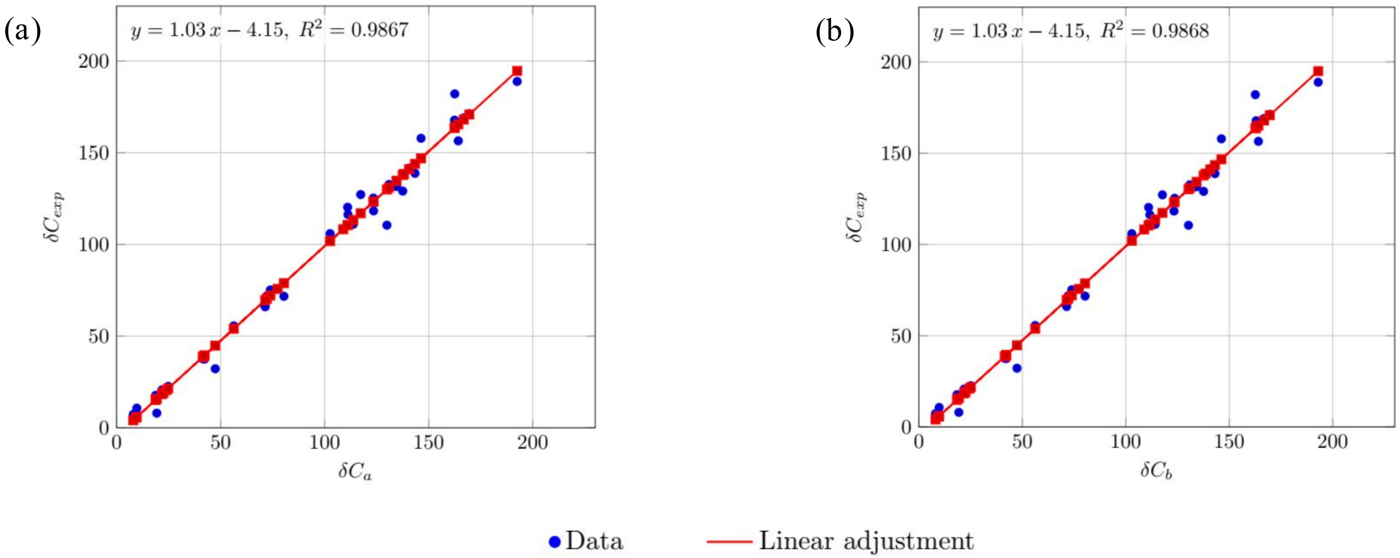

Figure S53. The correlation between the experimental chemical shift ($\delta_{C\,exp.}$) versus the calculated magnetic isotropic shielding using GIAO method with *mPW1pw91/6-311G(d,p)* level of theory for (a) **5a** (5'S) and (b) **5b** (5'R).

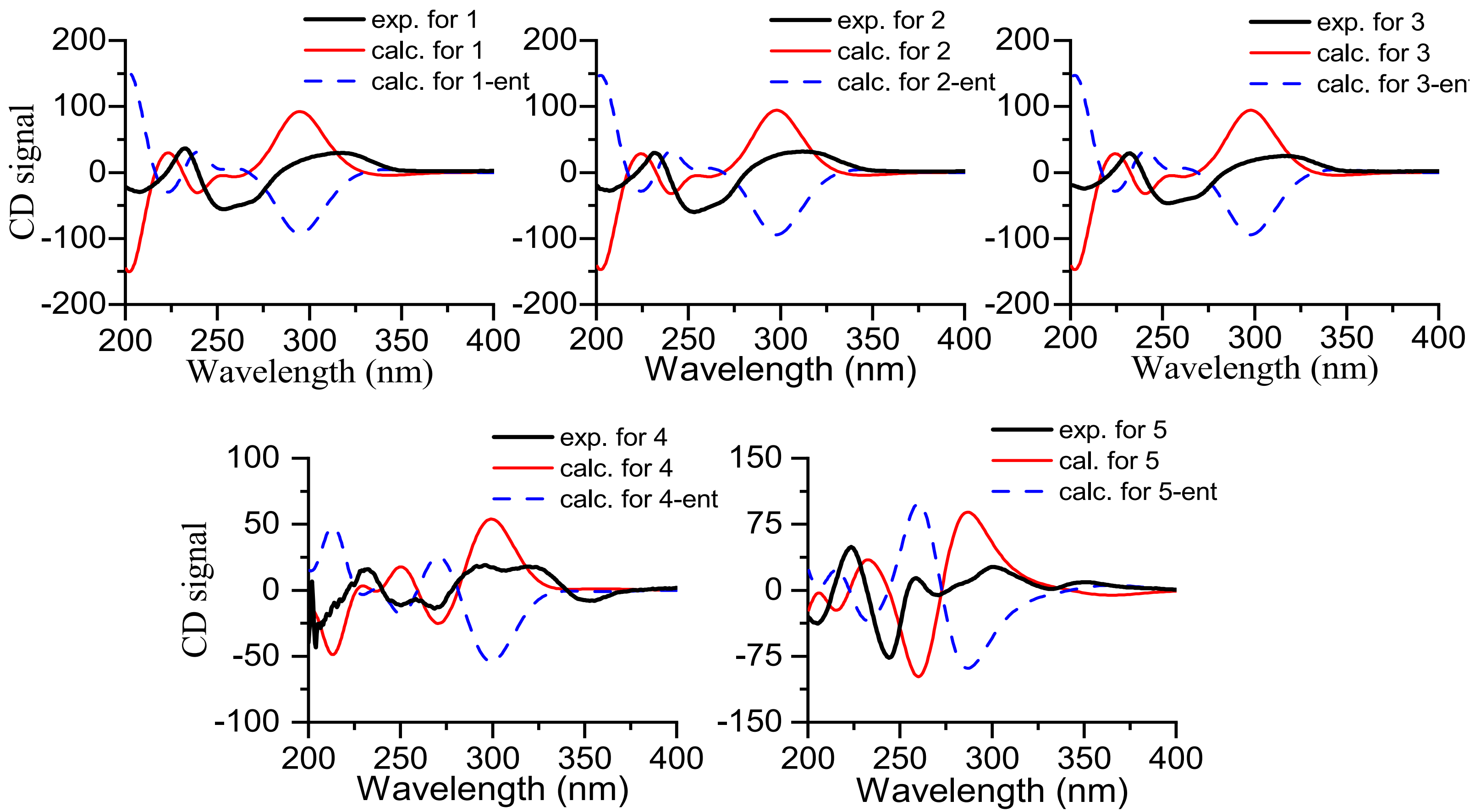

Figure S54. Experimental and calculated ECD spectra of **1-5** in methanol.
